# Changes in Gene Expression during Adaptation of *Listeria monocytogenes* to the Soil Environment

**DOI:** 10.1371/journal.pone.0024881

**Published:** 2011-09-23

**Authors:** Pascal Piveteau, Géraldine Depret, Barbara Pivato, Dominique Garmyn, Alain Hartmann

**Affiliations:** 1 Université de Bourgogne, UMR 1229, Dijon, France; 2 INRA, UMR 1229, Dijon, France; Max Planck Institute for Infection Biology, Germany

## Abstract

*Listeria monocytogenes* is a ubiquitous opportunistic pathogen responsible for listeriosis. In order to study the processes underlying its ability to adapt to the soil environment, whole-genome arrays were used to analyse transcriptome modifications 15 minutes, 30 minutes and 18 h after inoculation of *L. monocytogenes* EGD-e in soil extracts. Growth was observed within the first day of incubation and large numbers were still detected in soil extract and soil microcosms one year after the start of the experiment. Major transcriptional reprofiling was observed. Nutrient acquisition mechanisms (phosphoenolpyruvate-dependent phosphotransferase systems and ABC transporters) and enzymes involved in catabolism of specific carbohydrates (β-glucosidases; chitinases) were prevalent. This is consistent with the overrepresentation of the CodY regulon that suggests that in a nutrient depleted environment, *L. monocytogenes* recruits its extensive repertoire of transporters to acquire a range of substrates for energy production.

## Introduction

*Listeria monocytogenes* is a Gram positive opportunistic intracellular pathogen responsible of listeriosis, a severe condition in at-risk populations [1]. Over the past 30 years, *L. monocytogenes* has become a paradigm for the study of host/bacteria interactions [2]. The ecology of *L. monocytogenes* is complex, and this bacterium has the striking ability to switch from saprophytism to life within the host [3]. During saprophytic life, it is found in foodstuff and in the food processing environment [4], dairy farms [5], [6], faeces of animals [7], [8] and asymptomatic human carriers [9]. Natural environments where *L. monocytogenes* can be detected include water systems [10], vegetation [11], [12] and soil [8], [13]. Considering the ubiquitous nature of this bacterium, *L. monocytogenes* is a good model to study environmental adaptation of pathogenic bacteria. Determination of its genome gave insights on the genetic basis of this ubiquity. Indeed, the genome of *L. monocytogenes* includes large numbers of surface and secreted proteins, transporters and transcriptional regulators [14]. The role of several of these regulators has been deciphered. PrfA plays a key role during infection and regulates differentially 73 genes organized in three groups [15]. The alternative Sigma factor SigmaB regulates genes involved in adaptation to environmental changes [16], [17] and infection [18], [19]. CodY integrates energy and nutritional states of the cell to fine-tune its physiology to the environment [20]. Transcriptional regulation is complex, several operons overlap and are coregulated [21]–[23] suggesting that interconnected regulatory circuitry promotes rapid and fine-tuned adaptation to environmental changes. Complex transcriptional modifications occur in response to environmental stresses such as temperature variation [24], [25], osmolarity [17], [26], starvation [27] and during the onset of infection [28], [29]. Expression profiling during adaptation to foodstuff such as UHT milk [30] and cut cabbage [31] has been investigated; however transcriptional changes related to adaptation to the soil environment are still poorly documented. The objectives of this study were first of all to investigate growth and adaptation of *L. monocytogenes* to two soil environments and to decipher transcriptional modifications during the early stages of adaptation to these environments by mean of whole-genome microarrays.

## Results

### Bacterial growth in soil extracts and sterilized soils

**Figure 1:**
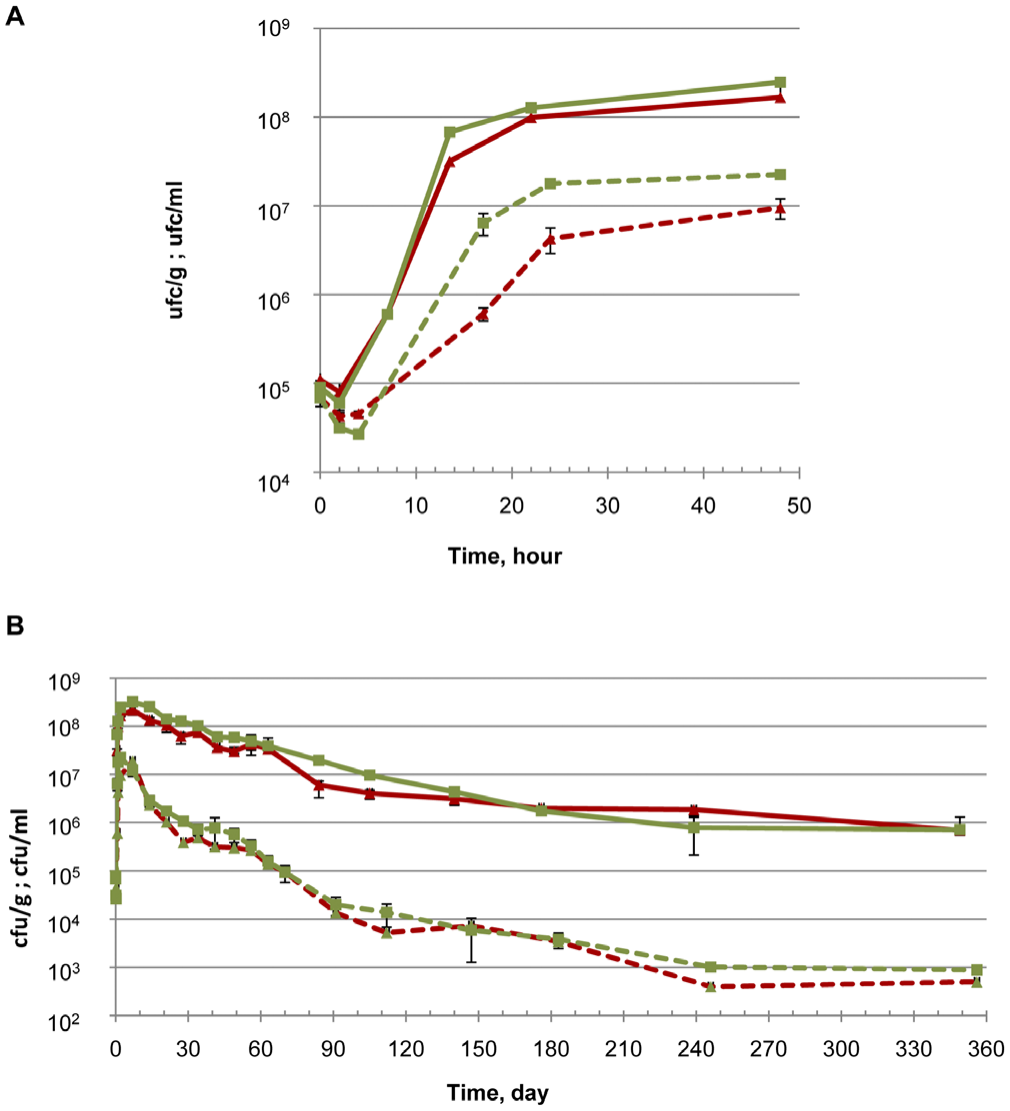
Growth and survival of *L. monocytogenes* EGD-e in soil microcosms (plain lines) and soil extracts (dashed lines) at 25°C. (A) Focus on the first two days of incubation. (B) one year incubation. Δ: control soil (TA); □: compost-amended soil (DA).

Growth and survival was followed during one year of incubation in soil microcosms and soil extracts (Figure 1). In soil microcosms, after inoculation, populations of *L. monocytogenes* EGD-e increased to over 10^+8^ cfu/g within 24 h. Growth stopped thereafter and large numbers were still detected during the rest of the incubation in soils. After 4 months, the population was around 10^+7^ cfu/g and 10^+6^ cfu/g were still numerated one year after the start of the experiments. A similar trend was observed in soil extracts but the population reached a maximum of 10^+7^ cfu/ml. The numbers decreased then. It was 10^+4^ cfu/ml after 4 month and around 10^+3^ cfu/ml were still detected at the end of the one-year period. Within the first week of incubation, the population of *L. monocytogenes* EGD-e was significantly higher in compost-amended soil (DA) and DA extract in comparison to control soil (TA) and TA extract respectively. Thereafter, differences were not statistically significant.

### Overview of transcriptome results

**Figure 2:**
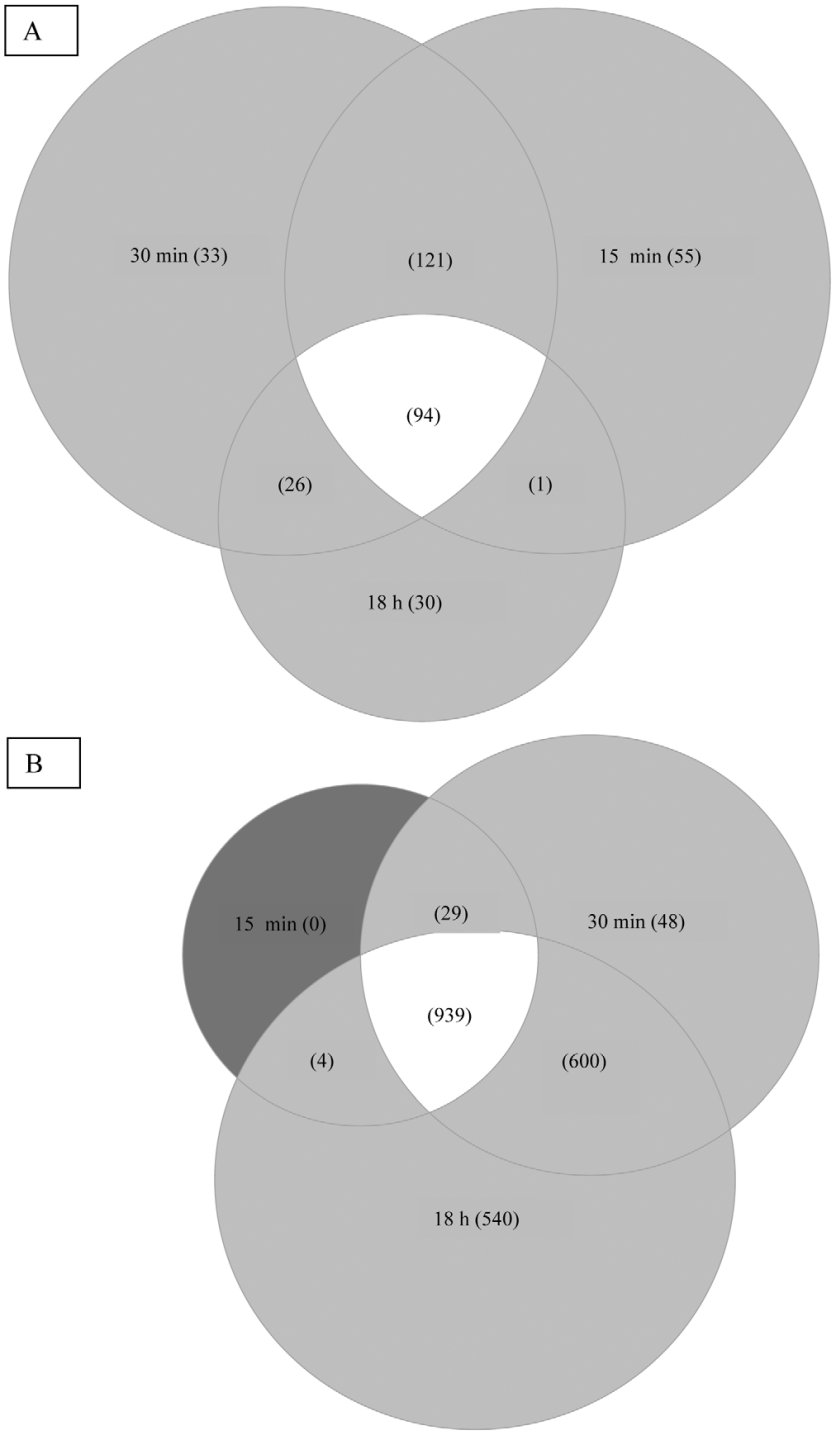
Venn diagramm of *L. monocytogenes* EGD-e genes with higher (A) and lower (B) transcript levels during 18 h incubation in soil extract.

Transcriptional microarray analysis was performed during growth of bacteria in soil extracts at 25°C. Incubation in soil extracts resulted in rapid and extensive transcriptional modifications. The number of genes whose transcript levels were significantly different (two-fold change, *P*<0.05) from T0 was 1237, 1883 and 2230 after 15 minutes, 30 minutes and 18 hours of incubation respectively (see list of genes in supplementary materials Tables S1 to S3). 271, 274 and 151 genes were up-regulated after 15 minutes, 30 minutes and 18 h respectively; among these, 94 were common to the three sampling times, 121 were common to time 15 min and 30 min while 26 were common to 30 min and 18 h (Figure 2A). The number of genes showing lower transcription increased gradually from 972 at 15 min to 2082 at 18 h (Figure 2B).

### Real time PCR quantification of selected transcripts

**Figure 3:**
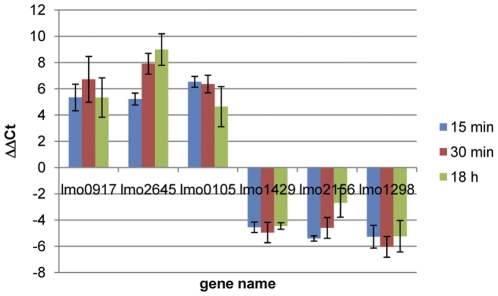
qRT-PCR quantification of selected genes with higher and lower transcript levels identified by microarray.

In order to confirm microarray results, six genes were selected for quantification of cDNA by real time PCR. Three of them (*lmo0917*, *lmo2645*, *lmo0105*) showed higher transcript levels in the microarray experiment and three (*lmo1429*, *lmo2156* and *lmo1298*) had lower transcript levels. The qRT-PCR confirmed microarray results (Figure 3). The transcript levels of *lmo0917*, *lmo2645* and *lmo0105* were significantly higher at 15 min, 30 min and 18 h than at time 0 (P<0.05). Conversely, normalised Ct values of *lmo1429*, *lmo2156* and *lmo1298* were significantly lower at 15 min, 30 min and 18 h than at time 0.

### Relative proportion of genes with higher and lower transcript levels within functional categories

**Figure 4:**
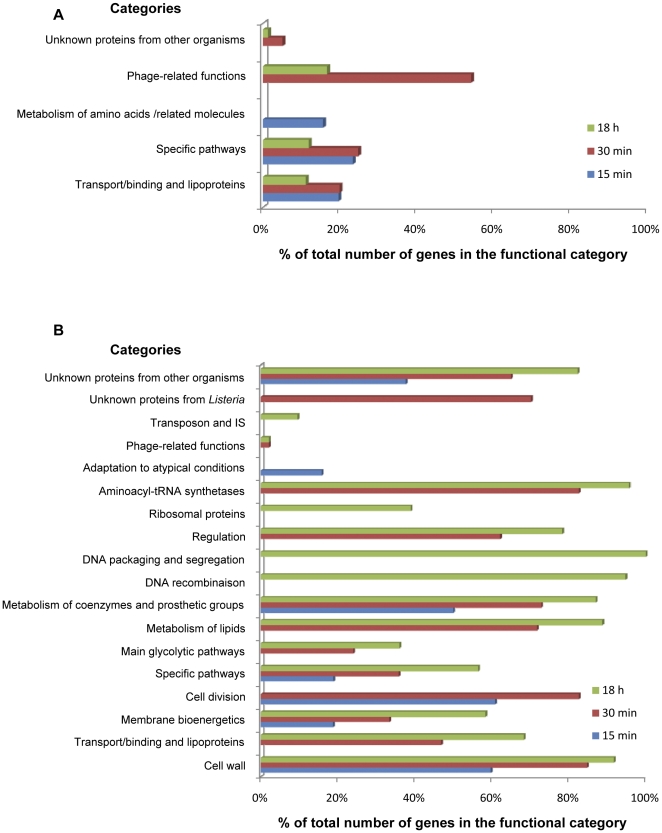
Functional categories identified after gene ontology among genes with higher (A) and lower (B) transcript levels.

The set of genes from each of these categories with higher transcript levels identified by gene ontology are reported in supplementary materials (Tables S4, S5, S6). The results of the analysis when functional categories were used as ontology terms are summarized figure 4. Genes most likely to be activated after 15 minutes of incubation in the soil environment (*P*<0.05) included 65 genes from category “Transport/binding proteins and lipoproteins” (1.2) and most of these (50 genes) encoded domains of phosphoenolpyruvate-dependent phosphotransferase systems (PTS). 51 genes belonged to the category “specific pathways” (2.1.1) and encoded enzymes involved in specific pathways of carbohydrates catabolism. Finally, 22 genes from category “metabolism of amino acids and related molecules” (2.2) were identified. After 30 min and 18 h, “transport/binding proteins and lipoproteins” (1.2) and “specific pathways” (2.1.1) were still represented as most likely significant terms as well as “similar to unknown proteins” (5.2) and “Phage-related functions” (4.3). The level of transcripts of twenty six of the 48 phage-related genes increased after 30 minutes of incubation. Among genes that showed higher levels of transcripts during the first 30 minutes of incubation, the proportion of genes encoding proteins involved in carbohydrate transport and metabolism, secondary metabolites, and to a lesser extent amino acid, nucleotide and coenzyme transport and metabolism was high. After 18 h, the relative importance of carbohydrate transport and metabolism as well as secondary metabolism increased.

Seven functional categories were determined as biologically significant within the set of genes with lower transcript levels at 15 min (Figure 4B). Three of them (“Cell wall”, “Membrane bioenergetics” and “cell division”) relate to cell envelope and cellular processes. Genes from categories “Specific pathways” and “Metabolism of coenzymes and prosthetic groups” are involved in intermediary metabolism. “Adaptation to atypical conditions” and “unknown proteins from other organisms” was identified as well. Five extra categories were detected after 30 minutes. These were related to cellular processes (“Transport/binding proteins and lipoproteins”), metabolism (“Main glycolytic pathways”, “Metabolism of lipids”), information pathways (“Regulation”, “Aminoacyl-tRNA synthetases”), “Phage-related functions” and “Unknown proteins from *Listeria*”. Finally, functional categories “DNA recombinaison”, “DNA packaging and segregation”, “Ribosomal proteins” and “Transposon and IS” were identified at 18 h. Overall, the transcription of 34%, 56% and 73% of total ORFs was lower after 15 min, 30 min and 18 h respectively.

### 94 genes showed higher transcript levels at each sampling time

**Figure 5:**
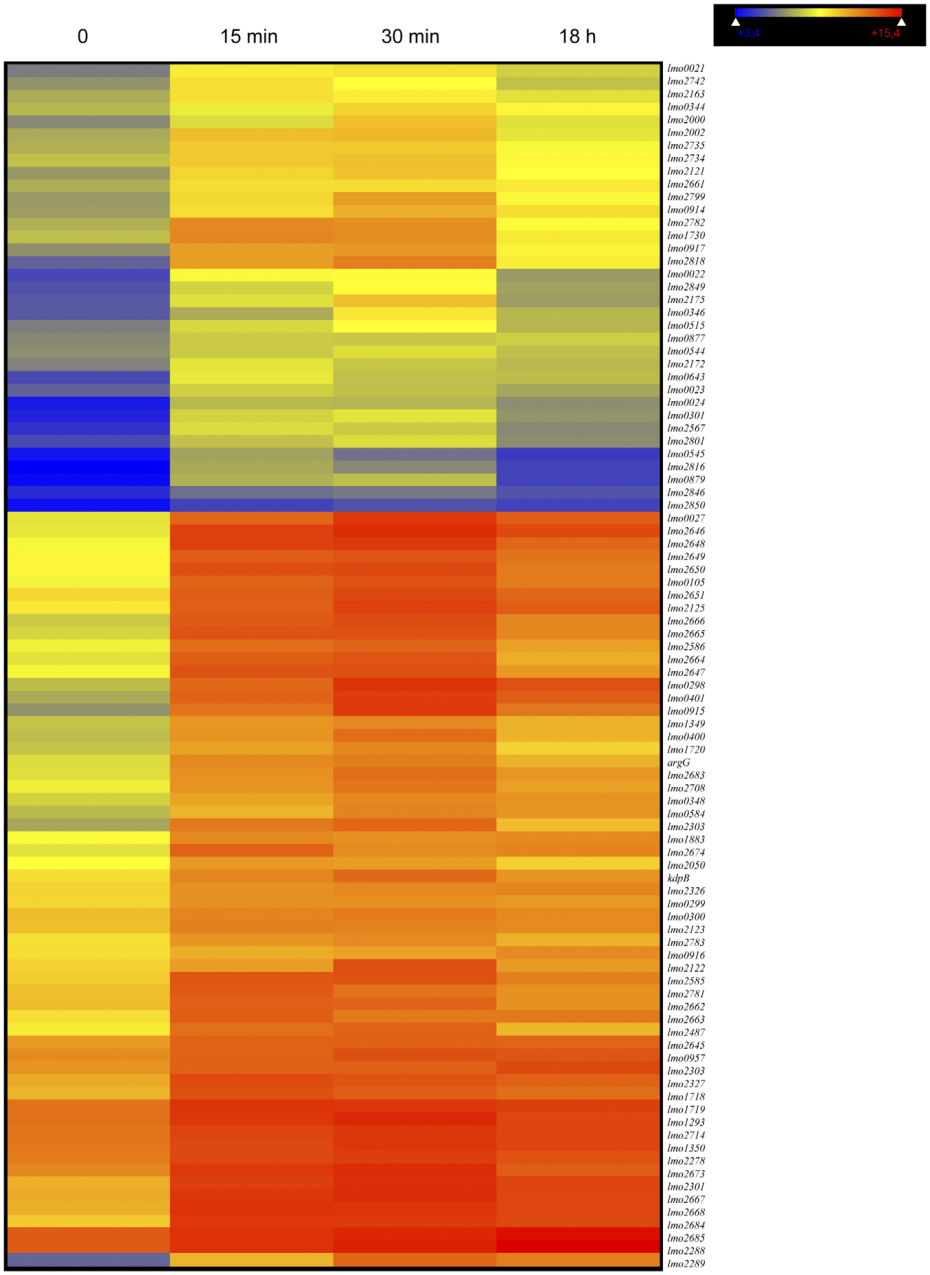
Expression levels of the 94 genes with higher transcript levels during incubation in the soil environment (TA). Each raw of coloured boxes represents one gene. Columns indicate sampling times (0, 15 min, 30 min and 18 h).

94 up-regulated genes (Figure 5) may be regarded as particularly important for saprophytic life in soils. Almost 40% of these genes belong to functional category 1.2 (37 genes) including genes coding for PTS systems, for example *lmo2649* (similar to hypothetical PTS enzyme IIC component), *lmo2782* (similar to PTS system, cellobiose-specific IIB component) and sugar transport proteins (*lmo2850*). Category 2.1.1 is highly represented as well (26 genes) including *lmo0105* (highly similar to chitinase B), *lmo1883* (similar to chitinases) and *lmo0917* (similar to β-glucosidase).

### Prevalence of genes from the CodY, PrfA, SigmaB and VirR regulons among the genes regulated in soil extracts

As these regulators are involved in the regulation of genes during transcriptional switches in response to environmental changes and in the regulation of virulence, we investigated whether genes of the CodY, PrfA, SigmaB and VirR regulons were overrepresented among genes with higher and lower transcript levels. Expression results were analysed with these four ontology terms. *P* values identified CodY as ontology term more likely to be significant among genes with higher transcript levels at 15 min (*P* = 1.28*10^−35^), 30 min (*P* = 1.17*10^−45^) and 18 h (*P* = 0.0000802). Twenty two genes of the CodY regulon were identified at time 15 min and 30 min (Figure 5; Table S7); at time 18 h, 15 genes of the CodY regulon were represented among the genes with higher transcript levels (Figure 5; Table S7). PrfA and SigmaB were not significant ontology terms at 15 min, 30 min and 18 h in the group of genes with higher transcript level.

The list of genes determined after gene ontology of the group of genes with lower transcript levels is presented in Supplementary materials (Table S7). The results, when considering these sets of genes, differed. Indeed, CodY regulated genes were also overrepresented among genes with lower transcript levels at 15 min (*P* = 2.11*10^−8^), 30 min (*P* = 3.34*10^−11^) and 18 h (*P* = 9.95*10^−35^), but PrfA was a significant term at 15 min (*P* = 0.0002) and 30 min (*P* = 0.022). Genes regulated by both PrfA and SigmaB were overrepresented at time 30 min (*P* = 5.11*10^−11^) and 18 h (*P* = 1.65*10^−7^) in the set of genes with lower transcript levels. The significance of ontology term SigmaB was high at time 18 h and over 50% of the genes of the SigmaB regulon were represented (113 out of 216 genes). Several SigmaB-regulated gene categories were represented and included cell wall biogenesis (*mreD*, *iap*, *spl*, *lmo2554*), cell division (*ftsE*, *ftsL*, *ftsX*, *ftsZ*, *minD*), cell cycle control (*smc*, *lmo1606*) and translation (*infC*, *tsf*, *frr*, ribosomal genes *rpsD*, *rpsI*, *rpsT*). Finally, the level of transcripts of the 17 genes of the VirR regulon was lower during incubation in the soil environment (Table S7), but the fold change of *virS* was not significant at times 15 min and 30 min, nor the fold changes of *dltB*, *dltC*, *dltD* and *virR* at time 15 min.

### Levels of transcripts of the major virulence determinants and internalin family of genes

This subset of genes was analysed to assess whether incubation in soil could affect virulence.

A focus on the major virulence determinants and internalin family is presented in supplementary material (). The fold changes of most of these genes were significantly greater than 2-fold down during incubation in soil except *plcB*, *inlA*, *inlE*, *inlH*, *lmo0610*, *lmo1289*, *lmo2027*, *lmo2445* and *lmo2470* which did not significantly vary during incubation and *inlB* for which a significant fold change (2.61 up) was observed only at time 15 minutes.

This decrease was significant from 15 minutes on for *iap*, *inlG*, *lmo0327*, *lmo0333*, *lmo0514*, *lmo0549*, *lmo0801*, *lmo1136*, *lmo2203* and *lmo2396*. The fold-change of *plcA*, *mpl*, *kat*, *lmo0331* and *lmo0409* was significant from 30 minutes on. Finally, variations regarding *prfA*, *hly*, *actA*, *sod*, *lmo0171*, *lmo1290* and *lmo2821* were significant at time 18 h only.

### Effect of soil amendment on the level of transcripts after incubation in soil extract

Minor differences were observed between the two soil extracts (Table S9). The number of genes at 2 fold change (DA versus TA) was 5, 27 and 19 at times 15 min, 30 min and 18 h respectively. Most of the differences between DA vs TA were close to the limit of significance (2 fold up or down), except *lmo0901* for which the level of transcript after 30 min incubation in DA was 7.495 higher than in TA. This gene is similar to a PTS cellobiose-specific IIC component.

## Discussion

Soil is an important reservoir of *Listeria monocytogenes* and a vector of transmission in agroecosystems and throughout the food chain [3]–[6], [8], [12], [13], [32]–[34]. Under our experimental conditions, long-term survival in soil was observed and significant bacterial loads were still detected one year after incubation. Soil amendment with green waste compost had only a slight effect on the growth and survival of *L. monocytogenes* as previously reported [13].

In order to sustain life, microorganisms have to cope with ever-changing environmental conditions. Integration of various environmental stimuli results in a coordinated response. Several traits of its genome support the ubiquitous nature of *L. monocytogenes*. Comparative genomics evidenced a large number of genes encoding transport proteins, especially PTS systems dedicated to carbohydrates uptake, and regulatory proteins including two component systems [14]. However, how this repertoire generates adaptation in specific habitats such as soils remains poorly documented.

Whole-genome microarray analysis evidenced massive transcriptional modifications at the beginning of the lag phase within 30 minutes of incubation in soil extracts. This is consistent with the need to regulate gene expression in order to respond to the physiological adjustments to the soil environmental conditions. One striking feature is the prevalence of nutrient acquisition mechanisms. Indeed, transcription of 22 of the 29 complete phosphoenolpyruvate-dependent phosphotransferase systems was higher in soil extracts and spanned the seven families of PTS (glucose/glucoside, mannose-fructose-sorbose, lactose-*N,N′*-diacetylchitobiose-*β*-glucoside, fructose-mannitol, galactitol, L-ascorbate and glucitol families) [35]. Interestingly, levels of transcripts of known activators of these PTS systems (*lmo0020*, *lmo0297*, *lmo0873*, *lmo0402*, *lmo0630*, *lmo2138*, *lmo0501*, *lmo2099* and *lmo2668*) were higher after incubation in soil extract. Several transporters including ABC transporters were over expressed including the gene cluster *lmo2121-lmo2126* and *lmo0278* whose function has been experimentally determined as uptake of maltose/maltodextrin [36]. Another interesting feature is the higher transcription of genes encoding enzymes involved in catabolism of specific substrates. β-glucosidases and chitinases were among genes with the highest transcript levels. Chitin hydrolysis and the functionality of two chitinases (*lmo0105* and *lmo1883*) have been demonstrated experimentally [37].

Overall, in the soil environment where nutrients are scarce, our results are consistent with the need to uptake a wide range of substrates as carbon, nitrogen and energy sources, and to synthesise specific enzymes required for their catabolism. These include the most abundant polymers in nature: cellulose and residues of plant material (fructose, sorbitol, cellobiose, β-glucosides, mannose, maltose, maltodextrin), exoskeleton of arthropods and cell wall of fungi (chitin). The ability of *L. monocytogenes* to acquire and utilise substrates from these various sources may be critical for its saprophytic life in soil. Overrepresentation of the CodY regulon confirms that *L. monocytogenes* probably has to face nutritional stresses resulting in a depletion of the energy level of the cell [20] and eventually a cessation of growth. Indeed, the cessation of growth is concomitant with a change of the transcriptional profile (18 h) under the control, at least partly, of SigmaB. Most of these repressed genes were also identified as targets of SigmaB during stationary phase, either directly or as an indirect control through regulation of the transcription of regulator genes and/or genes whose products affect RNA stability [16]. This is consistent and confirms that in response to nutrient limitation in soil, *L. monocytogenes* stops dividing in a manner similar to entry in stationary phase. SigmaB-dependent regulation is critical for adaptation to various environmental conditions such as osmotic, acid, ethanol and energy stresses [17], [26], [38], [39], temperature and growth phase [16], [24]. Furthermore, a role of SigmaB regulon in adaptation and survival in soil has been reported [40]. Our results also suggest that SigmaB regulation is important for saprophytic life in soil, further documenting the increasing body of evidence towards a central role of SigmaB in the transcriptional regulatory network of *L. monocytogenes* [21]–[23], [41].

Most of the virulence-associated and internalin genes were included in the set of genes with over 2-fold decrease during incubation except *plcB*, *inlA*, *inlE*, *inlH*, *lmo0610*, *lmo1289*, *lmo2027*, *lmo2445* and *lmo2470* for which fold changes were not significant and *inlB* for which fold changes were significant only at time 15 min. Furthermore, lower transcription of the genes of the VirR regulon was observed. These genes, mainly involved in modification of surface components, are important during infection [28], [42]. These results suggest that down regulation of virulence genes depends on stimuli from the soil environment such as the plant-derived disaccharide cellobiose [43] resulting in repression of the expression of virulence factors. The observed switch in the expression of internalin-coding genes upon environmental changes emphasizes the functional diversity of the internalin family, as it was previously proposed for their temperature-dependent expression [44]. The down regulation of genes involved in cell wall synthesis suggests modifications of the bacterial surface during growth and survival in soil.

Adaptation to specific niches requires deep transcriptional reshaping. During rapid growth in rich medium, SigmaB-regulation of genes involved in glucose and amino acid utilisation, stress responses, transcription factors and cell wall/membrane biogenesis was reported [16] while during heat-shock most of the differentially expressed genes were grouped in functional categories carbohydrate transport and metabolism, amino-acid transport and metabolism, transcription and translation [25]. *In vivo* in the intestinal lumen, the SigmaB regulon and genes involved in hypoxia and stress responses are up-regulated and PrfA-activated genes are up-regulated at the later stages of infection [28], [29]. Noteworthy, whatever the niche, *in vitro* [16], [25] *in vivo* [28], [29,] or in soil (this study), adaptation requires regulation of bacterial metabolism and modification of the bacterial surface.

Taken as a whole, our data evidenced a major transcriptional reprofiling during incubation in soil. We can speculate that, after a lag phase, this reprogramming may enable *Listeria monocytogenes* to generate energy for growth (over 3 log increase) from the available substrates ; furthermore, the long-term outcome of this reprogramming may be survival of large bacterial loads as evidenced after over one year incubation in soil. Overall, our results demonstrate the transcriptional basis of the capacity of *L. monocytogenes* to develop and remain in the soil environment and highlight the activation of genes encoding transport proteins, regulators and specific enzymes in order to colonize this habitat.

## Materials and Methods

### Soil characteristics and treatments

Loamy soil samples (pH 7.5 in water) were sampled in March 2009 at the experimental farming unit Qualiagro at Feucherolles (Yvelines, France). This facility managed by INRA since 1998 is dedicated to the study of the effects of compost or manure fertilisation on crop production and soil quality. For this reason, permits were not required for sample collection. The field was subdivided in four replicate blocks. Within each block, a wheat-maize rotation was conducted and fertilized with mineral nitrogen each year. Two soils from this experimental system were chosen. The first soil (Soil DA) was amended each second year at autumn by spreading co-composts of urban slurries and plant wastes and/or crushed pallets. The second soil was a control (Soil TA) with no organic fertilisation. Soils were collected in the 0–20 cm top-layer and four replicates from the four blocks were mixed to prepare soil microcosms or soil extracts.

Sterilized soil media were prepared from air-dried soil sterilized by γ-irradiation (45 kGy minimum) performed by Ionisos (Dagneux, France). Soil extracts were prepared according to Pochon and Tardieux method with some modifications. Briefly 500 g of soil were dispersed in 750 ml of water and autoclaved 1 hour at 130°C. Soil suspensions were centrifuged (10 000 g, 20 minutes), and supernatants were filtered on Whatman paper 3 MM. Then, these soil extracts were used as liquid growth media after autoclaving (20 minutes, 120°C).

### Growth kinetics in soil's extract and soil microcosm

*Listeria monocytogenes* strain EGD-e was used in this study. Pre-cultures were prepared by incubating statically for 16 h in 5 ml trypton soy broth (TSB; Biokar Diagnostics, Pantin, France) at 25°C. Then, 40 ml of fresh TSB were inoculated to an optical density (O.D_600 nm_) of 0.03 (1.5 10^7^ cfu/ml). This culture was grown under agitation (100 rpm; at 25°C) to an O.D_600 nm_ of 0.4. The culture was then centrifuged (5 600 g, 5 minutes at room temperature) and the bacterial pellet was suspended in NaCl 0.8%. Soil extracts (40 ml) and 35 g sterilized soil microcosms were inoculated with *L. monocytogenes* EGD-e to an initial concentration of 1×10^5^ CFU/ml (or CFU/g). Before bacterial inoculation, soil's humidity was adjusted to 16% (the soil humidity before drying and ionization).

Soil extracts and soil microcosms were incubated in the dark at 25°C under agitation (100 rpm) and statically respectively. *L. monocytogenes* EGD-e population was numerated by serial plating on tryptic soy agar (TSA) several times during the first 15 days, then once a week during 3 months and finally periodically up to one-year.

Three biological replicates were analysed. Cell numbers were compared by analysis of variance (ANOVA) using XLSTAT software (Addinsoft, 2009). Differences were considered statistically significant at the 0.05 probability level (*P* value).

### RNA isolation and cDNA synthesis

Bacterial cultures were prepared as described above except that bacterial pellets were suspended in 40 ml of soil extracts TA or DA. Inoculated soil extracts were incubated at 25°C, 100 rpm during 0, 15 minutes, 30 minutes and 18 hours. In order to stabilize RNA prior extraction, bacterial cultures were directly treated with RNAprotect bacterial reagent (QIAGEN, Courtaboeuf, France) according to the manufacturer's instructions. Three independent biological replicates were prepared and treated separately.

Bacteria were harvested by centrifugation (5500 g, 5 min), the pellet was suspended in 700 µl RLT buffer (Qiagen RNeasy kit) supplemented with 1% β-mercaptoethanol and 0.2 g of RNAse-free glass beads (100 µm) were added. Cells were disrupted mechanically in a Fast Prep (MP Bio, France) with 4 cycles (6 m/s; 30 s).

Total RNA was isolated using Qiagen RNeasy kit according to the manufacturer's protocol. An on-column DNase I (10 U, Quiagen) treatment was included before elution. Subsequently, 20 µg RNA were treated with 25 U RQ1 RNase-free DNase I (Promega, Charbonnière, France) in the presence of RNasin (40 U, Promega) and RNA was purified with Qiagen RNeasy MinElute cleanup kit according to the manufacturer's instructions. RNA purity and quality were assessed by spectrophotometer readings at 260 nm and by denaturing gel electrophoresis. RNA samples (10 µg each) were reverse transcribed using Superscript Double-Stranded cDNA Synthesis kit (Invitrogen, Cergy Pontoise, France) according to the manufacturer's instructions.

### Whole-genome microarrays and data analysis

A custom whole-genome microarray including all the 2857 annotated open reading frames (ORFs) of the genome of *Listeria monocytogenes* EGD-e was designed by NimbleGen Systems. Microarray hybridization, washes, raw data pre-processing and normalization was performed by NimbleGen Systems Inc (Madison, WI) according to their standard protocol.

DNASTAR ArrayStar software (Madison, WI) was used for the analysis of normalized results. T statistics and *P* values (*P*<0.05) were calculated to determine differentially expressed genes. The fold change was calculated by comparing results at 15 min, 30 min and 18 h with expression levels at time 0. Genes with at least 2-fold change were considered for interpretation in order to identify the relative importance of specific processes based on clusters of orthologous genes (COG) categories. Gene ontology was subsequently searched to identify relationships between genes with particular biological functions according to the functional classification established for the genus *Listeria* [14]. Finally SigmaB [16], codY [20] and PrfA [15] regulons were included as ontology terms in the analysis. Z-Scores, the standard statistical test used for hypergeometric distributions and the hypergeometric probability distribution (P-value) were calculated in order to determine the chance that a certain number of genes will be selected for any given gene ontology term.

For each condition, three sets of cDNAs were prepared from three sets of RNAs extracted from three independent biological repetitions and hybridised independently. Microarray data have been deposited to the Gene Expression Omnibus (https://www.ncbi.nlm.nih.gov/geo/).

### Real time PCR

In order to confirm results of the microarray analysis, transcript levels of three genes with higher and three genes with lower transcript levels were quantified using SYBR Green real time qRT-PCR as described previously [45]. Primers were designed using Primer3 (http://frodo.wi.mit.edu/primer3/) and tested with genomic DNA prior analysis. Three sets of cDNA originating from three sets of RNA extracted at each sampling time (15 min, 30 min and 18 h) were analysed. The double ΔCt method was used to analyse qRT-PCR results. The housekeeping gene *ldh* was selected as reference. Normalised Cts were compared to the calibrator condition “Time 0”.

## Supporting Information

Table S1 Genes over 2-fold change at time 15 minutes.
(PDF)

Table S2 Genes over 2-fold change at time 30 minutes.
(PDF)

Table S3 Genes over 2-fold change at time 18 hours.
(PDF)

Table S4 Gene ontology «functional categories » at time 15 minutes.
(PDF)

Table S5 Gene ontology «functional categories » at time 30 minutes.
(PDF)

Table S6 Gene ontology «functional categories » at time 18 h.
(PDF)

Table S7 List of genes with higher and lower transcript levels at time 15 min, 30 min and 18 h identified after gene ontology with ontology terms “CodY”, “PrfA”, “SigmaB” and “PrfA/SigmaB”.
(PDF)

Table S8 Transcript levels of the major virulence-associated genes and internalin family at time 15 min, 30 min and 18 h.
(PDF)

Table S9 Differences of gene expression during incubation in compost-amended (DA) and control (TA) soil extract.
(PDF)
