# Supplementary material for: Changes in Gene Expression during Adaptation of Listeria monocytogenes to the Soil Environment
Source: PLoS One. 2011 Sep 23;6(9):e24881. doi: 10.1371/journal.pone.0024881 (PMC3179493; doi:10.1371/journal.pone.0024881)
Supplement: Table S1 — Genes over 2-fold change at time 15 minutes. (PDF) [file pone.0024881.s001.pdf]

Table S1. Genes over 2-fold change at time 15 minutes.

LMO0003	3.509 down
LMO0008	2.044 down
LMO0009	2.421 down
LMO0010	2.130 down
LMO0013	2.265 down
LMO0017	2.607 down
LMO0020	2.705 up
LMO0021	11.202 up
LMO0022	19.035 up
LMO0023	15.139 up
LMO0024	19.291 up
LMO0025	2.012 up
LMO0027	18.344 up
LMO0029	2.469 down
LMO0030	2.300 down
LMO0033	2.197 down
LMO0034	3.182 down
LMO0035	2.436 down
LMO0038	2.182 up
LMO0039	2.448 up
LMO0042	3.013 down
LMO0046	2.201 down
LMO0050	2.458 down
LMO0051	2.030 down
LMO0055	3.381 down
LMO0071	2.432 down
LMO0072	3.150 down
LMO0073	3.185 down
LMO0074	2.706 down
LMO0075	2.743 down
LMO0077	2.515 down
LMO0079	2.075 down
LMO0083	2.381 up
LMO0102	2.104 down
LMO0105	34.138 up
LMO0106	4.663 down
LMO0109	2.592 up
LMO0110	2.542 up
LMO0111	3.297 down
LMO0112	3.074 down
LMO0113	3.519 up
LMO0114	2.460 up
LMO0132	2.284 down
LMO0133	2.363 up
LMO0134	2.804 up
LMO0135	2.227 down

LMO0136	2.138 down
LMO0137	2.141 down
LMO0138	2.760 down
LMO0139	2.880 down
LMO0140	2.211 down
LMO0142	2.600 down
LMO0143	2.273 down
LMO0144	4.034 down
LMO0145	4.683 down
LMO0146	2.493 down
LMO0151	7.528 down
LMO0152	8.297 down
LMO0153	7.921 down
LMO0154	5.646 down
LMO0159	2.523 down
LMO0160	2.601 down
LMO0167	2.016 down
LMO0168	2.846 down
LMO0176	3.646 down
LMO0181	2.024 up
LMO0185	4.702 down
LMO0186	6.153 down
LMO0187	3.731 down
LMO0189	2.820 down
LMO0190	4.450 down
LMO0192	2.797 down
LMO0198	3.283 down
LMO0199	4.068 down
LMO0213	2.469 down
LMO0218	2.672 down
LMO0219	2.858 down
LMO0221	2.056 down
LMO0222	2.514 down
LMO0223	2.544 down
LMO0238	3.616 down
LMO0239	3.039 down
LMO0240	2.751 down
LMO0241	2.985 down
LMO0242	2.245 down
LMO0243	3.546 down
LMO0244	6.199 down
LMO0245	9.249 down
LMO0246	4.269 down
LMO0247	2.750 down
LMO0248	2.261 down
LMO0252	4.319 down
LMO0254	2.328 down
LMO0256	2.770 down

LMO0262	2.389 down
LMO0265	2.270 up
LMO0269	6.234 down
LMO0272	3.492 down
LMO0273	4.570 down
LMO0274	3.439 up
LMO0278	3.867 up
LMO0279	2.946 up
LMO0280	2.062 up
LMO0282	4.449 down
LMO0283	4.326 down
LMO0284	6.166 down
LMO0285	8.352 down
LMO0286	9.158 down
LMO0287	2.045 down
LMO0288	3.196 down
LMO0289	2.139 down
LMO0293	4.388 down
LMO0294	4.031 down
LMO0297	2.332 up
LMO0298	13.358 up
LMO0299	13.398 up
LMO0300	11.617 up
LMO0301	17.073 up
LMO0302	3.316 down
LMO0303	2.224 down
LMO0304	2.162 down
LMO0306	2.827 down
LMO0307	2.452 down
LMO0315	2.650 down
LMO0316	2.716 down
LMO0319	2.193 up
LMO0320	2.862 down
LMO0323	3.948 up
LMO0326	2.128 down
LMO0327	2.152 down
LMO0333	2.304 down
LMO0344	2.317 up
LMO0345	2.632 up
LMO0346	4.276 up
LMO0347	3.007 up
LMO0348	4.088 up
LMO0349	4.717 up
LMO0350	4.933 up
LMO0351	7.184 up
LMO0357	2.209 up
LMO0364	6.892 down
LMO0365	4.697 down

LMO0366	2.521 down
LMO0367	2.127 down
LMO0371	4.514 down
LMO0377	2.191 up
LMO0383	7.233 up
LMO0384	6.414 up
LMO0385	3.668 up
LMO0386	3.020 up
LMO0390	3.298 down
LMO0394	12.132 down
LMO0395	5.441 down
LMO0396	2.478 down
LMO0398	4.494 up
LMO0399	3.859 up
LMO0400	6.875 up
LMO0401	19.291 up
LMO0402	2.473 up
LMO0403	2.220 down
LMO0404	2.221 down
LMO0412	2.266 down
LMO0420	2.374 down
LMO0430	2.786 down
LMO0434	2.617 up
LMO0435	2.024 down
LMO0436	3.392 down
LMO0440	2.302 down
LMO0442	2.592 down
LMO0449	2.653 down
LMO0450	3.094 down
LMO0451	2.309 down
LMO0452	2.286 down
LMO0453	2.535 down
LMO0454	2.872 down
LMO0455	2.795 down
LMO0465	4.445 down
LMO0468	2.033 down
LMO0469	9.239 down
LMO0470	4.272 down
LMO0471	2.647 up
LMO0472	2.304 down
LMO0473	2.633 down
LMO0476	2.032 down
LMO0477	10.879 down
LMO0478	11.019 down
LMO0479	3.846 down
LMO0480	2.571 down
LMO0482	4.403 down
LMO0485	10.003 down

LMO0486	3.765 down
LMO0489	2.498 down
LMO0492	2.389 down
LMO0494	2.031 down
LMO0495	3.847 down
LMO0496	2.259 up
LMO0497	3.891 down
LMO0501	3.185 up
LMO0502	4.322 up
LMO0503	2.766 up
LMO0504	2.543 up
LMO0509	4.562 down
LMO0510	3.862 down
LMO0511	3.546 down
LMO0513	2.037 down
LMO0514	12.522 down
LMO0515	2.990 up
LMO0517	10.613 up
LMO0519	5.133 down
LMO0520	2.636 down
LMO0522	2.070 down
LMO0532	2.016 down
LMO0536	5.450 up
LMO0537	2.238 down
LMO0541	2.141 down
LMO0543	3.289 up
LMO0544	14.110 up
LMO0545	11.899 up
LMO0546	3.676 up
LMO0547	2.346 down
LMO0549	2.378 down
LMO0550	3.280 down
LMO0551	2.253 down
LMO0552	2.923 down
LMO0559	3.199 down
LMO0573	2.711 down
LMO0581	4.708 down
LMO0582	4.708 down
LMO0583	2.505 down
LMO0584	2.929 up
LMO0585	4.437 down
LMO0586	5.393 down
LMO0588	4.266 down
LMO0589	2.366 down
LMO0597	3.181 down
LMO0598	4.602 down
LMO0599	2.946 down
LMO0600	2.748 down

LMO0601	2.829 down
LMO0602	3.455 up
LMO0604	5.633 down
LMO0608	2.661 up
LMO0609	3.923 down
LMO0611	3.367 down
LMO0616	2.225 down
LMO0622	2.752 down
LMO0623	2.091 down
LMO0629	2.153 up
LMO0630	6.815 up
LMO0631	4.174 up
LMO0632	4.421 up
LMO0633	3.209 up
LMO0634	3.111 up
LMO0635	6.685 down
LMO0636	3.157 down
LMO0637	2.145 down
LMO0638	5.909 down
LMO0640	2.295 up
LMO0643	6.213 up
LMO0644	2.912 down
LMO0645	3.518 down
LMO0655	2.078 down
LMO0656	7.545 down
LMO0657	2.521 down
LMO0658	2.360 down
LMO0660	2.877 up
LMO0664	2.568 down
LMO0665	2.417 down
LMO0666	2.468 down
LMO0667	2.126 down
LMO0668	2.239 down
LMO0672	5.841 down
LMO0678	5.114 down
LMO0679	6.455 down
LMO0680	7.974 down
LMO0681	4.252 down
LMO0682	4.254 down
LMO0683	7.304 down
LMO0684	5.106 down
LMO0685	2.726 down
LMO0686	2.007 down
LMO0691	2.438 down
LMO0692	2.286 down
LMO0693	3.365 down
LMO0694	2.698 down
LMO0696	2.062 down

LMO0720	2.378 up
LMO0721	3.170 down
LMO0725	5.403 down
LMO0726	7.710 down
LMO0727	5.306 down
LMO0728	2.639 down
LMO0733	2.967 down
LMO0739	2.293 down
LMO0740	2.510 down
LMO0745	2.074 down
LMO0748	2.857 down
LMO0749	2.381 down
LMO0751	2.232 down
LMO0752	2.188 down
LMO0755	2.550 down
LMO0756	2.175 down
LMO0765	3.248 down
LMO0766	2.356 down
LMO0767	2.490 down
LMO0769	2.114 down
LMO0770	3.038 down
LMO0771	5.064 down
LMO0772	5.292 down
LMO0773	2.140 down
LMO0777	4.742 down
LMO0778	6.784 down
LMO0779	3.129 down
LMO0781	2.470 up
LMO0785	2.380 down
LMO0787	2.362 down
LMO0788	2.743 down
LMO0790	3.220 down
LMO0791	4.192 down
LMO0793	2.929 down
LMO0795	3.445 down
LMO0796	2.209 up
LMO0801	2.114 down
LMO0802	6.802 down
LMO0806	2.856 down
LMO0814	4.934 down
LMO0815	3.643 down
LMO0816	3.250 down
LMO0817	3.603 down
LMO0818	2.731 down
LMO0831	2.002 down
LMO0833	3.444 down
LMO0834	5.139 down
LMO0835	4.256 down

LMO0836	4.512 down
LMO0837	6.276 down
LMO0839	2.428 down
LMO0840	2.814 down
LMO0841	2.146 down
LMO0842	3.804 down
LMO0845	2.400 down
LMO0847	6.130 down
LMO0848	6.606 down
LMO0850	3.389 down
LMO0851	3.385 down
LMO0852	4.030 down
LMO0853	2.574 down
LMO0854	2.626 down
LMO0855	2.141 down
LMO0857	2.258 down
LMO0866	3.447 down
LMO0867	3.549 down
LMO0869	3.084 up
LMO0870	2.569 up
LMO0873	3.685 up
LMO0874	4.725 up
LMO0875	4.492 up
LMO0876	3.433 up
LMO0877	2.685 up
LMO0878	3.251 up
LMO0879	3.296 up
LMO0882	3.777 down
LMO0883	3.055 down
LMO0884	2.762 down
LMO0885	2.055 down
LMO0887	2.033 down
LMO0888	2.288 down
LMO0889	2.331 down
LMO0890	2.486 down
LMO0891	2.562 down
LMO0899	4.584 down
LMO0903	4.318 down
LMO0908	2.516 down
LMO0912	2.190 down
LMO0914	23.186 up
LMO0915	21.540 up
LMO0916	35.521 up
LMO0917	60.712 up
LMO0918	3.780 up
LMO0920	4.366 down
LMO0921	2.406 down
LMO0922	2.299 down

LMO0927	2.662 down
LMO0928	3.205 down
LMO0934	2.375 down
LMO0935	2.288 down
LMO0939	3.601 down
LMO0948	2.062 down
LMO0950	2.063 down
LMO0951	2.891 down
LMO0952	3.760 down
LMO0957	2.650 up
LMO0958	2.791 up
LMO0959	2.480 down
LMO0960	2.797 down
LMO0961	2.188 down
LMO0966	2.075 down
LMO0967	2.301 down
LMO0968	2.705 down
LMO0969	2.368 down
LMO0974	2.336 down
LMO0975	2.440 down
LMO0981	2.947 down
LMO0983	2.023 up
LMO0988	2.492 down
LMO0997	5.180 up
LMO0998	9.823 down
LMO0999	2.254 down
LMO1000	3.393 down
LMO1001	4.605 down
LMO1004	2.981 down
LMO1006	2.350 down
LMO1008	3.517 down
LMO1009	2.102 down
LMO1011	2.195 down
LMO1013	2.348 down
LMO1014	6.032 down
LMO1015	3.471 down
LMO1016	2.767 down
LMO1017	2.370 down
LMO1024	3.873 down
LMO1025	3.111 down
LMO1030	2.591 down
LMO1037	4.734 down
LMO1039	2.171 down
LMO1043	2.465 up
LMO1044	2.254 up
LMO1045	2.683 up
LMO1046	3.348 up
LMO1047	3.054 up

LMO1048	3.366 up
LMO1049	2.105 up
LMO1056	3.257 down
LMO1064	2.205 down
LMO1065	2.172 down
LMO1066	2.807 down
LMO1067	3.764 down
LMO1068	3.070 up
LMO1070	2.327 down
LMO1071	3.099 down
LMO1073	6.184 down
LMO1074	3.767 down
LMO1076	2.989 down
LMO1077	3.372 down
LMO1078	5.478 down
LMO1079	5.005 down
LMO1080	2.486 down
LMO1086	2.042 down
LMO1088	2.107 down
LMO1089	2.425 down
LMO1090	2.581 down
LMO1091	2.370 down
LMO1092	2.200 down
LMO1093	2.058 down
LMO1096	2.070 down
LMO1113	2.735 up
LMO1114	2.655 up
LMO1119	2.340 down
LMO1120	3.832 down
LMO1122	2.143 down
LMO1123	3.107 down
LMO1128	2.926 down
LMO1129	2.568 down
LMO1130	2.261 down
LMO1131	4.210 down
LMO1132	2.304 down
LMO1134	2.422 down
LMO1135	3.397 down
LMO1136	4.214 down
LMO1137	3.292 up
LMO1138	2.280 up
LMO1140	2.466 up
LMO1141	2.797 down
LMO1144	2.662 down
LMO1145	2.415 down
LMO1146	2.527 down
LMO1147	2.732 down
LMO1148	2.747 down

LMO1149	3.784 down
LMO1169	2.994 down
LMO1170	3.780 down
LMO1175	3.298 down
LMO1177	2.301 down
LMO1178	2.340 down
LMO1179	2.545 down
LMO1180	2.381 down
LMO1181	2.968 down
LMO1182	2.489 down
LMO1183	2.966 down
LMO1184	2.819 down
LMO1185	3.055 down
LMO1186	2.107 down
LMO1197	2.697 down
LMO1198	2.494 down
LMO1199	2.731 down
LMO1200	3.493 down
LMO1201	3.828 down
LMO1202	4.182 down
LMO1203	2.896 down
LMO1204	4.132 down
LMO1205	3.111 down
LMO1206	2.808 down
LMO1207	2.412 down
LMO1208	2.191 down
LMO1210	3.807 down
LMO1211	3.581 down
LMO1216	3.566 down
LMO1221	2.613 down
LMO1222	2.609 down
LMO1223	3.665 down
LMO1228	2.343 down
LMO1229	2.764 down
LMO1230	2.334 down
LMO1231	2.175 down
LMO1235	3.519 down
LMO1236	4.194 down
LMO1237	8.958 down
LMO1238	6.185 down
LMO1239	11.778 down
LMO1240	6.501 down
LMO1241	2.366 up
LMO1243	2.256 down
LMO1245	4.452 down
LMO1246	3.852 down
LMO1247	4.311 down
LMO1248	13.616 down

LMO1249	6.265 down
LMO1250	3.120 down
LMO1251	11.667 down
LMO1252	3.142 down
LMO1254	2.167 up
LMO1256	2.998 up
LMO1270	2.071 down
LMO1271	2.228 down
LMO1272	2.051 down
LMO1274	2.590 down
LMO1275	5.129 down
LMO1276	2.425 down
LMO1284	2.931 down
LMO1286	3.083 down
LMO1291	2.067 down
LMO1292	4.130 down
LMO1293	2.898 up
LMO1294	2.894 down
LMO1296	3.670 down
LMO1297	2.812 down
LMO1298	9.011 down
LMO1300	2.214 down
LMO1306	3.641 down
LMO1307	4.683 down
LMO1308	2.947 down
LMO1309	4.427 down
LMO1310	7.753 down
LMO1311	7.994 down
LMO1312	4.096 down
LMO1313	3.569 down
LMO1314	2.639 down
LMO1315	4.527 down
LMO1316	3.548 down
LMO1317	2.381 down
LMO1321	2.350 down
LMO1322	2.396 down
LMO1323	2.049 down
LMO1326	2.398 down
LMO1327	2.743 down
LMO1328	3.994 down
LMO1329	2.270 down
LMO1330	2.739 down
LMO1331	7.413 down
LMO1334	2.328 down
LMO1335	3.221 down
LMO1336	2.253 down
LMO1337	2.100 down
LMO1347	2.109 down

LMO1348	4.328 up
LMO1349	7.360 up
LMO1350	3.829 up
LMO1351	2.558 down
LMO1352	2.933 down
LMO1353	11.720 down
LMO1355	2.261 down
LMO1356	3.398 down
LMO1357	2.281 down
LMO1358	2.062 down
LMO1359	2.290 down
LMO1364	2.281 down
LMO1365	3.805 down
LMO1366	2.299 down
LMO1369	4.663 down
LMO1370	3.928 down
LMO1375	2.132 down
LMO1384	4.383 down
LMO1385	3.101 down
LMO1395	3.899 down
LMO1396	4.582 down
LMO1400	2.186 down
LMO1401	2.205 down
LMO1403	2.034 down
LMO1409	4.185 down
LMO1410	3.031 down
LMO1411	2.708 down
LMO1416	3.125 down
LMO1417	4.568 down
LMO1418	4.637 down
LMO1419	5.928 down
LMO1420	5.369 down
LMO1424	2.571 down
LMO1428	4.482 down
LMO1429	14.980 down
LMO1430	4.569 down
LMO1431	9.421 down
LMO1436	2.494 down
LMO1437	3.121 down
LMO1438	2.024 down
LMO1440	12.921 down
LMO1445	2.219 down
LMO1446	4.930 down
LMO1447	4.601 down
LMO1449	2.553 down
LMO1450	2.290 down
LMO1459	2.319 down
LMO1461	2.108 down

LMO1463	2.005 down
LMO1464	2.129 down
LMO1468	2.760 down
LMO1476	3.034 down
LMO1478	2.215 down
LMO1479	2.189 down
LMO1480	2.907 down
LMO1481	2.004 down
LMO1483	3.737 down
LMO1485	5.664 down
LMO1486	3.828 down
LMO1487	2.930 down
LMO1488	2.925 down
LMO1489	2.929 down
LMO1490	3.206 down
LMO1491	3.753 down
LMO1492	7.301 down
LMO1494	2.414 down
LMO1495	2.302 down
LMO1496	3.420 down
LMO1497	2.329 down
LMO1498	3.794 down
LMO1499	3.812 down
LMO1500	4.812 down
LMO1504	2.477 down
LMO1506	2.648 down
LMO1511	2.069 down
LMO1512	2.752 down
LMO1513	3.451 down
LMO1515	8.155 down
LMO1519	2.346 down
LMO1520	3.509 down
LMO1521	2.988 down
LMO1523	2.194 down
LMO1524	2.853 down
LMO1528	2.123 down
LMO1530	2.141 down
LMO1532	2.033 down
LMO1536	4.297 down
LMO1537	5.266 down
LMO1538	3.467 up
LMO1545	2.486 down
LMO1546	2.460 down
LMO1548	2.303 down
LMO1549	4.071 down
LMO1550	2.702 down
LMO1551	2.507 down
LMO1552	2.338 down

LMO1555	2.429 down
LMO1556	3.287 down
LMO1557	3.721 down
LMO1558	5.100 down
LMO1559	2.410 down
LMO1568	2.446 down
LMO1575	2.033 down
LMO1576	2.457 down
LMO1577	2.766 down
LMO1580	2.291 up
LMO1581	2.125 down
LMO1582	2.499 down
LMO1584	3.597 down
LMO1585	5.861 down
LMO1589	2.314 up
LMO1590	3.770 up
LMO1591	4.317 up
LMO1592	2.437 down
LMO1593	2.692 down
LMO1594	3.013 down
LMO1596	2.052 down
LMO1598	2.417 down
LMO1600	2.976 down
LMO1604	2.677 down
LMO1613	2.392 down
LMO1614	5.984 down
LMO1615	4.707 down
LMO1616	6.312 down
LMO1617	2.428 down
LMO1618	3.688 down
LMO1621	2.090 down
LMO1622	3.548 down
LMO1623	2.053 down
LMO1624	3.009 down
LMO1625	4.061 down
LMO1626	3.201 down
LMO1635	4.718 down
LMO1641	2.655 down
LMO1645	2.461 down
LMO1646	3.944 down
LMO1647	4.149 down
LMO1648	2.769 down
LMO1653	2.522 down
LMO1654	3.192 down
LMO1655	2.683 down
LMO1656	4.361 down
LMO1657	2.290 down
LMO1658	2.066 down

LMO1660	2.973 down
LMO1661	2.061 down
LMO1662	3.258 down
LMO1663	2.626 down
LMO1664	6.152 down
LMO1665	3.807 down
LMO1668	2.375 down
LMO1669	4.403 down
LMO1670	4.128 down
LMO1671	7.071 down
LMO1676	2.620 down
LMO1677	2.224 down
LMO1681	2.070 down
LMO1682	3.387 down
LMO1687	2.301 down
LMO1688	2.825 down
LMO1689	5.568 down
LMO1691	2.090 down
LMO1692	3.012 down
LMO1693	2.161 down
LMO1695	2.275 down
LMO1696	7.540 down
LMO1697	3.757 down
LMO1698	4.409 down
LMO1706	2.387 down
LMO1707	2.842 down
LMO1708	3.061 down
LMO1709	2.752 down
LMO1710	3.987 down
LMO1711	2.147 down
LMO1713	2.270 down
LMO1715	3.437 down
LMO1718	4.872 up
LMO1719	5.354 up
LMO1720	5.168 up
LMO1722	3.432 down
LMO1723	3.356 down
LMO1724	3.749 down
LMO1725	3.938 down
LMO1729	2.676 up
LMO1730	11.181 up
LMO1731	5.217 up
LMO1732	2.885 up
LMO1735	2.620 down
LMO1736	2.476 down
LMO1737	2.989 down
LMO1738	15.882 down
LMO1739	17.405 down

LMO1740	22.949	down
LMO1742	2.352	down
LMO1743	2.237	down
LMO1744	2.395	down
LMO1747	2.714	down
LMO1748	7.390	down
LMO1749	5.809	down
LMO1750	2.870	down
LMO1751	2.006	down
LMO1753	3.353	down
LMO1761	6.186	down
LMO1762	3.448	down
LMO1764	3.540	up
LMO1765	3.910	up
LMO1766	2.463	up
LMO1767	2.135	up
LMO1768	2.079	up
LMO1775	2.626	down
LMO1776	3.248	down
LMO1778	2.040	down
LMO1779	6.367	down
LMO1787	3.138	down
LMO1791	4.467	up
LMO1796	3.154	down
LMO1797	2.448	down
LMO1801	2.058	down
LMO1802	2.674	down
LMO1804	4.145	down
LMO1805	6.963	down
LMO1806	2.165	down
LMO1807	2.214	down
LMO1808	2.505	down
LMO1809	6.084	down
LMO1810	6.984	down
LMO1811	2.786	down
LMO1812	3.705	down
LMO1813	2.214	down
LMO1814	2.183	down
LMO1815	3.442	down
LMO1816	5.296	down
LMO1817	3.009	down
LMO1826	2.289	down
LMO1827	2.291	down
LMO1828	2.781	down
LMO1830	2.456	up
LMO1831	3.397	up
LMO1832	2.143	up
LMO1837	2.343	down

LMO1838	2.984 down
LMO1839	4.038 down
LMO1840	7.510 down
LMO1841	6.120 down
LMO1842	3.192 down
LMO1847	2.498 down
LMO1848	3.162 down
LMO1849	3.240 down
LMO1850	2.441 down
LMO1851	2.984 down
LMO1864	4.202 down
LMO1865	5.108 down
LMO1866	2.828 down
LMO1869	2.439 down
LMO1870	32.338 down
LMO1871	2.483 down
LMO1872	3.211 down
LMO1875	2.835 down
LMO1878	2.936 down
LMO1879	2.421 up
LMO1880	3.321 down
LMO1883	7.157 up
LMO1884	3.259 down
LMO1885	5.388 down
LMO1887	2.787 down
LMO1889	2.603 down
LMO1890	2.493 down
LMO1891	2.976 down
LMO1892	2.485 down
LMO1900	2.099 down
LMO1901	2.117 down
LMO1902	2.994 down
LMO1903	4.508 down
LMO1908	2.175 down
LMO1909	3.043 down
LMO1910	3.988 down
LMO1911	11.150 down
LMO1915	3.247 down
LMO1918	4.125 down
LMO1920	2.196 down
LMO1923	2.436 down
LMO1925	3.310 down
LMO1926	2.766 down
LMO1927	2.849 down
LMO1928	2.657 down
LMO1932	2.557 down
LMO1933	2.415 down
LMO1936	2.406 down

LMO1937	3.738	down
LMO1940	2.462	down
LMO1944	4.301	down
LMO1950	2.049	down
LMO1951	2.230	down
LMO1952	2.536	down
LMO1957	3.930	down
LMO1958	4.631	down
LMO1959	4.594	down
LMO1960	2.883	down
LMO1961	2.335	down
LMO1977	3.349	down
LMO1978	2.582	down
LMO1981	2.074	down
LMO1983	2.438	up
LMO1984	4.107	up
LMO1985	3.951	up
LMO1986	4.822	up
LMO1987	4.040	up
LMO1988	3.317	up
LMO1989	3.209	up
LMO1990	3.662	up
LMO1991	3.346	up
LMO1992	2.197	up
LMO1994	2.044	down
LMO1996	2.081	down
LMO1997	5.834	up
LMO1998	4.561	up
LMO1999	7.159	up
LMO2000	10.236	up
LMO2001	6.605	up
LMO2002	8.015	up
LMO2003	3.852	up
LMO2004	3.296	up
LMO2018	2.159	down
LMO2019	2.060	down
LMO2020	3.173	down
LMO2021	3.653	down
LMO2025	2.451	up
LMO2029	2.170	down
LMO2034	2.351	down
LMO2035	2.956	down
LMO2037	2.147	down
LMO2038	2.841	down
LMO2039	2.564	down
LMO2040	3.369	down
LMO2041	6.144	down
LMO2042	7.077	down

LMO2044	3.355 down
LMO2045	4.645 down
LMO2046	5.505 down
LMO2047	2.211 down
LMO2048	5.943 down
LMO2049	2.015 down
LMO2050	2.984 up
LMO2051	2.360 down
LMO2052	4.913 down
LMO2053	5.209 down
LMO2056	3.270 down
LMO2058	3.068 down
LMO2059	4.014 down
LMO2061	3.542 down
LMO2062	5.271 down
LMO2063	3.027 down
LMO2070	2.659 down
LMO2073	2.520 down
LMO2075	2.008 down
LMO2076	2.562 down
LMO2077	3.007 down
LMO2078	3.417 down
LMO2079	3.768 down
LMO2080	2.747 down
LMO2081	2.314 down
LMO2082	2.335 down
LMO2086	3.227 down
LMO2087	3.817 down
LMO2088	4.671 down
LMO2090	4.707 up
LMO2091	5.961 up
LMO2096	2.339 down
LMO2099	2.610 up
LMO2100	6.071 down
LMO2103	2.096 down
LMO2104	5.258 down
LMO2105	5.288 down
LMO2106	3.608 down
LMO2111	2.298 down
LMO2112	2.489 down
LMO2114	4.994 down
LMO2115	3.891 down
LMO2116	2.151 down
LMO2117	2.244 down
LMO2118	4.136 down
LMO2121	9.319 up
LMO2122	9.721 up
LMO2123	11.361 up

LMO2124	10.151 up
LMO2125	12.023 up
LMO2126	2.538 up
LMO2127	3.305 down
LMO2128	2.639 down
LMO2129	3.034 down
LMO2131	2.445 down
LMO2132	2.231 up
LMO2135	2.749 up
LMO2136	5.115 up
LMO2137	5.052 up
LMO2138	4.163 up
LMO2144	3.381 down
LMO2156	22.391 down
LMO2158	2.627 up
LMO2159	2.336 up
LMO2160	2.970 up
LMO2161	3.610 up
LMO2162	5.404 up
LMO2163	6.220 up
LMO2170	2.132 up
LMO2171	5.184 up
LMO2172	4.861 up
LMO2175	7.305 up
LMO2177	2.993 down
LMO2179	2.167 down
LMO2187	3.628 down
LMO2197	10.774 down
LMO2202	6.457 down
LMO2203	2.941 down
LMO2207	3.858 down
LMO2210	5.331 up
LMO2211	2.272 down
LMO2212	3.047 down
LMO2213	2.514 up
LMO2218	2.134 down
LMO2223	2.287 down
LMO2225	2.072 down
LMO2233	3.575 down
LMO2239	5.567 down
LMO2240	5.039 down
LMO2241	5.780 down
LMO2243	3.378 down
LMO2244	3.122 down
LMO2248	2.120 down
LMO2249	2.430 down
LMO2259	2.339 down
LMO2260	2.885 down

LMO2275	2.055 up
LMO2278	2.376 up
LMO2288	2.081 up
LMO2299	2.068 up
LMO2301	2.367 up
LMO2302	2.345 up
LMO2303	2.517 up
LMO2304	2.212 up
LMO2306	2.004 up
LMO2320	2.267 up
LMO2321	2.086 up
LMO2325	2.108 up
LMO2326	2.120 up
LMO2327	2.387 up
LMO2332	2.442 up
LMO2337	2.123 down
LMO2345	2.266 down
LMO2346	2.141 down
LMO2347	3.018 down
LMO2348	3.934 down
LMO2349	5.199 down
LMO2350	7.589 down
LMO2351	13.626 down
LMO2352	23.562 down
LMO2354	2.382 down
LMO2355	3.979 down
LMO2357	2.564 up
LMO2365	2.942 down
LMO2369	2.387 down
LMO2374	3.074 down
LMO2375	2.394 down
LMO2377	6.045 down
LMO2378	3.763 down
LMO2379	2.538 down
LMO2380	2.402 down
LMO2381	2.208 down
LMO2388	3.077 down
LMO2390	2.265 down
LMO2395	4.074 down
LMO2396	2.070 down
LMO2397	2.871 down
LMO2408	2.534 down
LMO2409	2.964 down
LMO2410	2.392 down
LMO2416	3.825 down
LMO2418	3.007 down
LMO2419	3.296 down
LMO2424	3.694 down

LMO2427	2.347 down
LMO2428	4.484 down
LMO2433	3.348 down
LMO2435	6.448 down
LMO2436	2.289 up
LMO2439	2.187 down
LMO2442	2.637 down
LMO2443	6.852 down
LMO2450	2.050 down
LMO2460	2.972 down
LMO2464	5.407 down
LMO2465	5.510 down
LMO2466	7.547 down
LMO2467	2.298 down
LMO2469	2.302 down
LMO2475	2.389 down
LMO2476	2.896 down
LMO2477	2.292 down
LMO2487	2.307 up
LMO2491	2.446 down
LMO2492	3.936 down
LMO2493	5.262 down
LMO2494	2.118 up
LMO2503	2.508 down
LMO2504	6.397 down
LMO2505	4.205 down
LMO2506	5.313 down
LMO2507	4.428 down
LMO2509	2.112 down
LMO2511	3.764 up
LMO2512	2.676 down
LMO2513	3.799 down
LMO2516	4.699 down
LMO2517	3.640 down
LMO2518	3.390 down
LMO2519	2.459 down
LMO2520	2.106 down
LMO2521	3.040 down
LMO2522	3.271 down
LMO2525	2.041 down
LMO2526	3.414 down
LMO2527	2.365 down
LMO2537	2.662 down
LMO2538	2.098 down
LMO2544	2.158 down
LMO2545	2.719 down
LMO2546	2.747 down
LMO2547	7.032 down

LMO2549	3.348 down
LMO2550	4.297 down
LMO2551	4.932 down
LMO2552	2.412 down
LMO2558	2.825 down
LMO2559	5.051 down
LMO2561	3.415 down
LMO2562	3.036 down
LMO2563	2.842 down
LMO2566	2.269 down
LMO2567	3.985 up
LMO2568	2.095 up
LMO2569	2.462 down
LMO2570	2.112 up
LMO2574	2.104 up
LMO2576	2.348 down
LMO2577	2.155 down
LMO2584	3.283 up
LMO2585	7.101 up
LMO2586	7.368 up
LMO2587	3.661 down
LMO2591	9.855 down
LMO2596	2.817 down
LMO2597	2.042 down
LMO2601	2.183 down
LMO2603	2.421 up
LMO2628	2.052 down
LMO2629	2.000 down
LMO2634	2.154 down
LMO2645	40.633 up
LMO2646	32.680 up
LMO2647	53.486 up
LMO2648	47.191 up
LMO2649	32.946 up
LMO2650	26.143 up
LMO2651	33.435 up
LMO2661	8.132 up
LMO2662	8.669 up
LMO2663	9.744 up
LMO2664	9.634 up
LMO2665	14.893 up
LMO2666	15.747 up
LMO2667	6.649 up
LMO2668	7.161 up
LMO2670	3.495 up
LMO2671	2.377 up
LMO2673	2.096 up
LMO2674	3.332 up

LMO2681	2.798 up
LMO2682	3.003 up
LMO2683	8.343 up
LMO2684	8.427 up
LMO2685	11.418 up
LMO2686	7.288 down
LMO2687	2.941 down
LMO2688	3.496 down
LMO2689	8.642 down
LMO2690	28.488 down
LMO2704	2.048 down
LMO2707	2.863 up
LMO2708	20.466 up
LMO2710	3.768 down
LMO2713	3.577 up
LMO2714	2.694 up
LMO2719	4.555 down
LMO2720	7.407 down
LMO2724	2.409 up
LMO2725	2.554 down
LMO2726	4.515 down
LMO2727	2.817 down
LMO2733	4.733 up
LMO2734	6.192 up
LMO2735	6.244 up
LMO2736	2.065 up
LMO2737	4.242 down
LMO2742	9.154 up
LMO2743	4.336 up
LMO2744	2.520 down
LMO2749	4.094 down
LMO2750	2.431 down
LMO2753	3.822 down
LMO2754	3.175 down
LMO2755	2.035 down
LMO2756	2.604 down
LMO2758	4.119 down
LMO2761	2.187 down
LMO2766	2.290 down
LMO2767	2.016 down
LMO2768	2.222 down
LMO2769	3.718 down
LMO2771	2.043 up
LMO2772	4.708 up
LMO2777	2.956 down
LMO2779	5.135 down
LMO2780	3.774 up
LMO2781	15.760 up

LMO2782	27.191	up
LMO2783	18.995	up
LMO2786	2.144	down
LMO2787	2.245	down
LMO2791	2.181	down
LMO2792	2.649	up
LMO2793	3.320	down
LMO2794	2.650	down
LMO2799	3.864	up
LMO2800	3.867	up
LMO2801	9.175	up
LMO2810	3.472	down
LMO2811	5.263	down
LMO2812	4.688	up
LMO2814	2.837	down
LMO2816	15.953	up
LMO2817	6.409	up
LMO2818	5.167	up
LMO2819	2.630	up
LMO2826	4.158	down
LMO2827	4.849	down
LMO2828	2.028	up
LMO2829	3.171	down
LMO2842	3.384	down
LMO2843	2.192	down
LMO2844	2.473	down
LMO2845	2.566	down
LMO2846	2.508	up
LMO2847	3.324	up
LMO2848	7.039	up
LMO2849	8.746	up
LMO2850	16.839	up
LMO2851	11.194	up
LMO2852	4.724	down
LMO2854	2.514	down
LMO2855	2.612	down
LMO2856	3.056	down
LMO2857	7.087	down
