# Supplementary material for: Changes in Gene Expression during Adaptation of Listeria monocytogenes to the Soil Environment
Source: PLoS One. 2011 Sep 23;6(9):e24881. doi: 10.1371/journal.pone.0024881 (PMC3179493; doi:10.1371/journal.pone.0024881)
Supplement: Table S2 — Genes over 2-fold change at time 30 minutes. (PDF) [file pone.0024881.s002.pdf]

Table S2. Genes over 2-fold change at time 30 minutes.

listilist ID	Fold change TA 30 vs T0
LMO0001	3.525 down
LMO0002	3.393 down
LMO0003	7.593 down
LMO0004	3.859 down
LMO0005	3.191 down
LMO0008	2.915 down
LMO0009	4.931 down
LMO0010	2.802 down
LMO0013	2.639 down
LMO0017	6.566 down
LMO0020	2.149 up
LMO0021	12.812 up
LMO0022	19.697 up
LMO0023	14.374 up
LMO0024	25.296 up
LMO0027	41.540 up
LMO0028	2.508 down
LMO0029	5.104 down
LMO0030	4.707 down
LMO0031	3.188 down
LMO0032	2.063 down
LMO0033	3.449 down
LMO0034	5.131 down
LMO0035	3.678 down
LMO0039	2.076 up
LMO0040	3.777 down
LMO0041	3.443 down
LMO0042	13.250 down
LMO0046	4.639 down
LMO0047	2.229 up
LMO0050	5.331 down
LMO0051	4.445 down
LMO0055	6.400 down
LMO0057	2.583 down
LMO0058	3.141 down
LMO0059	2.234 down
LMO0060	2.136 down
LMO0066	2.200 down
LMO0070	3.204 down
LMO0071	9.228 down
LMO0072	13.701 down
LMO0073	7.649 down
LMO0074	7.022 down
LMO0075	3.989 down

LMO0076	2.841 down
LMO0077	6.316 down
LMO0079	3.806 down
LMO0080	2.692 down
LMO0081	2.043 down
LMO0086	2.319 down
LMO0094	2.444 down
LMO0095	2.486 down
LMO0099	2.751 down
LMO0100	2.113 down
LMO0101	3.220 down
LMO0102	4.007 down
LMO0103	3.103 down
LMO0104	2.973 down
LMO0105	43.506 up
LMO0106	7.111 down
LMO0107	2.061 down
LMO0108	2.559 down
LMO0111	10.735 down
LMO0112	6.948 down
LMO0113	2.892 up
LMO0114	2.139 up
LMO0119	2.224 up
LMO0123	2.046 up
LMO0127	2.074 up
LMO0128	2.131 up
LMO0132	3.651 down
LMO0133	2.415 up
LMO0134	2.746 up
LMO0135	2.743 down
LMO0136	2.663 down
LMO0137	3.062 down
LMO0138	4.296 down
LMO0139	4.049 down
LMO0140	4.131 down
LMO0141	2.188 down
LMO0142	6.600 down
LMO0143	3.817 down
LMO0144	6.736 down
LMO0145	7.302 down
LMO0146	4.388 down
LMO0149	2.725 down
LMO0150	2.802 down
LMO0151	12.152 down
LMO0152	11.850 down
LMO0153	20.115 down
LMO0154	19.330 down
LMO0155	5.535 down

LMO0156	4.009 down
LMO0157	2.177 down
LMO0158	4.188 down
LMO0159	3.994 down
LMO0160	4.016 down
LMO0161	2.129 down
LMO0162	2.728 down
LMO0164	2.294 down
LMO0165	2.616 down
LMO0166	2.816 down
LMO0167	3.795 down
LMO0168	4.601 down
LMO0175	2.097 down
LMO0176	6.700 down
LMO0177	2.619 down
LMO0181	2.426 up
LMO0185	9.895 down
LMO0186	10.168 down
LMO0187	6.282 down
LMO0188	2.212 down
LMO0189	4.872 down
LMO0190	7.352 down
LMO0191	2.950 down
LMO0192	6.369 down
LMO0197	2.040 down
LMO0198	5.220 down
LMO0199	9.179 down
LMO0201	3.726 down
LMO0203	2.851 down
LMO0207	3.509 down
LMO0208	2.509 down
LMO0212	2.391 down
LMO0213	4.993 down
LMO0217	2.954 down
LMO0218	3.930 down
LMO0219	4.062 down
LMO0221	3.474 down
LMO0222	4.864 down
LMO0223	4.454 down
LMO0228	2.536 down
LMO0229	2.006 up
LMO0234	2.416 down
LMO0238	5.112 down
LMO0239	4.048 down
LMO0240	3.294 down
LMO0241	5.321 down
LMO0242	2.980 down
LMO0243	5.776 down

LMO0244	14.816	down
LMO0245	26.173	down
LMO0246	7.810	down
LMO0247	4.104	down
LMO0248	2.574	down
LMO0249	2.013	down
LMO0251	2.348	down
LMO0252	11.515	down
LMO0253	4.717	down
LMO0254	2.621	down
LMO0256	5.755	down
LMO0262	3.872	down
LMO0265	2.498	up
LMO0269	15.999	down
LMO0272	5.969	down
LMO0273	9.167	down
LMO0276	2.295	down
LMO0277	3.025	down
LMO0279	3.169	up
LMO0281	3.213	down
LMO0282	11.613	down
LMO0283	13.149	down
LMO0284	16.512	down
LMO0285	18.870	down
LMO0286	17.356	down
LMO0287	4.177	down
LMO0288	3.860	down
LMO0289	3.111	down
LMO0290	2.239	down
LMO0291	2.470	down
LMO0293	8.006	down
LMO0294	9.248	down
LMO0295	3.524	down
LMO0296	6.260	down
LMO0297	2.584	up
LMO0298	15.822	up
LMO0299	17.041	up
LMO0300	13.810	up
LMO0301	12.663	up
LMO0302	5.851	down
LMO0303	3.588	down
LMO0304	5.619	down
LMO0306	4.839	down
LMO0307	2.561	down
LMO0312	3.072	down
LMO0313	4.087	down
LMO0314	4.013	down
LMO0315	4.760	down

LMO0316	3.766	down
LMO0317	2.345	down
LMO0319	3.403	up
LMO0320	5.118	down
LMO0321	5.375	down
LMO0322	5.685	down
LMO0323	2.845	up
LMO0326	4.315	down
LMO0327	2.847	down
LMO0331	2.979	down
LMO0332	3.602	down
LMO0333	7.408	down
LMO0335	2.054	down
LMO0336	2.383	down
LMO0337	4.957	down
LMO0338	5.336	down
LMO0342	2.620	up
LMO0343	4.285	up
LMO0344	6.249	up
LMO0345	5.345	up
LMO0346	8.345	up
LMO0347	5.598	up
LMO0348	6.555	up
LMO0349	4.710	up
LMO0350	3.698	up
LMO0351	2.768	up
LMO0354	2.836	down
LMO0356	2.478	down
LMO0358	2.452	up
LMO0360	3.620	down
LMO0361	3.586	down
LMO0362	2.625	down
LMO0364	14.476	down
LMO0365	9.607	down
LMO0366	4.056	down
LMO0367	3.366	down
LMO0368	2.430	down
LMO0370	2.291	down
LMO0371	13.792	down
LMO0372	2.239	down
LMO0374	2.529	down
LMO0380	2.721	down
LMO0381	2.767	down
LMO0382	4.910	down
LMO0383	8.293	up
LMO0384	9.531	up
LMO0385	5.751	up
LMO0386	5.058	up

LMO0387	4.386 down
LMO0388	3.989 down
LMO0389	4.059 down
LMO0390	8.836 down
LMO0394	29.478 down
LMO0395	9.357 down
LMO0396	4.312 down
LMO0397	2.521 down
LMO0398	3.361 up
LMO0399	3.478 up
LMO0400	7.580 up
LMO0401	20.009 up
LMO0403	6.757 down
LMO0404	4.730 down
LMO0409	2.673 down
LMO0410	3.500 down
LMO0411	2.497 down
LMO0412	4.913 down
LMO0413	2.463 down
LMO0414	2.522 down
LMO0416	2.710 down
LMO0417	2.304 down
LMO0419	3.144 down
LMO0420	3.359 down
LMO0430	12.412 down
LMO0435	3.837 down
LMO0436	8.521 down
LMO0437	2.139 down
LMO0438	2.503 down
LMO0440	7.113 down
LMO0441	2.095 down
LMO0442	4.153 down
LMO0448	3.604 down
LMO0449	12.737 down
LMO0450	6.800 down
LMO0451	5.142 down
LMO0452	3.710 down
LMO0453	4.889 down
LMO0454	7.373 down
LMO0455	6.697 down
LMO0459	3.951 down
LMO0460	3.028 down
LMO0465	8.293 down
LMO0467	2.275 down
LMO0468	4.774 down
LMO0469	28.258 down
LMO0470	10.380 down
LMO0471	2.147 up

LMO0472	5.167 down
LMO0473	5.133 down
LMO0474	5.472 down
LMO0475	7.106 down
LMO0476	3.888 down
LMO0477	17.337 down
LMO0478	17.348 down
LMO0479	7.051 down
LMO0480	3.323 down
LMO0482	6.916 down
LMO0484	2.790 down
LMO0485	20.141 down
LMO0486	10.802 down
LMO0487	3.582 down
LMO0488	2.050 down
LMO0489	3.127 down
LMO0491	2.904 down
LMO0492	5.213 down
LMO0493	2.774 down
LMO0494	4.636 down
LMO0495	7.569 down
LMO0497	10.180 down
LMO0501	3.259 up
LMO0502	6.171 up
LMO0503	4.361 up
LMO0504	3.775 up
LMO0505	2.785 up
LMO0506	2.421 down
LMO0507	2.487 up
LMO0509	9.177 down
LMO0510	8.151 down
LMO0511	8.709 down
LMO0512	2.374 down
LMO0513	4.780 down
LMO0514	27.493 down
LMO0515	2.850 up
LMO0517	6.903 up
LMO0518	4.462 down
LMO0519	10.472 down
LMO0520	3.913 down
LMO0522	5.366 down
LMO0523	4.073 down
LMO0525	4.221 down
LMO0526	2.920 down
LMO0527	3.022 down
LMO0532	3.507 down
LMO0533	3.230 down
LMO0535	2.284 down

LMO0536	5.857 up
LMO0537	2.941 down
LMO0541	6.902 down
LMO0544	7.390 up
LMO0545	5.452 up
LMO0546	2.483 up
LMO0547	4.427 down
LMO0548	3.466 down
LMO0549	6.219 down
LMO0550	5.083 down
LMO0551	3.303 down
LMO0552	4.104 down
LMO0557	2.128 down
LMO0558	2.338 down
LMO0559	14.544 down
LMO0560	3.157 down
LMO0569	2.376 down
LMO0570	4.355 down
LMO0571	4.035 down
LMO0572	2.713 down
LMO0573	4.658 down
LMO0575	2.824 down
LMO0576	2.344 down
LMO0577	2.390 down
LMO0578	2.815 down
LMO0581	8.774 down
LMO0582	6.776 down
LMO0583	3.523 down
LMO0584	3.325 up
LMO0585	8.303 down
LMO0586	10.479 down
LMO0587	3.888 down
LMO0588	9.377 down
LMO0589	4.219 down
LMO0590	2.498 down
LMO0593	2.381 down
LMO0594	2.581 down
LMO0595	3.127 down
LMO0597	9.881 down
LMO0598	8.163 down
LMO0599	5.619 down
LMO0600	4.625 down
LMO0601	4.205 down
LMO0603	4.351 down
LMO0604	7.895 down
LMO0605	4.232 down
LMO0606	2.065 down
LMO0608	2.181 up

LMO0609	10.771 down
LMO0611	13.080 down
LMO0614	3.022 down
LMO0615	4.459 down
LMO0616	7.003 down
LMO0617	2.135 down
LMO0618	4.869 down
LMO0619	2.841 down
LMO0620	3.046 down
LMO0621	3.459 down
LMO0622	3.582 down
LMO0623	2.015 down
LMO0625	2.123 up
LMO0626	2.828 down
LMO0627	2.335 down
LMO0628	2.684 up
LMO0629	2.377 up
LMO0630	4.485 up
LMO0631	2.922 up
LMO0632	4.672 up
LMO0633	4.049 up
LMO0634	3.930 up
LMO0635	8.328 down
LMO0636	4.745 down
LMO0637	4.437 down
LMO0638	13.910 down
LMO0642	4.082 down
LMO0643	4.796 up
LMO0644	4.630 down
LMO0645	6.208 down
LMO0646	2.792 down
LMO0648	3.174 down
LMO0652	2.622 down
LMO0654	4.301 down
LMO0655	8.055 down
LMO0656	24.238 down
LMO0657	5.238 down
LMO0658	4.995 down
LMO0659	2.520 down
LMO0662	2.115 down
LMO0663	2.445 down
LMO0664	6.738 down
LMO0665	5.657 down
LMO0666	5.934 down
LMO0667	4.319 down
LMO0668	5.774 down
LMO0671	3.106 down
LMO0672	10.709 down

LMO0677	2.284	down
LMO0678	10.497	down
LMO0679	13.307	down
LMO0680	12.536	down
LMO0681	7.433	down
LMO0682	5.960	down
LMO0683	23.620	down
LMO0684	11.504	down
LMO0685	3.360	down
LMO0686	2.073	down
LMO0691	5.016	down
LMO0692	4.249	down
LMO0693	6.426	down
LMO0694	4.623	down
LMO0695	3.085	down
LMO0696	3.822	down
LMO0701	2.132	down
LMO0703	2.967	down
LMO0704	2.559	down
LMO0705	2.065	down
LMO0721	7.791	down
LMO0725	22.143	down
LMO0726	10.453	down
LMO0727	6.911	down
LMO0728	12.177	down
LMO0729	2.180	down
LMO0730	2.133	down
LMO0731	3.192	down
LMO0732	2.642	down
LMO0733	5.619	down
LMO0734	2.925	down
LMO0739	4.130	down
LMO0740	7.475	down
LMO0742	2.779	down
LMO0744	2.401	down
LMO0745	4.567	down
LMO0748	4.735	down
LMO0749	3.199	down
LMO0750	3.190	down
LMO0751	3.255	down
LMO0752	3.512	down
LMO0753	2.429	down
LMO0755	2.180	down
LMO0756	2.851	down
LMO0757	3.235	down
LMO0763	2.713	down
LMO0764	3.941	down
LMO0765	8.739	down

LMO0766	3.357	down
LMO0767	3.902	down
LMO0768	2.649	down
LMO0769	2.989	down
LMO0770	6.499	down
LMO0771	19.239	down
LMO0772	13.413	down
LMO0773	3.463	down
LMO0774	2.374	down
LMO0775	2.533	down
LMO0776	2.952	down
LMO0777	9.301	down
LMO0778	10.710	down
LMO0779	7.695	down
LMO0781	2.357	up
LMO0785	5.601	down
LMO0786	2.288	down
LMO0787	4.482	down
LMO0788	3.816	down
LMO0789	4.042	down
LMO0790	10.621	down
LMO0791	7.977	down
LMO0792	3.096	down
LMO0793	6.677	down
LMO0795	6.420	down
LMO0797	3.523	down
LMO0798	2.669	down
LMO0799	2.073	down
LMO0801	4.341	down
LMO0802	17.176	down
LMO0803	3.949	down
LMO0804	4.739	down
LMO0805	3.494	down
LMO0806	9.330	down
LMO0807	4.463	down
LMO0808	2.630	down
LMO0811	3.993	down
LMO0812	4.479	down
LMO0814	6.380	down
LMO0815	5.765	down
LMO0816	7.277	down
LMO0817	13.654	down
LMO0818	3.481	down
LMO0820	2.967	down
LMO0821	2.889	down
LMO0824	2.492	down
LMO0825	2.087	down
LMO0826	2.739	down

LMO0831	5.756 down
LMO0833	6.374 down
LMO0834	13.594 down
LMO0835	8.668 down
LMO0836	17.145 down
LMO0837	11.183 down
LMO0838	2.177 down
LMO0839	4.507 down
LMO0840	9.662 down
LMO0841	3.246 down
LMO0842	10.844 down
LMO0843	2.320 down
LMO0844	5.306 down
LMO0845	4.030 down
LMO0846	4.779 down
LMO0847	9.073 down
LMO0848	10.680 down
LMO0849	2.464 down
LMO0850	12.029 down
LMO0851	9.123 down
LMO0852	6.876 down
LMO0853	4.626 down
LMO0854	5.263 down
LMO0855	2.878 down
LMO0856	3.085 down
LMO0857	5.089 down
LMO0864	2.744 down
LMO0866	4.901 down
LMO0867	8.333 down
LMO0868	4.132 down
LMO0869	2.975 up
LMO0873	3.350 up
LMO0874	6.369 up
LMO0875	3.987 up
LMO0876	3.381 up
LMO0877	3.630 up
LMO0878	5.696 up
LMO0879	3.847 up
LMO0882	6.612 down
LMO0883	5.096 down
LMO0884	3.944 down
LMO0885	2.696 down
LMO0886	2.160 down
LMO0887	4.605 down
LMO0888	7.081 down
LMO0889	3.379 down
LMO0890	3.632 down
LMO0891	3.657 down

LMO0892	2.319 down
LMO0898	2.370 down
LMO0899	8.706 down
LMO0900	4.446 down
LMO0901	3.590 down
LMO0902	3.689 down
LMO0903	6.355 down
LMO0904	2.652 down
LMO0905	3.137 down
LMO0908	3.923 down
LMO0911	2.099 down
LMO0912	4.446 down
LMO0914	21.601 up
LMO0915	25.705 up
LMO0916	49.999 up
LMO0917	103.336 up
LMO0918	7.014 up
LMO0920	6.428 down
LMO0921	4.673 down
LMO0922	5.482 down
LMO0923	2.141 down
LMO0924	2.129 down
LMO0926	2.491 down
LMO0927	4.322 down
LMO0928	6.154 down
LMO0932	2.077 down
LMO0933	2.274 down
LMO0934	2.902 down
LMO0935	3.094 down
LMO0937	4.352 down
LMO0938	3.477 down
LMO0939	10.575 down
LMO0942	2.215 down
LMO0944	2.041 down
LMO0948	3.550 down
LMO0949	3.012 down
LMO0950	3.130 down
LMO0951	4.324 down
LMO0952	13.482 down
LMO0954	6.450 up
LMO0955	5.585 up
LMO0956	2.041 up
LMO0957	2.653 up
LMO0959	7.703 down
LMO0960	2.952 down
LMO0961	2.771 down
LMO0965	2.178 down
LMO0966	4.454 down

LMO0967	3.767 down
LMO0968	4.252 down
LMO0969	3.382 down
LMO0970	4.528 down
LMO0971	2.748 down
LMO0972	2.981 down
LMO0973	3.795 down
LMO0974	3.460 down
LMO0975	4.200 down
LMO0976	2.570 down
LMO0978	2.889 down
LMO0981	2.843 down
LMO0984	19.097 up
LMO0985	13.917 up
LMO0986	13.445 up
LMO0987	2.644 up
LMO0988	3.703 down
LMO0989	2.476 down
LMO0990	2.930 down
LMO0995	5.050 down
LMO0997	5.672 up
LMO0998	39.280 down
LMO0999	9.405 down
LMO1000	6.617 down
LMO1001	11.536 down
LMO1004	8.884 down
LMO1005	2.958 down
LMO1006	4.978 down
LMO1008	8.857 down
LMO1009	4.832 down
LMO1010	2.912 down
LMO1011	4.556 down
LMO1012	3.724 down
LMO1013	5.193 down
LMO1014	7.069 down
LMO1015	4.972 down
LMO1016	4.867 down
LMO1017	7.283 down
LMO1024	7.343 down
LMO1025	7.350 down
LMO1026	3.631 down
LMO1027	2.721 down
LMO1028	3.751 down
LMO1030	10.841 down
LMO1033	2.043 down
LMO1037	7.708 down
LMO1038	2.862 down
LMO1039	2.965 down

LMO1040	2.859 down
LMO1041	2.466 down
LMO1043	2.202 up
LMO1044	2.071 up
LMO1045	2.269 up
LMO1046	3.115 up
LMO1047	2.300 up
LMO1048	2.167 up
LMO1050	2.821 down
LMO1056	6.295 down
LMO1060	4.162 down
LMO1061	3.266 down
LMO1063	2.177 down
LMO1064	7.592 down
LMO1065	4.957 down
LMO1066	4.673 down
LMO1067	9.347 down
LMO1068	2.939 up
LMO1069	4.513 down
LMO1070	4.727 down
LMO1071	9.044 down
LMO1072	2.600 down
LMO1073	12.311 down
LMO1074	8.804 down
LMO1075	4.113 down
LMO1076	5.003 down
LMO1077	8.226 down
LMO1078	16.484 down
LMO1079	10.173 down
LMO1080	4.039 down
LMO1081	2.044 down
LMO1082	2.239 down
LMO1085	4.208 down
LMO1086	4.581 down
LMO1087	2.986 down
LMO1088	4.723 down
LMO1089	5.129 down
LMO1090	5.196 down
LMO1091	4.570 down
LMO1092	3.029 down
LMO1093	2.423 down
LMO1096	4.132 down
LMO1100	2.685 down
LMO1102	2.418 down
LMO1113	2.096 up
LMO1114	2.070 up
LMO1116	3.021 down
LMO1117	4.111 down

LMO1118	8.544 down
LMO1119	9.804 down
LMO1120	9.740 down
LMO1121	3.234 down
LMO1122	5.159 down
LMO1123	6.601 down
LMO1124	4.058 down
LMO1125	2.757 down
LMO1126	2.894 down
LMO1127	4.128 down
LMO1128	5.599 down
LMO1129	4.215 down
LMO1130	6.079 down
LMO1131	6.517 down
LMO1132	3.461 down
LMO1134	7.480 down
LMO1135	7.990 down
LMO1136	8.920 down
LMO1137	2.578 up
LMO1140	2.322 up
LMO1141	7.581 down
LMO1144	3.832 down
LMO1145	3.660 down
LMO1146	3.376 down
LMO1147	4.381 down
LMO1148	4.553 down
LMO1149	7.577 down
LMO1150	2.085 down
LMO1169	4.561 down
LMO1170	5.479 down
LMO1172	2.922 down
LMO1175	4.199 down
LMO1176	2.597 down
LMO1177	2.819 down
LMO1178	2.530 down
LMO1179	2.967 down
LMO1180	3.865 down
LMO1181	5.754 down
LMO1182	4.047 down
LMO1183	5.244 down
LMO1184	3.747 down
LMO1185	4.901 down
LMO1186	4.025 down
LMO1188	4.688 down
LMO1190	3.535 down
LMO1191	2.370 down
LMO1193	2.296 down
LMO1194	2.264 down

LMO1199	2.565 down
LMO1200	2.837 down
LMO1201	5.110 down
LMO1202	5.355 down
LMO1203	4.244 down
LMO1204	4.241 down
LMO1205	3.807 down
LMO1206	3.449 down
LMO1207	3.682 down
LMO1208	4.429 down
LMO1209	2.435 down
LMO1210	11.844 down
LMO1211	10.100 down
LMO1212	2.222 down
LMO1213	4.194 down
LMO1214	4.406 down
LMO1215	2.151 down
LMO1216	3.713 down
LMO1219	2.108 down
LMO1220	2.069 down
LMO1221	3.208 down
LMO1222	2.852 down
LMO1223	11.244 down
LMO1224	3.388 down
LMO1225	5.808 down
LMO1227	2.461 down
LMO1228	5.209 down
LMO1229	7.453 down
LMO1230	5.275 down
LMO1231	2.746 down
LMO1232	2.818 down
LMO1235	7.577 down
LMO1236	10.095 down
LMO1237	16.010 down
LMO1238	8.515 down
LMO1239	15.873 down
LMO1240	9.622 down
LMO1241	2.147 up
LMO1242	3.015 down
LMO1243	3.594 down
LMO1244	2.008 down
LMO1245	6.260 down
LMO1246	7.166 down
LMO1247	11.565 down
LMO1248	31.331 down
LMO1249	12.146 down
LMO1250	4.324 down
LMO1251	31.537 down

LMO1252	7.784 down
LMO1253	5.668 down
LMO1257	2.028 down
LMO1259	2.140 down
LMO1260	4.758 down
LMO1262	3.694 down
LMO1263	2.023 down
LMO1266	5.815 down
LMO1267	2.615 down
LMO1268	2.918 down
LMO1269	2.384 down
LMO1270	2.603 down
LMO1271	3.543 down
LMO1272	3.175 down
LMO1273	3.737 down
LMO1274	3.351 down
LMO1275	11.284 down
LMO1276	5.246 down
LMO1280	2.390 down
LMO1282	2.407 down
LMO1283	2.465 down
LMO1284	7.146 down
LMO1285	3.602 down
LMO1286	3.781 down
LMO1287	2.050 down
LMO1288	2.191 down
LMO1291	4.413 down
LMO1292	12.758 down
LMO1293	2.850 up
LMO1294	6.565 down
LMO1295	5.854 down
LMO1296	6.448 down
LMO1297	5.424 down
LMO1298	21.531 down
LMO1299	2.095 down
LMO1300	8.938 down
LMO1305	2.671 down
LMO1306	7.733 down
LMO1307	14.260 down
LMO1308	5.605 down
LMO1309	6.806 down
LMO1310	17.213 down
LMO1311	18.795 down
LMO1312	13.107 down
LMO1313	5.040 down
LMO1314	4.313 down
LMO1315	7.156 down
LMO1316	7.050 down

LMO1317	3.747	down
LMO1318	2.334	down
LMO1319	2.448	down
LMO1321	2.923	down
LMO1322	2.918	down
LMO1323	2.338	down
LMO1326	3.385	down
LMO1327	6.042	down
LMO1328	7.646	down
LMO1329	4.404	down
LMO1330	7.381	down
LMO1331	12.179	down
LMO1333	5.300	down
LMO1334	3.697	down
LMO1335	7.231	down
LMO1336	3.984	down
LMO1337	4.049	down
LMO1338	2.943	down
LMO1339	3.282	down
LMO1341	2.689	down
LMO1347	4.999	down
LMO1348	2.629	up
LMO1349	9.321	up
LMO1350	4.750	up
LMO1351	5.355	down
LMO1352	4.859	down
LMO1353	21.983	down
LMO1354	2.241	down
LMO1355	3.528	down
LMO1356	5.517	down
LMO1357	3.185	down
LMO1358	2.904	down
LMO1359	4.409	down
LMO1360	3.042	down
LMO1361	2.119	down
LMO1362	2.308	down
LMO1363	2.357	down
LMO1364	4.838	down
LMO1365	6.775	down
LMO1366	5.309	down
LMO1368	2.531	down
LMO1369	9.032	down
LMO1370	9.082	down
LMO1371	2.744	down
LMO1375	3.764	down
LMO1376	2.435	down
LMO1377	2.560	down
LMO1378	2.812	down

LMO1384	10.912	down
LMO1385	5.866	down
LMO1387	2.816	down
LMO1392	3.865	down
LMO1393	2.052	down
LMO1394	2.969	down
LMO1395	18.656	down
LMO1396	14.311	down
LMO1397	2.459	down
LMO1398	2.438	down
LMO1399	2.334	down
LMO1400	5.048	down
LMO1401	5.307	down
LMO1402	4.198	down
LMO1403	3.112	down
LMO1406	2.182	down
LMO1407	2.063	down
LMO1408	2.629	down
LMO1409	10.580	down
LMO1410	7.698	down
LMO1411	5.543	down
LMO1412	4.439	down
LMO1414	3.739	down
LMO1415	2.962	down
LMO1416	13.797	down
LMO1417	20.865	down
LMO1418	12.578	down
LMO1419	15.115	down
LMO1420	11.929	down
LMO1421	2.603	down
LMO1424	3.639	down
LMO1427	2.147	down
LMO1428	9.492	down
LMO1429	37.743	down
LMO1430	6.910	down
LMO1431	15.737	down
LMO1436	3.700	down
LMO1437	6.454	down
LMO1438	2.908	down
LMO1440	38.745	down
LMO1441	4.680	down
LMO1442	5.376	down
LMO1443	8.008	down
LMO1444	2.166	down
LMO1445	15.151	down
LMO1446	18.618	down
LMO1447	13.432	down
LMO1448	2.518	down

LMO1449	4.310	down
LMO1450	4.462	down
LMO1451	2.993	down
LMO1453	3.552	down
LMO1457	2.473	down
LMO1458	2.567	down
LMO1459	2.779	down
LMO1461	2.385	down
LMO1462	3.243	down
LMO1463	4.083	down
LMO1464	5.192	down
LMO1465	3.121	down
LMO1466	3.048	down
LMO1467	3.166	down
LMO1468	4.430	down
LMO1469	2.628	down
LMO1476	5.053	down
LMO1478	2.976	down
LMO1479	4.485	down
LMO1480	7.628	down
LMO1481	4.036	down
LMO1482	2.684	down
LMO1483	7.485	down
LMO1484	2.709	down
LMO1485	20.037	down
LMO1486	6.991	down
LMO1487	4.819	down
LMO1488	4.162	down
LMO1489	4.118	down
LMO1490	4.651	down
LMO1491	5.743	down
LMO1492	14.545	down
LMO1494	5.233	down
LMO1495	3.522	down
LMO1496	6.567	down
LMO1497	4.318	down
LMO1498	8.164	down
LMO1499	9.171	down
LMO1500	6.339	down
LMO1504	3.462	down
LMO1505	2.471	down
LMO1506	5.211	down
LMO1507	2.303	down
LMO1509	2.105	down
LMO1510	2.833	down
LMO1511	3.682	down
LMO1512	4.095	down
LMO1513	4.575	down

LMO1514	2.112 down
LMO1515	21.982 down
LMO1516	2.980 down
LMO1517	3.125 down
LMO1518	6.621 down
LMO1519	3.575 down
LMO1520	4.063 down
LMO1521	5.069 down
LMO1522	6.238 down
LMO1523	3.883 down
LMO1524	6.199 down
LMO1525	2.474 down
LMO1528	4.402 down
LMO1529	3.194 down
LMO1530	4.297 down
LMO1531	2.631 down
LMO1532	2.683 down
LMO1533	2.342 down
LMO1534	2.411 down
LMO1535	2.297 down
LMO1536	6.391 down
LMO1537	6.460 down
LMO1538	3.258 up
LMO1540	2.918 down
LMO1543	2.895 down
LMO1544	3.887 down
LMO1545	6.947 down
LMO1546	5.764 down
LMO1547	3.379 down
LMO1548	3.205 down
LMO1549	9.079 down
LMO1550	3.613 down
LMO1551	6.060 down
LMO1552	3.253 down
LMO1554	2.398 down
LMO1555	6.197 down
LMO1556	6.863 down
LMO1557	8.315 down
LMO1558	16.634 down
LMO1559	3.150 down
LMO1560	2.119 down
LMO1565	2.401 down
LMO1566	2.180 down
LMO1568	5.657 down
LMO1569	4.704 down
LMO1571	2.686 down
LMO1572	2.843 down
LMO1573	2.291 down

LMO1574	3.327 down
LMO1575	3.118 down
LMO1576	3.248 down
LMO1577	3.149 down
LMO1580	2.612 up
LMO1581	2.987 down
LMO1582	3.908 down
LMO1583	3.148 down
LMO1584	7.020 down
LMO1585	10.777 down
LMO1587	2.258 up
LMO1588	3.658 up
LMO1589	4.836 up
LMO1590	7.591 up
LMO1591	4.334 up
LMO1592	3.810 down
LMO1593	3.381 down
LMO1594	4.263 down
LMO1595	3.037 down
LMO1596	3.528 down
LMO1598	2.834 down
LMO1599	2.463 down
LMO1600	5.691 down
LMO1603	4.369 down
LMO1604	6.898 down
LMO1606	2.595 down
LMO1613	3.810 down
LMO1614	12.710 down
LMO1615	10.204 down
LMO1616	11.631 down
LMO1617	5.451 down
LMO1618	8.097 down
LMO1621	2.639 down
LMO1622	5.877 down
LMO1623	8.784 down
LMO1624	4.973 down
LMO1625	8.249 down
LMO1626	6.647 down
LMO1627	2.199 down
LMO1635	13.653 down
LMO1636	2.843 down
LMO1639	4.130 down
LMO1640	4.545 down
LMO1641	3.348 down
LMO1642	2.977 down
LMO1643	2.457 down
LMO1644	2.109 down
LMO1645	4.249 down

LMO1646	8.279 down
LMO1647	11.142 down
LMO1648	7.935 down
LMO1653	6.102 down
LMO1654	7.406 down
LMO1655	4.221 down
LMO1656	10.562 down
LMO1657	3.245 down
LMO1658	2.386 down
LMO1659	2.783 down
LMO1660	4.221 down
LMO1661	2.520 down
LMO1662	4.363 down
LMO1663	5.549 down
LMO1664	9.570 down
LMO1665	15.417 down
LMO1667	2.901 down
LMO1668	3.654 down
LMO1669	9.027 down
LMO1670	15.116 down
LMO1671	11.570 down
LMO1672	2.001 down
LMO1673	2.427 down
LMO1675	2.463 down
LMO1676	4.099 down
LMO1677	6.983 down
LMO1678	2.463 down
LMO1680	2.748 down
LMO1681	3.446 down
LMO1682	6.289 down
LMO1685	2.317 down
LMO1686	4.137 down
LMO1687	6.146 down
LMO1688	4.931 down
LMO1689	11.745 down
LMO1691	4.031 down
LMO1692	5.602 down
LMO1693	3.267 down
LMO1695	3.214 down
LMO1696	22.171 down
LMO1697	4.600 down
LMO1698	8.723 down
LMO1699	3.135 down
LMO1700	4.540 down
LMO1701	2.216 down
LMO1702	2.075 down
LMO1705	2.122 down
LMO1706	3.712 down

LMO1707	5.068 down
LMO1708	3.817 down
LMO1709	3.818 down
LMO1710	8.692 down
LMO1711	2.974 down
LMO1712	2.128 down
LMO1713	5.661 down
LMO1714	2.256 down
LMO1715	4.254 down
LMO1716	2.895 down
LMO1717	3.763 down
LMO1718	4.214 up
LMO1719	4.219 up
LMO1720	3.472 up
LMO1721	3.346 down
LMO1722	6.664 down
LMO1723	7.139 down
LMO1724	8.584 down
LMO1725	8.656 down
LMO1728	2.358 up
LMO1729	2.971 up
LMO1730	12.135 up
LMO1731	6.124 up
LMO1732	4.314 up
LMO1734	2.095 down
LMO1735	6.345 down
LMO1736	5.902 down
LMO1737	5.415 down
LMO1738	22.157 down
LMO1739	19.969 down
LMO1740	23.491 down
LMO1742	3.467 down
LMO1743	4.083 down
LMO1744	6.070 down
LMO1745	2.933 down
LMO1746	3.108 down
LMO1748	19.872 down
LMO1749	17.608 down
LMO1750	7.091 down
LMO1751	2.335 down
LMO1752	2.306 down
LMO1753	5.634 down
LMO1755	2.020 down
LMO1756	2.093 down
LMO1759	2.023 down
LMO1760	3.957 down
LMO1761	9.954 down
LMO1762	9.027 down

LMO1763	4.203 down
LMO1764	2.274 up
LMO1770	2.322 down
LMO1775	4.224 down
LMO1776	11.745 down
LMO1777	4.600 down
LMO1778	6.631 down
LMO1779	22.031 down
LMO1781	2.941 down
LMO1783	3.073 down
LMO1784	2.132 down
LMO1787	4.541 down
LMO1791	2.536 up
LMO1796	4.165 down
LMO1797	3.752 down
LMO1800	2.550 down
LMO1801	2.465 down
LMO1802	4.411 down
LMO1803	2.770 down
LMO1804	8.247 down
LMO1805	9.709 down
LMO1806	3.573 down
LMO1807	3.526 down
LMO1808	3.064 down
LMO1809	12.013 down
LMO1810	12.924 down
LMO1811	5.570 down
LMO1812	5.601 down
LMO1813	3.894 down
LMO1814	3.443 down
LMO1815	8.195 down
LMO1816	15.166 down
LMO1817	4.949 down
LMO1818	2.186 down
LMO1825	2.496 down
LMO1826	5.561 down
LMO1827	4.516 down
LMO1828	4.432 down
LMO1829	3.097 down
LMO1831	2.226 up
LMO1837	2.444 down
LMO1838	3.108 down
LMO1839	3.396 down
LMO1840	9.346 down
LMO1841	20.478 down
LMO1842	7.893 down
LMO1843	2.468 down
LMO1844	4.470 down

LMO1845	2.008 down
LMO1847	2.910 down
LMO1848	3.861 down
LMO1849	3.436 down
LMO1850	4.968 down
LMO1851	3.840 down
LMO1854	2.901 down
LMO1864	16.639 down
LMO1865	12.083 down
LMO1866	5.878 down
LMO1868	2.091 down
LMO1869	5.842 down
LMO1870	49.645 down
LMO1871	6.753 down
LMO1872	6.960 down
LMO1873	3.235 down
LMO1874	2.733 down
LMO1875	4.136 down
LMO1878	9.619 down
LMO1880	7.823 down
LMO1881	3.835 down
LMO1882	2.956 down
LMO1883	6.114 up
LMO1884	5.185 down
LMO1885	6.991 down
LMO1886	3.293 down
LMO1887	3.476 down
LMO1889	5.668 down
LMO1890	6.069 down
LMO1891	6.513 down
LMO1892	4.218 down
LMO1893	2.209 down
LMO1899	2.003 down
LMO1900	3.354 down
LMO1901	2.448 down
LMO1902	4.919 down
LMO1903	20.373 down
LMO1904	2.364 down
LMO1905	2.848 down
LMO1906	2.534 down
LMO1907	2.923 down
LMO1908	3.984 down
LMO1909	5.257 down
LMO1910	6.180 down
LMO1911	44.636 down
LMO1915	6.132 down
LMO1916	2.919 down
LMO1918	7.093 down

LMO1919	5.457 down
LMO1920	5.008 down
LMO1921	2.954 down
LMO1923	3.063 down
LMO1924	2.338 down
LMO1925	6.769 down
LMO1926	5.654 down
LMO1927	3.943 down
LMO1928	4.055 down
LMO1929	2.375 down
LMO1930	2.822 down
LMO1931	2.693 down
LMO1932	6.027 down
LMO1933	6.817 down
LMO1934	2.163 down
LMO1935	2.522 down
LMO1936	4.348 down
LMO1937	8.082 down
LMO1939	3.135 down
LMO1940	4.364 down
LMO1941	2.107 down
LMO1944	9.166 down
LMO1946	4.491 down
LMO1947	3.251 down
LMO1948	3.033 down
LMO1949	3.132 down
LMO1950	3.624 down
LMO1951	3.759 down
LMO1952	3.869 down
LMO1956	5.017 down
LMO1957	9.501 down
LMO1958	9.462 down
LMO1959	8.174 down
LMO1960	4.664 down
LMO1961	3.202 down
LMO1965	3.759 down
LMO1966	4.594 up
LMO1967	3.799 up
LMO1971	2.266 down
LMO1976	3.902 down
LMO1977	10.951 down
LMO1978	6.659 down
LMO1979	5.810 down
LMO1980	5.336 down
LMO1981	7.534 down
LMO1982	8.939 down
LMO1984	3.381 up
LMO1985	3.287 up

LMO1986	3.737	up
LMO1987	3.654	up
LMO1988	2.954	up
LMO1989	2.889	up
LMO1990	2.656	up
LMO1991	2.947	up
LMO1994	3.328	down
LMO1996	2.735	down
LMO1997	6.082	up
LMO1998	6.294	up
LMO1999	10.879	up
LMO2000	13.563	up
LMO2001	7.748	up
LMO2002	7.982	up
LMO2003	2.620	up
LMO2004	2.165	up
LMO2009	3.261	down
LMO2010	3.578	down
LMO2011	5.375	down
LMO2012	4.581	down
LMO2015	2.611	down
LMO2016	2.181	down
LMO2017	2.105	down
LMO2018	3.352	down
LMO2019	2.693	down
LMO2020	5.624	down
LMO2021	9.202	down
LMO2022	2.556	down
LMO2023	5.786	down
LMO2028	2.375	down
LMO2029	5.300	down
LMO2030	2.961	down
LMO2031	2.753	down
LMO2033	2.992	down
LMO2034	5.044	down
LMO2035	4.352	down
LMO2036	2.518	down
LMO2037	3.590	down
LMO2038	5.402	down
LMO2039	3.620	down
LMO2040	8.151	down
LMO2041	13.437	down
LMO2042	22.966	down
LMO2044	7.555	down
LMO2045	21.067	down
LMO2046	18.552	down
LMO2047	5.099	down
LMO2048	11.684	down

LMO2049	2.809 down
LMO2050	2.975 up
LMO2051	3.752 down
LMO2052	9.676 down
LMO2053	10.209 down
LMO2054	3.465 down
LMO2055	2.990 down
LMO2056	5.239 down
LMO2057	3.546 down
LMO2058	7.185 down
LMO2059	6.698 down
LMO2060	4.830 down
LMO2061	7.823 down
LMO2062	18.586 down
LMO2063	8.257 down
LMO2064	3.342 down
LMO2070	5.782 down
LMO2071	4.021 down
LMO2072	4.706 down
LMO2073	4.802 down
LMO2074	4.134 down
LMO2075	3.040 down
LMO2076	3.438 down
LMO2077	4.028 down
LMO2078	6.018 down
LMO2079	7.673 down
LMO2080	6.805 down
LMO2081	4.529 down
LMO2082	4.484 down
LMO2086	9.196 down
LMO2087	2.865 down
LMO2090	4.368 up
LMO2091	5.981 up
LMO2092	2.928 down
LMO2094	3.125 down
LMO2095	3.218 down
LMO2096	4.592 down
LMO2100	12.746 down
LMO2103	3.210 down
LMO2104	11.771 down
LMO2105	14.593 down
LMO2106	9.373 down
LMO2107	2.803 down
LMO2108	2.085 down
LMO2109	3.013 down
LMO2110	2.525 down
LMO2111	4.316 down
LMO2112	3.366 down

LMO2113	2.251 down
LMO2114	5.570 down
LMO2115	6.676 down
LMO2116	6.097 down
LMO2117	5.368 down
LMO2118	8.375 down
LMO2121	23.635 up
LMO2122	17.860 up
LMO2123	17.192 up
LMO2124	6.687 up
LMO2125	14.136 up
LMO2127	8.309 down
LMO2128	7.858 down
LMO2129	9.538 down
LMO2130	2.921 down
LMO2131	6.739 down
LMO2133	3.051 down
LMO2134	2.224 up
LMO2135	3.002 up
LMO2136	4.674 up
LMO2137	4.918 up
LMO2138	2.632 up
LMO2144	7.601 down
LMO2156	19.914 down
LMO2159	3.858 up
LMO2160	4.678 up
LMO2161	5.420 up
LMO2162	7.140 up
LMO2163	4.940 up
LMO2164	3.122 down
LMO2165	4.530 down
LMO2166	3.586 down
LMO2167	4.796 down
LMO2171	4.328 up
LMO2172	3.028 up
LMO2175	11.559 up
LMO2176	2.722 down
LMO2177	4.719 down
LMO2179	4.559 down
LMO2181	2.375 down
LMO2183	3.141 down
LMO2184	2.899 down
LMO2187	6.786 down
LMO2191	2.466 down
LMO2195	2.921 down
LMO2197	26.870 down
LMO2201	3.116 down
LMO2202	11.536 down

LMO2203	5.019 down
LMO2207	3.832 down
LMO2208	4.914 down
LMO2209	4.502 down
LMO2210	4.537 up
LMO2211	5.242 down
LMO2212	6.354 down
LMO2216	2.160 down
LMO2217	2.221 down
LMO2218	7.653 down
LMO2219	2.097 down
LMO2223	6.357 down
LMO2225	3.321 down
LMO2229	2.110 down
LMO2233	8.219 down
LMO2234	2.086 down
LMO2235	2.531 down
LMO2236	2.781 down
LMO2237	3.296 down
LMO2238	3.234 down
LMO2239	17.244 down
LMO2240	9.975 down
LMO2241	17.022 down
LMO2242	2.815 down
LMO2243	7.507 down
LMO2244	7.010 down
LMO2245	3.588 down
LMO2246	3.249 down
LMO2247	3.422 down
LMO2248	2.554 down
LMO2249	3.009 down
LMO2253	3.599 down
LMO2254	2.762 down
LMO2255	2.259 down
LMO2258	2.358 up
LMO2259	7.594 down
LMO2260	7.659 down
LMO2261	2.613 down
LMO2262	3.059 down
LMO2263	2.813 down
LMO2270	2.764 down
LMO2278	3.033 up
LMO2279	2.002 up
LMO2281	2.399 up
LMO2282	2.290 up
LMO2285	2.063 up
LMO2286	2.261 up
LMO2287	2.144 up

LMO2288	2.511 up
LMO2289	2.047 up
LMO2292	2.000 up
LMO2294	2.142 up
LMO2298	2.563 up
LMO2299	2.742 up
LMO2300	2.019 up
LMO2301	2.780 up
LMO2303	2.974 up
LMO2304	2.331 up
LMO2305	2.353 up
LMO2306	2.759 up
LMO2307	2.176 up
LMO2308	2.406 up
LMO2311	2.328 up
LMO2312	2.002 up
LMO2314	2.149 up
LMO2315	2.405 up
LMO2316	2.104 up
LMO2317	2.253 up
LMO2320	2.712 up
LMO2321	2.449 up
LMO2322	2.217 up
LMO2323	2.013 up
LMO2324	2.252 up
LMO2325	2.295 up
LMO2326	2.602 up
LMO2327	2.428 up
LMO2328	2.025 up
LMO2334	3.199 down
LMO2337	2.700 down
LMO2344	2.434 down
LMO2345	3.195 down
LMO2346	2.963 down
LMO2347	4.540 down
LMO2348	6.239 down
LMO2349	5.964 down
LMO2350	8.358 down
LMO2351	14.777 down
LMO2352	77.367 down
LMO2353	2.591 down
LMO2354	3.753 down
LMO2355	4.781 down
LMO2359	2.154 down
LMO2364	2.596 down
LMO2365	5.004 down
LMO2369	3.805 down
LMO2371	3.513 down

LMO2372	3.150 down
LMO2374	7.454 down
LMO2375	4.651 down
LMO2376	3.454 down
LMO2377	15.914 down
LMO2378	7.010 down
LMO2379	4.573 down
LMO2380	3.651 down
LMO2381	3.614 down
LMO2382	2.013 down
LMO2383	2.292 down
LMO2384	2.662 down
LMO2386	3.269 down
LMO2388	6.216 down
LMO2390	6.054 down
LMO2391	2.256 up
LMO2392	4.269 down
LMO2393	2.370 down
LMO2395	10.597 down
LMO2396	2.831 down
LMO2397	12.483 down
LMO2400	2.894 down
LMO2401	2.873 down
LMO2402	2.389 down
LMO2403	3.363 down
LMO2404	2.130 down
LMO2405	3.315 down
LMO2407	2.205 down
LMO2408	8.346 down
LMO2409	18.291 down
LMO2410	4.216 down
LMO2416	7.623 down
LMO2417	3.323 down
LMO2418	4.356 down
LMO2419	4.779 down
LMO2420	2.294 down
LMO2422	2.050 down
LMO2423	2.574 down
LMO2424	12.433 down
LMO2426	2.118 down
LMO2427	6.723 down
LMO2428	11.633 down
LMO2429	2.093 down
LMO2431	3.083 down
LMO2433	8.814 down
LMO2435	10.074 down
LMO2437	2.096 down
LMO2438	2.806 down

LMO2439	4.660 down
LMO2440	2.530 down
LMO2441	3.516 down
LMO2442	5.342 down
LMO2443	11.332 down
LMO2446	2.075 down
LMO2447	2.563 down
LMO2450	2.223 down
LMO2451	3.565 down
LMO2460	4.472 down
LMO2464	19.310 down
LMO2465	12.356 down
LMO2466	23.777 down
LMO2467	4.725 down
LMO2469	5.495 down
LMO2474	2.785 down
LMO2475	4.053 down
LMO2476	5.703 down
LMO2477	4.028 down
LMO2478	2.226 down
LMO2479	2.215 down
LMO2480	4.289 down
LMO2481	2.405 down
LMO2482	2.696 down
LMO2483	2.925 down
LMO2486	2.402 up
LMO2487	8.833 up
LMO2490	3.193 down
LMO2491	5.100 down
LMO2492	8.223 down
LMO2493	12.713 down
LMO2497	2.019 down
LMO2502	3.594 down
LMO2503	4.485 down
LMO2504	10.859 down
LMO2505	5.283 down
LMO2506	5.763 down
LMO2507	4.759 down
LMO2508	3.234 down
LMO2509	3.025 down
LMO2510	2.388 down
LMO2511	4.051 up
LMO2512	3.935 down
LMO2513	6.232 down
LMO2516	9.072 down
LMO2517	6.383 down
LMO2518	7.121 down
LMO2519	6.836 down

LMO2520	3.975 down
LMO2521	9.508 down
LMO2522	4.623 down
LMO2524	3.235 down
LMO2525	2.990 down
LMO2526	6.865 down
LMO2527	8.051 down
LMO2535	2.062 down
LMO2537	4.218 down
LMO2538	3.870 down
LMO2540	3.464 down
LMO2541	2.267 down
LMO2542	2.147 down
LMO2543	2.515 down
LMO2544	3.794 down
LMO2545	8.347 down
LMO2546	4.609 down
LMO2547	13.696 down
LMO2548	2.291 down
LMO2549	8.644 down
LMO2550	9.249 down
LMO2551	7.722 down
LMO2552	4.115 down
LMO2553	3.107 down
LMO2554	3.035 down
LMO2555	2.283 down
LMO2557	3.443 down
LMO2558	3.296 down
LMO2559	9.650 down
LMO2560	2.461 down
LMO2561	5.161 down
LMO2562	4.962 down
LMO2563	8.605 down
LMO2564	4.150 down
LMO2565	2.849 down
LMO2566	3.867 down
LMO2567	21.833 up
LMO2568	12.415 up
LMO2569	4.195 down
LMO2570	2.521 up
LMO2576	3.222 down
LMO2577	7.649 down
LMO2579	2.622 down
LMO2584	3.892 up
LMO2585	11.917 up
LMO2586	10.877 up
LMO2587	7.533 down
LMO2590	2.106 down

LMO2591	20.004 down
LMO2593	2.135 up
LMO2594	2.592 down
LMO2595	3.099 down
LMO2596	5.748 down
LMO2597	2.566 down
LMO2598	2.439 down
LMO2599	3.584 down
LMO2600	2.821 down
LMO2601	4.233 down
LMO2604	2.210 down
LMO2605	2.731 down
LMO2628	2.175 down
LMO2629	2.088 down
LMO2630	2.050 down
LMO2633	2.267 down
LMO2634	4.298 down
LMO2635	2.508 down
LMO2636	2.354 down
LMO2639	3.436 down
LMO2640	2.727 down
LMO2641	2.693 down
LMO2642	2.074 down
LMO2644	2.217 down
LMO2645	142.954 up
LMO2646	84.283 up
LMO2647	144.279 up
LMO2648	92.415 up
LMO2649	48.264 up
LMO2650	29.323 up
LMO2651	33.793 up
LMO2658	3.866 down
LMO2659	3.035 up
LMO2660	4.089 up
LMO2661	16.768 up
LMO2662	13.639 up
LMO2663	21.145 up
LMO2664	16.266 up
LMO2665	20.652 up
LMO2666	17.375 up
LMO2667	8.907 up
LMO2668	8.715 up
LMO2670	2.899 up
LMO2671	2.377 up
LMO2673	3.022 up
LMO2674	4.027 up
LMO2680	2.308 up
LMO2681	2.615 up

LMO2682	2.860 up
LMO2683	5.149 up
LMO2684	7.871 up
LMO2685	11.108 up
LMO2686	13.178 down
LMO2687	6.297 down
LMO2688	6.261 down
LMO2689	12.132 down
LMO2690	43.573 down
LMO2696	2.110 up
LMO2697	2.230 up
LMO2700	2.273 down
LMO2703	2.115 down
LMO2704	3.086 down
LMO2708	9.915 up
LMO2710	10.464 down
LMO2711	3.311 down
LMO2712	2.311 down
LMO2713	3.901 up
LMO2714	3.557 up
LMO2718	2.063 down
LMO2719	8.847 down
LMO2720	18.115 down
LMO2721	2.589 down
LMO2725	3.205 down
LMO2726	11.039 down
LMO2727	8.202 down
LMO2728	2.029 down
LMO2733	4.004 up
LMO2734	6.173 up
LMO2735	7.069 up
LMO2736	3.193 up
LMO2737	10.125 down
LMO2742	5.680 up
LMO2743	4.035 up
LMO2744	6.674 down
LMO2746	2.365 down
LMO2749	9.334 down
LMO2750	4.252 down
LMO2753	8.684 down
LMO2754	4.402 down
LMO2755	2.108 down
LMO2756	3.713 down
LMO2757	2.775 down
LMO2758	5.229 down
LMO2761	2.004 down
LMO2765	2.263 down
LMO2766	6.440 down

LMO2767	4.135 down
LMO2768	5.786 down
LMO2769	7.340 down
LMO2770	3.247 down
LMO2771	3.346 up
LMO2772	4.320 up
LMO2774	5.646 down
LMO2775	2.706 down
LMO2777	7.495 down
LMO2779	9.225 down
LMO2780	3.593 up
LMO2781	30.740 up
LMO2782	31.364 up
LMO2783	17.189 up
LMO2785	2.119 down
LMO2786	3.615 down
LMO2787	3.786 down
LMO2788	3.059 down
LMO2789	3.421 down
LMO2790	2.450 down
LMO2791	2.972 down
LMO2793	8.263 down
LMO2794	5.549 down
LMO2795	2.831 down
LMO2799	18.475 up
LMO2800	19.916 up
LMO2801	40.473 up
LMO2802	3.386 down
LMO2803	3.195 down
LMO2810	6.275 down
LMO2811	9.541 down
LMO2814	5.111 down
LMO2816	9.502 up
LMO2817	4.167 up
LMO2818	4.878 up
LMO2819	2.698 up
LMO2820	2.829 down
LMO2823	2.441 down
LMO2826	7.378 down
LMO2827	6.883 down
LMO2829	9.549 down
LMO2830	4.195 down
LMO2831	3.806 down
LMO2832	2.397 down
LMO2833	2.287 down
LMO2840	2.574 down
LMO2841	4.629 down
LMO2842	6.577 down

LMO2843	5.427	down
LMO2844	5.491	down
LMO2845	6.991	down
LMO2846	3.303	up
LMO2847	5.645	up
LMO2848	12.426	up
LMO2849	17.831	up
LMO2850	21.620	up
LMO2851	5.417	up
LMO2852	10.756	down
LMO2853	2.314	down
LMO2854	3.212	down
LMO2855	4.737	down
LMO2856	11.488	down
LMO2857	12.593	down
