# Supplementary material for: Changes in Gene Expression during Adaptation of Listeria monocytogenes to the Soil Environment
Source: PLoS One. 2011 Sep 23;6(9):e24881. doi: 10.1371/journal.pone.0024881 (PMC3179493; doi:10.1371/journal.pone.0024881)
Supplement: Table S3 — Genes over 2-fold change at time 18 hours. (PDF) [file pone.0024881.s003.pdf]

Table S3. Genes over 2-fold change at time 18 hours.

LMO0001	3.071 down
LMO0002	6.355 down
LMO0003	10.910 down
LMO0005	4.527 down
LMO0006	2.267 down
LMO0007	3.728 down
LMO0008	4.857 down
LMO0009	8.731 down
LMO0010	4.970 down
LMO0011	2.333 down
LMO0012	3.235 down
LMO0013	6.724 down
LMO0014	4.371 down
LMO0015	5.296 down
LMO0016	10.232 down
LMO0017	10.287 down
LMO0021	4.009 up
LMO0022	4.137 up
LMO0023	7.562 up
LMO0024	7.031 up
LMO0026	3.155 down
LMO0027	21.795 up
LMO0028	5.671 down
LMO0029	8.831 down
LMO0030	2.333 down
LMO0031	5.518 down
LMO0033	4.351 down
LMO0034	5.929 down
LMO0035	6.106 down
LMO0036	2.636 down
LMO0037	7.747 down
LMO0040	7.806 down
LMO0041	10.142 down
LMO0042	16.606 down
LMO0046	11.363 down
LMO0047	3.534 down
LMO0048	3.936 down
LMO0049	6.418 down
LMO0050	10.258 down
LMO0051	7.118 down
LMO0053	2.191 down
LMO0055	43.181 down
LMO0057	4.811 down

LMO0058	3.616 down
LMO0059	2.502 down
LMO0060	3.114 down
LMO0061	3.469 down
LMO0062	2.716 down
LMO0064	2.958 down
LMO0065	2.647 down
LMO0066	3.429 down
LMO0067	5.198 down
LMO0068	5.261 down
LMO0069	5.076 down
LMO0070	5.219 down
LMO0071	16.778 down
LMO0072	16.010 down
LMO0073	8.568 down
LMO0074	8.355 down
LMO0075	31.929 down
LMO0076	7.530 down
LMO0077	4.588 down
LMO0078	2.991 down
LMO0079	10.029 down
LMO0080	9.374 down
LMO0081	4.261 down
LMO0082	2.781 down
LMO0084	6.433 down
LMO0086	3.727 down
LMO0089	3.177 down
LMO0090	3.086 down
LMO0091	2.733 down
LMO0093	2.469 down
LMO0094	2.726 down
LMO0095	4.093 down
LMO0099	11.313 down
LMO0100	3.343 down
LMO0101	5.766 down
LMO0102	3.492 down
LMO0103	4.370 down
LMO0104	3.329 down
LMO0105	15.998 up
LMO0106	11.713 down
LMO0107	9.974 down
LMO0109	2.879 down
LMO0110	2.974 down
LMO0111	18.558 down
LMO0112	8.199 down
LMO0115	2.029 up
LMO0117	2.610 up
LMO0118	2.523 up

LMO0119	3.265 up
LMO0120	2.601 up
LMO0121	2.498 up
LMO0123	3.303 up
LMO0124	2.080 up
LMO0126	2.442 up
LMO0127	3.154 up
LMO0128	3.229 up
LMO0130	3.091 down
LMO0132	3.093 down
LMO0135	2.518 down
LMO0136	3.662 down
LMO0137	3.326 down
LMO0138	7.507 down
LMO0139	10.033 down
LMO0140	11.060 down
LMO0142	5.365 down
LMO0143	3.172 down
LMO0144	5.162 down
LMO0145	4.978 down
LMO0146	2.975 down
LMO0149	4.736 down
LMO0150	2.995 down
LMO0151	16.895 down
LMO0152	11.048 down
LMO0153	82.501 down
LMO0154	29.400 down
LMO0155	10.502 down
LMO0156	4.457 down
LMO0157	3.340 down
LMO0158	11.662 down
LMO0159	7.537 down
LMO0160	6.111 down
LMO0161	4.152 down
LMO0162	4.081 down
LMO0164	3.046 down
LMO0165	3.823 down
LMO0166	3.839 down
LMO0167	5.977 down
LMO0168	9.016 down
LMO0169	6.332 down
LMO0171	3.771 down
LMO0173	4.279 down
LMO0175	2.102 down
LMO0176	9.874 down
LMO0177	3.276 down
LMO0178	5.160 down
LMO0179	2.407 down

LMO0180	2.132	down
LMO0182	2.056	down
LMO0183	3.922	down
LMO0184	5.471	down
LMO0185	16.929	down
LMO0186	11.772	down
LMO0188	4.852	down
LMO0189	4.045	down
LMO0190	5.057	down
LMO0191	2.274	down
LMO0192	8.297	down
LMO0193	3.410	down
LMO0194	2.566	down
LMO0197	2.195	down
LMO0198	8.011	down
LMO0199	9.697	down
LMO0200	2.253	down
LMO0201	3.359	down
LMO0202	3.517	down
LMO0204	5.819	down
LMO0207	6.211	down
LMO0208	10.323	down
LMO0209	4.221	down
LMO0212	3.089	down
LMO0213	7.527	down
LMO0214	2.482	down
LMO0217	12.409	down
LMO0218	6.685	down
LMO0219	6.995	down
LMO0221	5.521	down
LMO0222	5.794	down
LMO0223	3.050	down
LMO0224	2.790	down
LMO0225	3.663	down
LMO0226	4.219	down
LMO0227	3.171	down
LMO0228	2.244	down
LMO0229	2.275	down
LMO0232	2.081	down
LMO0233	2.123	down
LMO0238	11.187	down
LMO0239	5.446	down
LMO0240	4.685	down
LMO0241	13.331	down
LMO0242	3.627	down
LMO0243	6.566	down
LMO0244	15.534	down
LMO0245	17.135	down

LMO0246	2.849	down
LMO0247	6.264	down
LMO0248	4.890	down
LMO0249	3.093	down
LMO0250	2.498	down
LMO0251	2.690	down
LMO0252	16.323	down
LMO0253	6.005	down
LMO0254	2.195	down
LMO0256	3.403	down
LMO0258	2.068	down
LMO0260	17.619	down
LMO0262	4.713	down
LMO0266	2.344	down
LMO0268	4.987	down
LMO0269	19.905	down
LMO0270	3.684	down
LMO0271	2.597	down
LMO0272	5.097	down
LMO0273	12.275	down
LMO0275	2.216	down
LMO0276	4.498	down
LMO0277	3.225	down
LMO0280	2.648	up
LMO0281	5.345	down
LMO0282	12.757	down
LMO0283	39.564	down
LMO0284	35.908	down
LMO0285	29.762	down
LMO0286	11.340	down
LMO0288	4.966	down
LMO0289	5.982	down
LMO0290	3.299	down
LMO0291	3.171	down
LMO0293	8.829	down
LMO0294	12.327	down
LMO0295	3.856	down
LMO0296	24.589	down
LMO0298	5.698	up
LMO0299	8.432	up
LMO0300	6.092	up
LMO0301	4.396	up
LMO0302	10.871	down
LMO0303	13.426	down
LMO0304	18.584	down
LMO0306	3.294	down
LMO0307	2.191	down
LMO0310	2.088	down

LMO0312	7.815	down
LMO0313	11.375	down
LMO0314	4.700	down
LMO0315	2.581	down
LMO0316	8.150	down
LMO0317	4.127	down
LMO0318	11.517	down
LMO0320	2.949	down
LMO0321	16.598	down
LMO0322	4.274	down
LMO0325	4.310	down
LMO0326	3.746	down
LMO0327	2.963	down
LMO0331	5.816	down
LMO0332	8.965	down
LMO0333	6.899	down
LMO0335	2.262	down
LMO0336	4.865	down
LMO0337	8.798	down
LMO0338	10.322	down
LMO0339	2.826	down
LMO0344	3.672	up
LMO0346	2.504	up
LMO0348	3.171	up
LMO0352	3.106	down
LMO0353	5.933	down
LMO0354	4.966	down
LMO0356	4.147	down
LMO0359	3.683	down
LMO0360	5.705	down
LMO0361	29.235	down
LMO0362	4.723	down
LMO0363	4.484	down
LMO0364	10.055	down
LMO0365	8.236	down
LMO0366	8.401	down
LMO0367	5.772	down
LMO0368	5.874	down
LMO0369	2.285	down
LMO0370	3.910	down
LMO0371	7.891	down
LMO0375	4.529	down
LMO0376	2.735	down
LMO0377	5.428	down
LMO0382	5.791	down
LMO0385	2.190	down
LMO0387	4.633	down
LMO0388	5.127	down

LMO0389	52.996 down
LMO0390	15.304 down
LMO0391	4.027 down
LMO0392	2.138 down
LMO0394	26.538 down
LMO0395	12.235 down
LMO0396	9.362 down
LMO0397	3.216 down
LMO0400	3.501 up
LMO0401	5.745 up
LMO0403	2.798 down
LMO0404	6.903 down
LMO0405	2.713 down
LMO0406	8.219 down
LMO0407	2.848 down
LMO0409	5.097 down
LMO0410	14.191 down
LMO0411	3.051 down
LMO0412	2.799 down
LMO0413	6.194 down
LMO0414	5.517 down
LMO0415	7.041 down
LMO0417	3.724 down
LMO0419	4.010 down
LMO0420	3.172 down
LMO0422	3.241 down
LMO0425	3.755 down
LMO0426	2.534 down
LMO0427	2.405 down
LMO0429	3.227 down
LMO0430	13.421 down
LMO0431	5.395 down
LMO0432	2.167 down
LMO0435	9.142 down
LMO0436	15.826 down
LMO0438	2.491 down
LMO0439	3.765 down
LMO0440	8.689 down
LMO0441	5.072 down
LMO0442	3.015 down
LMO0444	9.365 down
LMO0448	3.246 down
LMO0449	14.827 down
LMO0450	6.128 down
LMO0451	3.408 down
LMO0452	9.194 down
LMO0453	9.388 down
LMO0454	11.313 down

LMO0455	11.272	down
LMO0456	5.724	down
LMO0457	7.949	down
LMO0458	7.420	down
LMO0459	3.617	down
LMO0460	2.430	down
LMO0462	3.520	down
LMO0463	3.940	down
LMO0465	6.807	down
LMO0466	5.272	down
LMO0467	4.111	down
LMO0468	5.590	down
LMO0469	28.622	down
LMO0470	11.662	down
LMO0472	6.338	down
LMO0473	9.972	down
LMO0474	10.628	down
LMO0475	4.101	down
LMO0476	5.185	down
LMO0477	81.494	down
LMO0478	23.585	down
LMO0479	13.266	down
LMO0480	9.079	down
LMO0482	4.612	down
LMO0483	4.347	down
LMO0484	7.722	down
LMO0485	20.053	down
LMO0486	6.699	down
LMO0487	2.192	down
LMO0488	2.205	down
LMO0489	2.042	down
LMO0490	4.509	down
LMO0491	9.566	down
LMO0492	10.454	down
LMO0493	6.585	down
LMO0494	3.138	down
LMO0495	6.287	down
LMO0497	28.157	down
LMO0498	2.243	down
LMO0499	2.242	down
LMO0500	3.134	down
LMO0501	2.570	down
LMO0509	5.219	down
LMO0510	9.999	down
LMO0511	12.254	down
LMO0512	4.020	down
LMO0513	7.318	down
LMO0514	5.895	down

LMO0515	3.180 up
LMO0516	5.289 down
LMO0518	5.983 down
LMO0519	9.048 down
LMO0520	2.888 down
LMO0522	12.208 down
LMO0523	6.182 down
LMO0524	3.361 down
LMO0525	18.281 down
LMO0526	4.381 down
LMO0527	14.697 down
LMO0528	7.717 down
LMO0529	3.532 down
LMO0531	2.050 down
LMO0532	5.838 down
LMO0533	17.260 down
LMO0534	2.774 down
LMO0535	2.822 down
LMO0537	10.023 down
LMO0538	4.970 down
LMO0540	10.682 up
LMO0541	6.293 down
LMO0544	6.960 up
LMO0545	2.105 up
LMO0547	10.557 down
LMO0548	2.727 down
LMO0549	4.160 down
LMO0550	7.605 down
LMO0551	8.341 down
LMO0552	10.186 down
LMO0554	2.488 down
LMO0557	4.664 down
LMO0558	7.197 down
LMO0559	13.487 down
LMO0560	11.762 down
LMO0563	4.101 down
LMO0564	4.625 down
LMO0565	2.737 down
LMO0566	5.321 down
LMO0567	4.868 down
LMO0568	4.459 down
LMO0569	8.891 down
LMO0570	5.443 down
LMO0571	5.379 down
LMO0572	2.660 down
LMO0573	7.285 down
LMO0574	2.974 down
LMO0575	5.011 down

LMO0576	7.830	down
LMO0577	3.330	down
LMO0578	2.152	down
LMO0579	2.782	down
LMO0581	8.487	down
LMO0582	20.543	down
LMO0583	5.909	down
LMO0584	3.542	up
LMO0585	4.591	down
LMO0586	9.111	down
LMO0587	4.150	down
LMO0588	11.567	down
LMO0589	8.257	down
LMO0590	4.468	down
LMO0591	2.737	down
LMO0592	3.377	down
LMO0593	3.349	down
LMO0594	7.981	down
LMO0595	7.006	down
LMO0597	11.580	down
LMO0598	11.774	down
LMO0599	14.452	down
LMO0600	15.093	down
LMO0601	8.837	down
LMO0603	4.902	down
LMO0604	6.608	down
LMO0605	4.434	down
LMO0606	5.052	down
LMO0608	2.013	down
LMO0609	7.232	down
LMO0611	21.554	down
LMO0612	3.279	down
LMO0613	2.338	down
LMO0614	13.731	down
LMO0615	7.607	down
LMO0616	10.673	down
LMO0617	4.460	down
LMO0618	9.597	down
LMO0619	4.057	down
LMO0620	4.599	down
LMO0621	5.149	down
LMO0623	6.355	down
LMO0626	6.432	down
LMO0627	3.511	down
LMO0629	2.676	down
LMO0632	2.038	down
LMO0635	13.306	down
LMO0636	5.212	down

LMO0637	5.800 down
LMO0638	15.790 down
LMO0639	4.240 down
LMO0642	6.780 down
LMO0643	3.187 up
LMO0644	7.361 down
LMO0645	9.814 down
LMO0646	3.697 down
LMO0647	3.461 down
LMO0648	8.156 down
LMO0649	5.101 down
LMO0650	2.246 down
LMO0652	3.447 down
LMO0654	4.767 down
LMO0655	2.919 down
LMO0656	29.867 down
LMO0657	4.965 down
LMO0658	40.813 down
LMO0659	5.918 down
LMO0661	2.313 down
LMO0662	9.287 down
LMO0663	2.734 down
LMO0664	12.971 down
LMO0665	8.155 down
LMO0666	8.345 down
LMO0667	3.898 down
LMO0668	6.453 down
LMO0669	2.779 down
LMO0670	2.305 down
LMO0671	6.657 down
LMO0672	3.763 down
LMO0673	71.540 up
LMO0674	2.817 down
LMO0675	5.291 down
LMO0676	2.740 down
LMO0677	7.065 down
LMO0678	9.492 down
LMO0679	94.171 down
LMO0680	19.583 down
LMO0681	7.523 down
LMO0682	6.984 down
LMO0683	41.616 down
LMO0684	14.620 down
LMO0685	7.425 down
LMO0686	3.299 down
LMO0687	5.893 down
LMO0688	2.480 down
LMO0691	6.752 down

LMO0692	2.386	down
LMO0694	4.623	down
LMO0695	5.065	down
LMO0696	5.936	down
LMO0700	2.133	down
LMO0701	6.617	down
LMO0702	3.474	down
LMO0704	2.421	down
LMO0705	2.575	down
LMO0707	4.217	down
LMO0708	2.271	down
LMO0709	2.549	down
LMO0710	2.375	down
LMO0712	2.561	down
LMO0713	2.548	down
LMO0714	2.016	down
LMO0715	2.222	down
LMO0718	3.947	down
LMO0721	8.926	down
LMO0725	16.554	down
LMO0726	12.910	down
LMO0727	10.975	down
LMO0728	10.561	down
LMO0729	14.208	down
LMO0730	4.295	down
LMO0731	3.536	down
LMO0732	5.653	down
LMO0733	13.809	down
LMO0734	11.564	down
LMO0738	3.892	down
LMO0739	21.550	down
LMO0740	11.000	down
LMO0742	2.236	down
LMO0744	2.238	down
LMO0745	5.736	down
LMO0749	4.402	down
LMO0750	4.700	down
LMO0751	3.037	down
LMO0752	3.584	down
LMO0753	3.413	down
LMO0754	3.762	down
LMO0755	6.595	down
LMO0756	8.095	down
LMO0757	5.098	down
LMO0758	3.831	down
LMO0759	2.693	down
LMO0762	6.146	down
LMO0763	5.132	down

LMO0764	7.875 down
LMO0765	7.630 down
LMO0767	3.504 down
LMO0768	5.303 down
LMO0769	7.872 down
LMO0770	5.171 down
LMO0771	17.565 down
LMO0772	11.845 down
LMO0773	6.416 down
LMO0774	6.529 down
LMO0776	7.082 down
LMO0777	11.035 down
LMO0778	13.198 down
LMO0779	9.120 down
LMO0781	4.114 down
LMO0785	5.111 down
LMO0786	2.726 down
LMO0787	8.114 down
LMO0788	3.864 down
LMO0790	25.194 down
LMO0791	9.086 down
LMO0792	2.846 down
LMO0793	9.124 down
LMO0795	6.842 down
LMO0796	3.355 down
LMO0797	4.466 down
LMO0799	3.906 down
LMO0801	4.798 down
LMO0802	9.114 down
LMO0803	11.525 down
LMO0804	8.926 down
LMO0806	5.984 down
LMO0807	3.865 down
LMO0808	4.646 down
LMO0809	3.155 down
LMO0810	2.371 down
LMO0811	2.916 down
LMO0812	6.310 down
LMO0813	2.017 down
LMO0814	18.482 down
LMO0815	7.891 down
LMO0816	7.123 down
LMO0817	12.343 down
LMO0818	4.968 down
LMO0819	2.666 down
LMO0820	11.999 down
LMO0821	9.341 down
LMO0822	8.803 down

LMO0824	6.932	down
LMO0825	3.434	down
LMO0826	6.136	down
LMO0829	2.164	down
LMO0830	6.174	down
LMO0831	10.877	down
LMO0833	5.734	down
LMO0834	11.066	down
LMO0835	26.350	down
LMO0836	43.153	down
LMO0837	8.412	down
LMO0839	4.996	down
LMO0840	24.753	down
LMO0841	8.905	down
LMO0842	4.791	down
LMO0844	6.310	down
LMO0845	7.794	down
LMO0846	6.763	down
LMO0847	8.391	down
LMO0848	19.234	down
LMO0849	2.809	down
LMO0850	10.299	down
LMO0851	6.077	down
LMO0852	12.156	down
LMO0853	6.340	down
LMO0854	6.521	down
LMO0855	7.246	down
LMO0856	3.697	down
LMO0857	6.348	down
LMO0859	2.634	down
LMO0860	4.061	down
LMO0861	4.158	down
LMO0863	3.741	down
LMO0864	5.967	down
LMO0866	6.383	down
LMO0867	9.354	down
LMO0868	4.008	down
LMO0870	3.215	down
LMO0871	2.924	down
LMO0877	2.242	up
LMO0879	2.074	up
LMO0881	2.073	down
LMO0882	17.175	down
LMO0883	30.168	down
LMO0884	4.599	down
LMO0885	5.247	down
LMO0886	3.145	down
LMO0887	8.836	down

LMO0888	20.393 down
LMO0889	7.400 down
LMO0890	9.746 down
LMO0891	7.931 down
LMO0892	2.465 down
LMO0893	3.524 down
LMO0894	2.833 down
LMO0896	2.410 down
LMO0897	2.284 down
LMO0899	2.505 down
LMO0900	11.736 down
LMO0901	2.955 down
LMO0902	8.679 down
LMO0903	6.121 down
LMO0904	14.118 down
LMO0905	9.305 down
LMO0906	2.745 down
LMO0907	2.689 down
LMO0908	20.232 down
LMO0909	21.304 down
LMO0910	6.115 down
LMO0911	2.785 down
LMO0912	11.266 down
LMO0914	3.314 up
LMO0915	5.686 up
LMO0916	11.860 up
LMO0917	16.982 up
LMO0919	2.598 down
LMO0920	8.843 down
LMO0921	7.439 down
LMO0922	9.214 down
LMO0923	5.644 down
LMO0924	7.500 down
LMO0925	8.493 down
LMO0926	5.787 down
LMO0927	7.548 down
LMO0928	31.878 down
LMO0929	2.347 down
LMO0931	2.925 down
LMO0932	4.086 down
LMO0933	3.065 down
LMO0934	3.656 down
LMO0935	8.455 down
LMO0938	2.506 down
LMO0939	31.920 down
LMO0940	2.144 down
LMO0941	4.425 down
LMO0944	4.114 down

LMO0945	2.610 down
LMO0947	4.400 down
LMO0948	6.579 down
LMO0949	3.416 down
LMO0950	6.325 down
LMO0951	7.432 down
LMO0952	6.264 down
LMO0955	2.219 up
LMO0957	2.481 up
LMO0959	30.561 down
LMO0960	2.394 down
LMO0961	3.809 down
LMO0964	4.448 down
LMO0965	4.118 down
LMO0966	6.981 down
LMO0967	6.577 down
LMO0968	6.255 down
LMO0969	7.154 down
LMO0970	6.227 down
LMO0971	5.483 down
LMO0972	13.912 down
LMO0973	3.479 down
LMO0974	7.100 down
LMO0975	5.430 down
LMO0976	6.230 down
LMO0977	6.980 down
LMO0978	6.250 down
LMO0979	2.096 down
LMO0980	2.668 down
LMO0981	3.440 down
LMO0982	2.248 down
LMO0988	2.383 down
LMO0989	2.605 down
LMO0990	7.719 down
LMO0995	6.601 down
LMO0998	29.424 down
LMO0999	7.750 down
LMO1000	23.386 down
LMO1001	20.720 down
LMO1002	2.410 down
LMO1003	3.934 down
LMO1004	6.430 down
LMO1005	2.825 down
LMO1006	4.148 down
LMO1008	8.797 down
LMO1009	6.287 down
LMO1010	4.672 down
LMO1011	3.560 down

LMO1012	3.382 down
LMO1013	3.967 down
LMO1014	6.031 down
LMO1015	7.573 down
LMO1016	7.890 down
LMO1018	9.491 down
LMO1019	4.463 down
LMO1020	3.625 down
LMO1021	3.054 down
LMO1022	5.623 down
LMO1023	5.248 down
LMO1024	14.718 down
LMO1025	13.662 down
LMO1026	7.140 down
LMO1027	9.197 down
LMO1028	3.435 down
LMO1029	3.831 down
LMO1030	8.665 down
LMO1031	5.855 down
LMO1032	2.963 down
LMO1033	3.050 down
LMO1034	3.128 down
LMO1035	5.534 down
LMO1036	3.358 down
LMO1037	5.463 down
LMO1038	12.933 down
LMO1039	6.285 down
LMO1040	2.625 down
LMO1041	10.514 down
LMO1042	2.705 down
LMO1044	2.855 down
LMO1045	2.505 down
LMO1046	2.629 down
LMO1048	2.021 down
LMO1050	6.381 down
LMO1054	2.336 up
LMO1056	7.320 down
LMO1057	3.991 down
LMO1058	4.722 down
LMO1059	4.387 down
LMO1060	9.010 down
LMO1061	4.765 down
LMO1063	2.120 down
LMO1064	12.369 down
LMO1065	16.539 down
LMO1066	7.204 down
LMO1067	4.605 down
LMO1069	8.220 down

LMO1070	7.216 down
LMO1071	10.246 down
LMO1072	2.509 down
LMO1073	30.015 down
LMO1074	12.147 down
LMO1075	6.615 down
LMO1076	7.749 down
LMO1077	3.007 down
LMO1078	12.284 down
LMO1079	14.248 down
LMO1080	6.452 down
LMO1081	2.158 down
LMO1082	5.378 down
LMO1083	4.321 down
LMO1084	22.054 down
LMO1085	4.803 down
LMO1086	5.814 down
LMO1088	13.644 down
LMO1089	11.310 down
LMO1090	8.399 down
LMO1091	7.271 down
LMO1092	6.972 down
LMO1093	5.086 down
LMO1094	2.101 down
LMO1095	3.828 down
LMO1096	3.159 down
LMO1097	4.220 down
LMO1100	7.335 down
LMO1110	2.306 up
LMO1116	5.501 down
LMO1117	4.506 down
LMO1118	15.864 down
LMO1119	20.207 down
LMO1120	20.539 down
LMO1121	2.534 down
LMO1122	11.361 down
LMO1123	6.531 down
LMO1124	9.847 down
LMO1125	9.758 down
LMO1126	7.828 down
LMO1127	16.165 down
LMO1128	6.915 down
LMO1129	8.244 down
LMO1130	16.588 down
LMO1131	4.761 down
LMO1132	2.881 down
LMO1134	9.129 down
LMO1135	13.881 down

LMO1136	5.644	down
LMO1137	2.484	down
LMO1141	8.313	down
LMO1142	2.966	down
LMO1143	2.354	down
LMO1144	4.780	down
LMO1145	6.850	down
LMO1146	4.665	down
LMO1147	6.519	down
LMO1149	6.092	down
LMO1150	4.778	down
LMO1153	2.175	down
LMO1155	2.477	down
LMO1156	2.709	down
LMO1157	2.012	down
LMO1158	2.867	down
LMO1159	4.511	down
LMO1161	2.989	down
LMO1162	7.624	down
LMO1163	3.167	down
LMO1165	4.464	down
LMO1169	6.557	down
LMO1170	6.896	down
LMO1171	2.157	down
LMO1172	6.946	down
LMO1173	5.468	down
LMO1175	4.011	down
LMO1176	2.622	down
LMO1177	2.525	down
LMO1178	3.553	down
LMO1179	5.553	down
LMO1180	6.402	down
LMO1181	8.766	down
LMO1182	5.642	down
LMO1183	6.203	down
LMO1184	6.879	down
LMO1185	6.004	down
LMO1186	4.312	down
LMO1188	6.409	down
LMO1189	2.286	down
LMO1190	5.512	down
LMO1191	2.143	down
LMO1192	6.445	down
LMO1193	8.025	down
LMO1194	8.384	down
LMO1195	5.615	down
LMO1196	7.249	down
LMO1197	3.448	down

LMO1198	5.068	down
LMO1199	8.003	down
LMO1200	7.246	down
LMO1201	4.884	down
LMO1202	12.155	down
LMO1204	10.919	down
LMO1205	9.210	down
LMO1206	14.025	down
LMO1207	5.886	down
LMO1209	6.809	down
LMO1210	47.665	down
LMO1211	11.291	down
LMO1212	9.153	down
LMO1213	14.621	down
LMO1214	6.372	down
LMO1215	4.447	down
LMO1216	3.494	down
LMO1217	2.069	down
LMO1219	3.822	down
LMO1220	2.852	down
LMO1221	5.394	down
LMO1222	3.927	down
LMO1223	13.097	down
LMO1224	8.333	down
LMO1225	18.708	down
LMO1226	2.953	down
LMO1227	12.993	down
LMO1228	17.092	down
LMO1229	12.188	down
LMO1230	9.520	down
LMO1231	5.857	down
LMO1232	5.475	down
LMO1233	3.072	down
LMO1234	2.244	down
LMO1235	8.277	down
LMO1236	7.688	down
LMO1237	14.616	down
LMO1238	7.628	down
LMO1239	25.557	down
LMO1240	7.340	down
LMO1242	3.624	down
LMO1243	5.010	down
LMO1244	5.903	down
LMO1245	13.354	down
LMO1246	10.544	down
LMO1247	15.452	down
LMO1248	36.775	down
LMO1249	14.113	down

LMO1250	5.058 down
LMO1251	21.166 down
LMO1252	10.095 down
LMO1253	7.967 down
LMO1257	2.905 down
LMO1258	4.197 down
LMO1259	5.240 down
LMO1260	5.847 down
LMO1261	2.920 down
LMO1262	8.595 down
LMO1266	9.753 down
LMO1267	3.963 down
LMO1268	6.035 down
LMO1269	5.292 down
LMO1270	7.755 down
LMO1271	11.615 down
LMO1272	13.355 down
LMO1273	6.960 down
LMO1274	5.559 down
LMO1275	19.497 down
LMO1276	13.222 down
LMO1277	2.082 down
LMO1278	4.089 down
LMO1279	2.926 down
LMO1280	13.992 down
LMO1281	4.936 down
LMO1282	6.040 down
LMO1283	4.778 down
LMO1284	8.313 down
LMO1285	4.574 down
LMO1286	5.451 down
LMO1287	2.360 down
LMO1288	3.251 down
LMO1290	3.610 down
LMO1291	12.478 down
LMO1292	14.397 down
LMO1293	2.372 up
LMO1294	12.130 down
LMO1296	10.527 down
LMO1297	6.302 down
LMO1298	39.342 down
LMO1299	4.606 down
LMO1300	16.732 down
LMO1303	2.365 down
LMO1304	3.022 down
LMO1305	3.573 down
LMO1306	8.986 down
LMO1307	13.148 down

LMO1308	7.122 down
LMO1309	7.743 down
LMO1310	24.779 down
LMO1311	22.252 down
LMO1312	12.965 down
LMO1313	5.192 down
LMO1314	6.758 down
LMO1315	5.716 down
LMO1316	9.142 down
LMO1317	4.989 down
LMO1318	4.015 down
LMO1319	4.248 down
LMO1320	4.132 down
LMO1321	5.278 down
LMO1322	3.839 down
LMO1323	2.086 down
LMO1324	4.054 down
LMO1325	2.759 down
LMO1326	5.703 down
LMO1327	10.307 down
LMO1328	9.418 down
LMO1329	5.531 down
LMO1330	6.377 down
LMO1331	13.323 down
LMO1333	11.753 down
LMO1334	9.650 down
LMO1335	7.007 down
LMO1336	2.486 down
LMO1337	8.512 down
LMO1338	7.868 down
LMO1339	6.695 down
LMO1340	3.225 down
LMO1341	3.775 down
LMO1342	2.064 down
LMO1347	3.465 down
LMO1349	2.276 up
LMO1350	2.059 up
LMO1351	3.082 down
LMO1353	21.253 down
LMO1354	4.369 down
LMO1355	5.444 down
LMO1356	5.440 down
LMO1357	4.106 down
LMO1358	3.698 down
LMO1359	6.154 down
LMO1360	6.689 down
LMO1361	3.989 down
LMO1362	5.373 down

LMO1363	9.724 down
LMO1364	3.418 down
LMO1365	5.336 down
LMO1366	7.097 down
LMO1367	16.894 down
LMO1368	7.501 down
LMO1369	12.563 down
LMO1370	7.792 down
LMO1371	3.084 down
LMO1372	3.491 down
LMO1373	3.433 down
LMO1374	2.129 down
LMO1375	9.213 down
LMO1376	2.311 down
LMO1377	6.652 down
LMO1378	6.594 down
LMO1379	2.259 down
LMO1380	4.209 down
LMO1381	3.634 down
LMO1382	4.833 down
LMO1383	3.578 down
LMO1384	10.791 down
LMO1385	9.437 down
LMO1386	3.668 down
LMO1387	7.201 down
LMO1388	2.075 down
LMO1389	2.544 down
LMO1390	2.640 down
LMO1391	2.494 down
LMO1392	7.623 down
LMO1393	3.891 down
LMO1394	6.161 down
LMO1395	53.343 down
LMO1396	14.172 down
LMO1397	5.582 down
LMO1398	2.881 down
LMO1399	5.100 down
LMO1400	8.232 down
LMO1401	11.751 down
LMO1402	7.130 down
LMO1403	7.072 down
LMO1404	2.895 down
LMO1406	3.491 down
LMO1407	2.541 down
LMO1408	4.711 down
LMO1409	10.877 down
LMO1410	9.947 down
LMO1411	7.260 down

LMO1412	5.795 down
LMO1413	6.892 down
LMO1414	3.549 down
LMO1415	4.021 down
LMO1416	11.381 down
LMO1417	19.524 down
LMO1418	14.923 down
LMO1419	16.131 down
LMO1420	14.839 down
LMO1421	3.600 down
LMO1422	2.028 down
LMO1423	4.158 down
LMO1424	2.292 down
LMO1425	3.017 down
LMO1426	2.095 down
LMO1427	4.858 down
LMO1428	15.392 down
LMO1429	18.686 down
LMO1430	5.502 down
LMO1431	12.551 down
LMO1432	4.240 down
LMO1433	2.659 down
LMO1434	2.984 down
LMO1435	5.875 down
LMO1436	5.219 down
LMO1437	10.189 down
LMO1438	4.547 down
LMO1439	2.790 down
LMO1440	36.429 down
LMO1441	13.223 down
LMO1442	12.369 down
LMO1443	6.018 down
LMO1445	14.079 down
LMO1446	25.736 down
LMO1447	17.351 down
LMO1448	3.596 down
LMO1449	8.315 down
LMO1450	4.676 down
LMO1451	6.459 down
LMO1452	2.403 down
LMO1453	4.750 down
LMO1455	2.280 down
LMO1456	2.685 down
LMO1457	4.268 down
LMO1458	3.476 down
LMO1459	5.587 down
LMO1460	3.001 down
LMO1461	9.219 down

LMO1462	8.493 down
LMO1463	8.486 down
LMO1464	11.767 down
LMO1465	8.106 down
LMO1466	7.548 down
LMO1467	6.237 down
LMO1468	3.620 down
LMO1470	2.229 down
LMO1474	2.433 down
LMO1476	10.091 down
LMO1477	8.345 down
LMO1478	2.041 down
LMO1479	5.365 down
LMO1480	7.470 down
LMO1481	5.043 down
LMO1483	9.383 down
LMO1484	2.686 down
LMO1485	16.856 down
LMO1486	13.877 down
LMO1487	10.840 down
LMO1488	7.557 down
LMO1489	9.404 down
LMO1490	6.990 down
LMO1491	7.689 down
LMO1492	15.308 down
LMO1493	3.178 down
LMO1494	7.282 down
LMO1495	7.813 down
LMO1496	11.797 down
LMO1497	6.340 down
LMO1498	19.316 down
LMO1499	15.563 down
LMO1500	9.605 down
LMO1501	3.417 down
LMO1503	2.331 down
LMO1504	9.227 down
LMO1505	3.025 down
LMO1506	5.894 down
LMO1507	4.573 down
LMO1508	5.069 down
LMO1509	2.201 down
LMO1510	5.178 down
LMO1511	3.828 down
LMO1512	7.249 down
LMO1513	5.504 down
LMO1514	3.045 down
LMO1515	17.312 down
LMO1516	10.918 down

LMO1517	10.387 down
LMO1518	4.462 down
LMO1519	124.440 down
LMO1520	11.362 down
LMO1521	5.823 down
LMO1522	6.828 down
LMO1523	3.866 down
LMO1524	7.658 down
LMO1525	9.452 down
LMO1526	2.440 down
LMO1527	2.644 down
LMO1528	12.057 down
LMO1529	4.051 down
LMO1530	3.940 down
LMO1531	3.339 down
LMO1532	3.663 down
LMO1533	3.389 down
LMO1534	3.914 down
LMO1535	2.938 down
LMO1536	9.551 down
LMO1537	7.686 down
LMO1543	6.039 down
LMO1544	4.413 down
LMO1545	13.713 down
LMO1546	5.227 down
LMO1547	3.838 down
LMO1548	3.454 down
LMO1549	7.523 down
LMO1550	3.255 down
LMO1551	11.825 down
LMO1552	5.479 down
LMO1553	2.016 down
LMO1554	7.009 down
LMO1555	16.817 down
LMO1556	10.509 down
LMO1557	11.587 down
LMO1558	14.111 down
LMO1559	8.434 down
LMO1560	5.917 down
LMO1561	5.281 down
LMO1562	3.554 down
LMO1563	4.106 down
LMO1564	4.642 down
LMO1565	2.397 down
LMO1566	7.996 down
LMO1567	3.624 down
LMO1568	40.628 down
LMO1569	7.156 down

LMO1571	4.034 down
LMO1572	3.974 down
LMO1573	2.655 down
LMO1574	3.782 down
LMO1575	9.555 down
LMO1576	8.078 down
LMO1577	7.267 down
LMO1578	4.431 down
LMO1579	2.685 down
LMO1581	4.200 down
LMO1582	3.823 down
LMO1583	35.512 down
LMO1584	11.551 down
LMO1585	16.129 down
LMO1586	7.982 down
LMO1592	4.830 down
LMO1593	6.199 down
LMO1594	6.342 down
LMO1595	5.976 down
LMO1596	5.605 down
LMO1597	4.247 down
LMO1598	4.763 down
LMO1599	7.448 down
LMO1600	15.114 down
LMO1601	2.250 down
LMO1603	5.886 down
LMO1604	7.924 down
LMO1605	4.854 down
LMO1606	6.830 down
LMO1607	4.928 down
LMO1608	2.542 down
LMO1609	2.774 down
LMO1610	2.540 down
LMO1611	2.235 down
LMO1612	2.979 down
LMO1613	5.944 down
LMO1614	12.676 down
LMO1615	63.822 down
LMO1616	14.713 down
LMO1617	7.909 down
LMO1618	8.322 down
LMO1619	3.372 down
LMO1620	2.229 down
LMO1621	5.532 down
LMO1622	11.229 down
LMO1623	10.341 down
LMO1624	4.003 down
LMO1625	4.804 down

LMO1626	6.999 down
LMO1627	4.717 down
LMO1629	3.867 down
LMO1630	2.656 down
LMO1631	2.675 down
LMO1633	2.911 down
LMO1635	24.150 down
LMO1636	4.736 down
LMO1638	2.377 down
LMO1639	14.660 down
LMO1640	6.084 down
LMO1641	7.733 down
LMO1642	7.553 down
LMO1643	7.371 down
LMO1644	5.785 down
LMO1645	4.675 down
LMO1646	13.151 down
LMO1647	12.443 down
LMO1648	11.596 down
LMO1649	4.108 down
LMO1652	2.067 down
LMO1653	7.970 down
LMO1654	14.133 down
LMO1655	2.615 down
LMO1656	9.322 down
LMO1657	4.335 down
LMO1658	3.149 down
LMO1659	3.208 down
LMO1660	5.857 down
LMO1661	2.267 down
LMO1662	4.241 down
LMO1664	20.770 down
LMO1665	19.634 down
LMO1667	26.768 down
LMO1669	12.110 down
LMO1671	22.065 down
LMO1672	2.358 down
LMO1673	3.891 down
LMO1674	2.607 down
LMO1675	4.273 down
LMO1676	4.604 down
LMO1677	16.675 down
LMO1678	6.428 down
LMO1679	2.251 down
LMO1680	3.905 down
LMO1681	5.082 down
LMO1682	5.256 down
LMO1684	2.339 down

LMO1685	3.522 down
LMO1686	3.823 down
LMO1687	7.556 down
LMO1688	9.105 down
LMO1689	13.679 down
LMO1691	3.031 down
LMO1692	5.857 down
LMO1693	8.277 down
LMO1695	5.763 down
LMO1696	21.415 down
LMO1697	3.830 down
LMO1698	6.719 down
LMO1699	2.183 down
LMO1700	4.032 down
LMO1701	4.628 down
LMO1702	5.148 down
LMO1703	5.722 down
LMO1704	2.546 down
LMO1705	5.317 down
LMO1706	7.264 down
LMO1707	7.276 down
LMO1708	5.637 down
LMO1709	4.791 down
LMO1710	14.133 down
LMO1711	5.026 down
LMO1712	5.026 down
LMO1713	11.250 down
LMO1715	3.484 down
LMO1716	5.478 down
LMO1717	5.218 down
LMO1718	3.359 up
LMO1719	3.187 up
LMO1720	2.066 up
LMO1721	5.326 down
LMO1722	13.629 down
LMO1723	19.235 down
LMO1724	23.030 down
LMO1725	13.835 down
LMO1726	2.960 down
LMO1727	3.310 down
LMO1728	2.030 down
LMO1730	2.573 up
LMO1732	8.345 down
LMO1733	2.995 down
LMO1734	4.560 down
LMO1735	16.047 down
LMO1736	43.469 down
LMO1737	7.464 down

LMO1738	37.999 down
LMO1739	36.933 down
LMO1740	41.023 down
LMO1741	3.201 down
LMO1742	5.167 down
LMO1743	5.799 down
LMO1744	14.681 down
LMO1745	4.972 down
LMO1746	6.635 down
LMO1747	4.367 down
LMO1748	15.679 down
LMO1749	46.524 down
LMO1750	8.973 down
LMO1751	5.074 down
LMO1752	7.923 down
LMO1753	6.299 down
LMO1754	6.120 down
LMO1755	5.755 down
LMO1756	3.556 down
LMO1757	2.243 down
LMO1760	4.657 down
LMO1761	12.131 down
LMO1762	11.885 down
LMO1763	4.936 down
LMO1765	3.482 down
LMO1766	3.399 down
LMO1767	2.556 down
LMO1770	8.339 down
LMO1771	7.906 down
LMO1772	4.949 down
LMO1773	4.068 down
LMO1774	2.490 down
LMO1775	13.106 down
LMO1776	10.284 down
LMO1777	9.457 down
LMO1778	8.677 down
LMO1779	17.077 down
LMO1780	2.562 down
LMO1781	6.067 down
LMO1782	13.880 down
LMO1783	4.975 down
LMO1784	2.625 down
LMO1785	2.363 down
LMO1786	3.603 down
LMO1788	2.985 down
LMO1789	2.103 down
LMO1793	5.371 down
LMO1794	7.691 down

LMO1795	4.179 down
LMO1796	4.704 down
LMO1797	5.820 down
LMO1798	3.233 down
LMO1799	3.006 down
LMO1801	3.676 down
LMO1802	8.276 down
LMO1803	4.097 down
LMO1804	2.918 down
LMO1805	11.539 down
LMO1806	4.054 down
LMO1807	8.294 down
LMO1808	6.883 down
LMO1809	17.388 down
LMO1810	17.393 down
LMO1811	12.683 down
LMO1812	6.911 down
LMO1813	4.273 down
LMO1814	5.081 down
LMO1815	16.726 down
LMO1817	6.673 down
LMO1818	10.596 down
LMO1819	3.581 down
LMO1820	3.680 down
LMO1821	3.647 down
LMO1822	2.373 down
LMO1823	8.520 down
LMO1824	2.301 down
LMO1825	4.740 down
LMO1826	10.656 down
LMO1827	6.503 down
LMO1828	10.653 down
LMO1829	2.958 down
LMO1835	2.848 down
LMO1838	3.718 down
LMO1839	2.553 down
LMO1840	6.091 down
LMO1841	14.665 down
LMO1842	5.957 down
LMO1843	2.729 down
LMO1844	5.330 down
LMO1845	2.926 down
LMO1846	2.588 down
LMO1847	4.733 down
LMO1848	2.933 down
LMO1849	2.237 down
LMO1850	7.312 down
LMO1851	9.071 down

LMO1852	3.679 down
LMO1853	3.292 down
LMO1854	5.851 down
LMO1855	3.636 down
LMO1856	3.558 down
LMO1857	3.479 down
LMO1858	3.183 down
LMO1859	2.162 down
LMO1861	3.575 down
LMO1862	3.052 down
LMO1863	4.431 down
LMO1864	27.422 down
LMO1865	16.234 down
LMO1866	11.662 down
LMO1867	4.066 down
LMO1868	4.553 down
LMO1869	9.463 down
LMO1870	36.108 down
LMO1871	9.571 down
LMO1872	8.012 down
LMO1873	3.970 down
LMO1874	3.349 down
LMO1875	5.928 down
LMO1876	6.439 down
LMO1877	2.168 down
LMO1878	14.482 down
LMO1880	16.149 down
LMO1881	3.453 down
LMO1882	3.178 down
LMO1883	6.959 up
LMO1884	7.956 down
LMO1885	2.298 down
LMO1886	7.740 down
LMO1887	10.909 down
LMO1888	3.175 down
LMO1889	6.889 down
LMO1890	11.286 down
LMO1891	9.310 down
LMO1892	6.083 down
LMO1893	3.266 down
LMO1894	3.864 down
LMO1895	2.978 down
LMO1896	4.921 down
LMO1897	3.732 down
LMO1898	4.899 down
LMO1899	4.900 down
LMO1900	4.908 down
LMO1901	2.609 down

LMO1902	7.741 down
LMO1903	77.354 down
LMO1904	5.225 down
LMO1906	6.296 down
LMO1907	5.864 down
LMO1909	8.784 down
LMO1910	52.433 down
LMO1911	49.539 down
LMO1912	7.473 down
LMO1913	3.257 down
LMO1914	6.107 down
LMO1915	18.248 down
LMO1916	7.139 down
LMO1917	2.052 down
LMO1918	10.933 down
LMO1919	7.136 down
LMO1920	9.561 down
LMO1921	6.070 down
LMO1923	4.109 down
LMO1924	3.871 down
LMO1925	8.376 down
LMO1926	21.257 down
LMO1927	4.676 down
LMO1928	4.783 down
LMO1929	6.836 down
LMO1930	20.285 down
LMO1931	6.702 down
LMO1932	12.698 down
LMO1933	11.564 down
LMO1934	3.194 down
LMO1935	10.574 down
LMO1936	4.945 down
LMO1937	5.792 down
LMO1938	3.185 down
LMO1939	5.851 down
LMO1940	6.786 down
LMO1941	7.361 down
LMO1942	3.545 down
LMO1943	2.488 down
LMO1944	6.301 down
LMO1946	8.241 down
LMO1947	7.686 down
LMO1948	3.762 down
LMO1949	5.960 down
LMO1950	6.308 down
LMO1951	7.757 down
LMO1952	5.432 down
LMO1955	2.629 down

LMO1956	2.729 down
LMO1957	15.623 down
LMO1958	10.826 down
LMO1959	9.249 down
LMO1960	7.457 down
LMO1961	4.478 down
LMO1963	2.109 down
LMO1965	10.839 down
LMO1968	2.253 down
LMO1969	2.886 down
LMO1970	2.924 down
LMO1971	5.141 down
LMO1972	2.392 down
LMO1973	3.562 down
LMO1974	5.697 down
LMO1976	4.505 down
LMO1977	25.796 down
LMO1978	13.176 down
LMO1979	8.251 down
LMO1980	7.422 down
LMO1981	9.935 down
LMO1982	34.425 down
LMO1983	4.276 down
LMO1993	3.214 down
LMO1994	18.969 down
LMO1995	3.623 down
LMO1996	8.918 down
LMO2000	5.273 up
LMO2002	3.140 up
LMO2005	3.789 down
LMO2007	2.896 down
LMO2008	7.836 down
LMO2009	12.166 down
LMO2010	8.330 down
LMO2011	4.569 down
LMO2012	6.193 down
LMO2013	3.268 down
LMO2014	2.100 down
LMO2015	16.274 down
LMO2016	3.527 down
LMO2017	6.674 down
LMO2018	4.638 down
LMO2019	4.605 down
LMO2020	6.129 down
LMO2021	15.260 down
LMO2023	2.693 down
LMO2028	7.269 down
LMO2029	3.514 down

LMO2030	3.781 down
LMO2031	2.539 down
LMO2032	3.015 down
LMO2033	6.201 down
LMO2034	6.492 down
LMO2035	7.081 down
LMO2036	2.154 down
LMO2037	7.267 down
LMO2038	10.718 down
LMO2039	2.619 down
LMO2040	66.299 down
LMO2041	18.367 down
LMO2042	24.103 down
LMO2043	3.319 down
LMO2044	9.320 down
LMO2045	13.642 down
LMO2046	11.264 down
LMO2047	4.083 down
LMO2048	11.670 down
LMO2049	3.156 down
LMO2050	2.622 up
LMO2051	11.361 down
LMO2052	10.457 down
LMO2053	12.754 down
LMO2054	5.634 down
LMO2055	2.734 down
LMO2056	7.950 down
LMO2057	5.138 down
LMO2058	24.972 down
LMO2059	6.969 down
LMO2060	11.962 down
LMO2061	10.158 down
LMO2062	29.505 down
LMO2063	9.136 down
LMO2064	6.010 down
LMO2067	2.048 down
LMO2070	7.941 down
LMO2071	6.743 down
LMO2072	3.649 down
LMO2073	13.491 down
LMO2074	10.914 down
LMO2075	3.606 down
LMO2076	4.154 down
LMO2077	4.855 down
LMO2078	6.536 down
LMO2079	25.732 down
LMO2080	9.367 down
LMO2081	5.149 down

LMO2082	7.246 down
LMO2083	4.093 down
LMO2084	3.587 down
LMO2086	7.088 down
LMO2087	5.072 down
LMO2088	5.049 down
LMO2090	2.002 up
LMO2092	6.269 down
LMO2094	4.054 down
LMO2095	2.541 down
LMO2096	15.552 down
LMO2097	4.027 down
LMO2098	2.507 down
LMO2100	14.901 down
LMO2102	4.000 down
LMO2103	5.135 down
LMO2104	10.061 down
LMO2105	17.012 down
LMO2106	6.911 down
LMO2107	6.995 down
LMO2108	3.945 down
LMO2109	9.914 down
LMO2110	3.376 down
LMO2111	9.799 down
LMO2112	11.782 down
LMO2113	6.078 down
LMO2114	5.238 down
LMO2115	7.275 down
LMO2116	74.067 down
LMO2117	6.219 down
LMO2118	7.937 down
LMO2119	3.139 down
LMO2120	2.830 down
LMO2121	5.729 up
LMO2122	9.095 up
LMO2123	5.043 up
LMO2125	8.268 up
LMO2127	11.794 down
LMO2128	6.377 down
LMO2129	8.502 down
LMO2130	2.076 down
LMO2131	4.258 down
LMO2133	4.509 down
LMO2139	4.580 down
LMO2140	5.039 down
LMO2141	4.331 down
LMO2142	3.320 down
LMO2143	7.915 down

LMO2144	7.520 down
LMO2145	2.184 down
LMO2148	3.428 down
LMO2149	2.587 down
LMO2155	2.000 up
LMO2156	20.629 down
LMO2163	2.320 up
LMO2164	9.537 down
LMO2165	4.218 down
LMO2166	12.117 down
LMO2167	7.248 down
LMO2168	4.683 down
LMO2169	5.234 down
LMO2172	2.425 up
LMO2173	3.103 down
LMO2174	8.287 down
LMO2175	3.096 up
LMO2176	9.060 down
LMO2177	5.232 down
LMO2178	5.607 down
LMO2179	6.506 down
LMO2181	2.536 down
LMO2184	2.852 down
LMO2187	5.187 down
LMO2188	3.848 down
LMO2189	3.909 down
LMO2191	3.511 down
LMO2192	3.443 down
LMO2193	2.978 down
LMO2194	3.381 down
LMO2195	3.816 down
LMO2196	2.026 down
LMO2197	12.462 down
LMO2198	3.217 down
LMO2200	4.695 down
LMO2201	2.994 down
LMO2202	6.465 down
LMO2203	4.164 down
LMO2204	2.012 down
LMO2207	9.128 down
LMO2208	9.220 down
LMO2209	8.714 down
LMO2211	8.799 down
LMO2212	7.544 down
LMO2214	6.789 down
LMO2215	5.294 down
LMO2216	4.072 down
LMO2217	5.005 down

LMO2218	13.435 down
LMO2219	4.191 down
LMO2221	2.043 down
LMO2222	2.417 down
LMO2223	9.006 down
LMO2224	5.637 down
LMO2225	8.723 down
LMO2226	4.678 down
LMO2227	3.055 down
LMO2229	6.904 down
LMO2231	2.740 down
LMO2232	2.571 down
LMO2233	8.105 down
LMO2234	2.046 down
LMO2236	6.161 down
LMO2237	3.745 down
LMO2238	4.032 down
LMO2239	19.657 down
LMO2240	29.306 down
LMO2241	55.059 down
LMO2242	4.515 down
LMO2243	9.681 down
LMO2244	6.672 down
LMO2245	5.069 down
LMO2246	4.557 down
LMO2247	3.754 down
LMO2248	4.550 down
LMO2249	2.503 down
LMO2250	3.081 down
LMO2252	19.473 down
LMO2253	5.846 down
LMO2254	14.388 down
LMO2255	5.208 down
LMO2257	7.581 up
LMO2259	19.115 down
LMO2260	7.092 down
LMO2261	4.407 down
LMO2262	7.118 down
LMO2263	4.023 down
LMO2264	2.035 down
LMO2265	2.210 down
LMO2267	2.196 down
LMO2277	3.877 down
LMO2278	2.354 up
LMO2279	3.079 up
LMO2280	2.493 up
LMO2281	3.109 up
LMO2282	3.159 up

LMO2283	2.322	up
LMO2284	3.364	up
LMO2285	3.541	up
LMO2286	3.804	up
LMO2287	3.466	up
LMO2288	3.980	up
LMO2289	2.785	up
LMO2290	2.748	up
LMO2291	2.414	up
LMO2292	2.853	up
LMO2293	3.216	up
LMO2294	3.404	up
LMO2295	2.270	up
LMO2296	2.127	up
LMO2297	2.489	up
LMO2298	2.943	up
LMO2299	4.314	up
LMO2300	3.554	up
LMO2301	2.015	up
LMO2303	2.275	up
LMO2306	2.540	up
LMO2307	2.065	up
LMO2309	2.140	up
LMO2311	2.625	up
LMO2313	2.142	up
LMO2314	3.404	up
LMO2315	2.089	up
LMO2316	2.224	up
LMO2317	2.071	up
LMO2319	2.195	up
LMO2322	2.287	up
LMO2326	3.801	up
LMO2327	3.507	up
LMO2329	9.300	down
LMO2330	2.272	down
LMO2334	47.450	down
LMO2335	2.738	down
LMO2337	3.447	down
LMO2339	2.885	down
LMO2342	4.614	down
LMO2343	3.375	down
LMO2344	4.442	down
LMO2345	4.899	down
LMO2346	4.752	down
LMO2347	7.246	down
LMO2348	7.182	down
LMO2349	7.918	down
LMO2350	7.627	down

LMO2351	14.477 down
LMO2352	45.074 down
LMO2353	3.902 down
LMO2354	4.337 down
LMO2355	4.910 down
LMO2358	2.522 down
LMO2359	4.038 down
LMO2360	3.297 down
LMO2361	5.701 down
LMO2362	2.267 down
LMO2363	2.586 down
LMO2364	2.526 down
LMO2365	3.616 down
LMO2366	10.625 down
LMO2368	3.932 down
LMO2369	5.827 down
LMO2371	7.968 down
LMO2372	5.919 down
LMO2374	10.618 down
LMO2375	9.614 down
LMO2376	3.868 down
LMO2377	14.377 down
LMO2378	7.210 down
LMO2379	7.110 down
LMO2380	5.073 down
LMO2381	5.551 down
LMO2382	2.936 down
LMO2383	3.702 down
LMO2384	4.880 down
LMO2385	3.898 down
LMO2386	4.031 down
LMO2387	3.014 down
LMO2388	5.147 down
LMO2389	2.111 down
LMO2390	3.057 down
LMO2392	5.588 down
LMO2393	3.716 down
LMO2395	7.708 down
LMO2396	3.611 down
LMO2397	13.294 down
LMO2399	4.088 down
LMO2400	6.192 down
LMO2401	3.350 down
LMO2402	3.189 down
LMO2403	4.961 down
LMO2404	3.031 down
LMO2405	4.254 down
LMO2407	3.824 down

LMO2408	16.924	down
LMO2409	42.966	down
LMO2410	6.851	down
LMO2411	6.136	down
LMO2416	16.540	down
LMO2417	8.764	down
LMO2418	9.693	down
LMO2419	5.625	down
LMO2420	4.253	down
LMO2421	3.049	down
LMO2422	3.966	down
LMO2423	7.055	down
LMO2424	9.364	down
LMO2425	3.855	down
LMO2427	5.308	down
LMO2428	11.287	down
LMO2429	3.391	down
LMO2430	2.803	down
LMO2431	4.581	down
LMO2433	8.290	down
LMO2435	10.246	down
LMO2436	2.254	down
LMO2437	2.236	down
LMO2438	4.064	down
LMO2439	8.006	down
LMO2440	4.484	down
LMO2441	8.881	down
LMO2442	6.311	down
LMO2443	7.960	down
LMO2444	3.203	down
LMO2447	2.077	down
LMO2448	3.538	down
LMO2450	3.847	down
LMO2451	4.586	down
LMO2452	2.162	down
LMO2454	5.194	up
LMO2460	3.887	down
LMO2461	4.193	down
LMO2462	2.048	down
LMO2463	3.712	down
LMO2464	18.164	down
LMO2465	12.400	down
LMO2466	16.876	down
LMO2467	5.974	down
LMO2468	2.381	down
LMO2469	6.557	down
LMO2472	3.351	down
LMO2473	3.454	down

LMO2474	2.842 down
LMO2475	7.829 down
LMO2476	4.580 down
LMO2477	6.185 down
LMO2478	5.839 down
LMO2479	2.128 down
LMO2480	5.026 down
LMO2481	2.501 down
LMO2482	5.970 down
LMO2483	4.173 down
LMO2487	2.409 up
LMO2488	2.071 down
LMO2490	4.158 down
LMO2491	6.000 down
LMO2492	5.745 down
LMO2493	9.130 down
LMO2494	2.912 down
LMO2495	2.739 down
LMO2496	2.622 down
LMO2497	2.647 down
LMO2498	3.642 down
LMO2499	3.317 down
LMO2500	11.859 down
LMO2501	7.286 down
LMO2502	8.629 down
LMO2503	4.800 down
LMO2504	14.040 down
LMO2505	10.045 down
LMO2506	5.700 down
LMO2507	4.567 down
LMO2508	2.489 down
LMO2509	5.126 down
LMO2510	3.748 down
LMO2512	9.738 down
LMO2513	7.393 down
LMO2514	3.121 down
LMO2515	2.646 down
LMO2516	12.981 down
LMO2517	9.892 down
LMO2518	6.724 down
LMO2519	6.309 down
LMO2520	6.040 down
LMO2521	12.340 down
LMO2522	4.657 down
LMO2524	4.866 down
LMO2525	4.941 down
LMO2526	7.128 down
LMO2527	9.734 down

LMO2535	2.581 down
LMO2536	3.811 down
LMO2537	11.643 down
LMO2538	2.688 down
LMO2539	2.559 down
LMO2540	5.863 down
LMO2541	3.243 down
LMO2542	2.997 down
LMO2543	4.262 down
LMO2544	3.763 down
LMO2545	11.685 down
LMO2546	6.006 down
LMO2547	21.543 down
LMO2548	2.241 down
LMO2549	9.284 down
LMO2550	6.952 down
LMO2551	6.829 down
LMO2552	5.416 down
LMO2553	4.530 down
LMO2554	4.995 down
LMO2555	2.744 down
LMO2557	9.205 down
LMO2558	5.723 down
LMO2559	7.896 down
LMO2560	3.257 down
LMO2561	4.624 down
LMO2562	11.772 down
LMO2563	34.952 down
LMO2565	4.658 down
LMO2566	6.731 down
LMO2567	4.788 up
LMO2569	8.215 down
LMO2570	2.515 down
LMO2575	3.179 down
LMO2577	7.356 down
LMO2578	3.848 down
LMO2579	4.617 down
LMO2580	5.660 down
LMO2581	3.398 down
LMO2582	6.089 down
LMO2583	3.497 down
LMO2585	5.771 up
LMO2586	6.222 up
LMO2587	18.147 down
LMO2590	2.637 down
LMO2591	2.790 down
LMO2594	3.032 down
LMO2595	3.918 down

LMO2596	5.217 down
LMO2598	3.073 down
LMO2599	3.696 down
LMO2600	3.454 down
LMO2601	4.679 down
LMO2603	2.805 down
LMO2604	3.252 down
LMO2605	3.303 down
LMO2610	5.366 down
LMO2634	3.318 down
LMO2635	3.143 down
LMO2636	3.039 down
LMO2637	4.575 down
LMO2639	4.455 down
LMO2640	3.753 down
LMO2641	3.088 down
LMO2642	2.126 down
LMO2643	7.569 down
LMO2644	5.146 down
LMO2645	98.664 up
LMO2646	48.757 up
LMO2647	50.049 up
LMO2648	50.803 up
LMO2649	28.152 up
LMO2650	13.512 up
LMO2651	13.487 up
LMO2654	2.203 up
LMO2658	5.345 down
LMO2659	2.349 up
LMO2660	3.527 up
LMO2661	8.397 up
LMO2662	11.375 up
LMO2663	16.199 up
LMO2664	9.926 up
LMO2665	9.916 up
LMO2666	7.420 up
LMO2667	5.991 up
LMO2668	5.401 up
LMO2669	2.528 down
LMO2673	2.315 up
LMO2674	2.134 up
LMO2676	2.144 up
LMO2681	2.287 up
LMO2683	5.156 up
LMO2684	5.738 up
LMO2685	8.777 up
LMO2686	13.586 down
LMO2687	8.929 down

LMO2688	8.828 down
LMO2689	11.286 down
LMO2690	69.169 down
LMO2692	2.815 down
LMO2693	2.053 down
LMO2694	3.428 down
LMO2696	2.062 up
LMO2698	2.340 down
LMO2700	2.283 down
LMO2701	2.462 down
LMO2702	2.146 down
LMO2703	3.572 down
LMO2704	2.027 down
LMO2705	2.896 down
LMO2707	2.698 down
LMO2708	11.343 up
LMO2710	11.428 down
LMO2712	2.042 down
LMO2714	2.063 up
LMO2715	2.509 down
LMO2716	2.202 down
LMO2717	2.161 down
LMO2719	9.930 down
LMO2720	32.501 down
LMO2721	2.622 down
LMO2722	3.922 down
LMO2723	4.472 down
LMO2725	3.485 down
LMO2726	9.110 down
LMO2727	4.416 down
LMO2728	4.860 down
LMO2729	5.227 down
LMO2732	2.550 down
LMO2734	5.075 up
LMO2735	2.931 up
LMO2737	15.470 down
LMO2738	6.382 down
LMO2741	2.408 down
LMO2742	2.095 up
LMO2744	9.723 down
LMO2745	2.083 down
LMO2746	3.950 down
LMO2747	6.898 down
LMO2749	18.432 down
LMO2750	6.550 down
LMO2753	13.547 down
LMO2754	13.990 down
LMO2755	3.099 down

LMO2756	7.856 down
LMO2757	3.688 down
LMO2759	3.028 down
LMO2760	3.365 down
LMO2761	11.619 down
LMO2762	5.415 down
LMO2763	3.653 down
LMO2764	3.159 down
LMO2765	2.400 down
LMO2766	13.240 down
LMO2767	7.310 down
LMO2768	52.959 down
LMO2769	16.548 down
LMO2770	5.747 down
LMO2773	2.665 down
LMO2774	4.123 down
LMO2775	2.098 down
LMO2777	8.584 down
LMO2779	7.360 down
LMO2781	9.731 up
LMO2782	7.112 up
LMO2783	3.871 up
LMO2784	3.587 down
LMO2785	3.124 down
LMO2786	6.607 down
LMO2787	7.947 down
LMO2788	5.211 down
LMO2789	8.531 down
LMO2790	5.573 down
LMO2791	8.196 down
LMO2793	46.304 down
LMO2794	7.076 down
LMO2795	6.817 down
LMO2797	2.233 down
LMO2799	4.085 up
LMO2801	3.084 up
LMO2802	7.367 down
LMO2803	13.163 down
LMO2804	5.132 down
LMO2805	7.071 down
LMO2806	3.873 down
LMO2810	17.823 down
LMO2811	10.200 down
LMO2814	5.188 down
LMO2815	2.469 down
LMO2816	2.895 up
LMO2818	2.100 up
LMO2820	8.452 down

LMO2821	53.309 down
LMO2822	3.519 down
LMO2823	5.359 down
LMO2825	4.271 down
LMO2826	7.775 down
LMO2827	6.726 down
LMO2829	9.362 down
LMO2830	6.849 down
LMO2831	7.248 down
LMO2832	3.963 down
LMO2833	5.556 down
LMO2834	2.012 down
LMO2835	2.315 down
LMO2839	2.904 down
LMO2841	5.786 down
LMO2842	10.361 down
LMO2843	14.173 down
LMO2844	12.083 down
LMO2845	7.944 down
LMO2846	2.508 up
LMO2849	3.926 up
LMO2850	2.694 up
LMO2852	7.536 down
LMO2853	5.491 down
LMO2854	8.342 down
LMO2855	10.463 down
LMO2856	33.757 down
