# Supplementary material for: Changes in Gene Expression during Adaptation of Listeria monocytogenes to the Soil Environment
Source: PLoS One. 2011 Sep 23;6(9):e24881. doi: 10.1371/journal.pone.0024881 (PMC3179493; doi:10.1371/journal.pone.0024881)
Supplement: Table S4 — Gene ontology «functional categories » at time 15 minutes. (PDF) [file pone.0024881.s004.pdf]

Table S4. Gene ontology « functional categories » 15 minutes

gene name	description	category	fold change
lmo2818	similar to transmembrane efflux protein	1.2	5.167 up
lmo2171	similar to antiporter proteins	1.2	5.184 up
lmo2850	similar to sugar transport proteins	1.2	16.839 up
lmo2816	similar to transport protein	1.2	15.953 up
lmo2799	similar to phosphotransferase system mannitol-specific enzyme IIBC	1.2	3.864 up
lmo2783	similar to cellobiose phosphotransferase system enzyme IIC	1.2	18.995 up
lmo2782	similar to PTS. cellobiose-specific IIB component	1.2	27.191 up
lmo2780	similar to cellobiose PTS enzyme IIA	1.2	3.774 up
lmo2772	similar to beta-glucoside-specific enzyme IIABC	1.2	4.708 up
lmo2733	similar to PTS system. fructose-specific IIABC component	1.2	4.733 up
lmo2708	similar to PTS system. cellobiose-specific enzyme IIC	1.2	20.466 up
lmo2685	similar to cellobiose phosphotransferase enzyme IIA component	1.2	11.418 up
lmo2684	similar to cellobiose phosphotransferase enzyme IIC component	1.2	8.427 up
lmo2683	similar to cellobiose phosphotransferase enzyme IIB component	1.2	8.343 up
lmo2666	similar to PTS system galactitol-specific enzyme IIB component	1.2	15.747 up
lmo2665	similar to PTS system galactitol-specific enzyme IIC component	1.2	14.893 up
lmo2650	similar to hypothetical PTS enzyme IIB component	1.2	26.143 up
lmo2136	similar to PTS system. fructose-specific enzyme IIB component	1.2	5.115 up
lmo2135	similar to PTS system. fructose-specific enzyme IIC component	1.2	2.749 up
lmo2125	similar to maltose/maltodextrin ABC-transporter (binding protein)	1.2	12.023 up
lmo2124	similar to maltodextrin ABC-transport system (permease)	1.2	10.151 up
lmo2123	similar to maltodextrin ABC-transport system (permease)	1.2	11.361 up
lmo2002	similar to PTS mannose-specific enzyme IIB component	1.2	8.015 up
lmo2001	similar to PTS mannose-specific enzyme IIC component	1.2	6.605 up
lmo2000	similar to PTS mannose-specific enzyme IID component	1.2	10.236 up
lmo1997	similar to PTS mannose-specific enzyme IIA component	1.2	5.834 up
lmo1732	similar to sugar ABC transporter. permease protein	1.2	2.885 up
lmo1731	similar to sugar ABC transporter. permease protein	1.2	5.217 up
lmo1730	similar to sugar ABC transporter binding protein	1.2	11.181 up
lmo1720	similar to phosphotransferase system (PTS) lichenan-specific enzyme IIB component	1.2	5.168 up
lmo1719	similar to phosphotransferase system (PTS) lichenan-specific enzyme IIA component	1.2	5.354 up
lmo0916	similar to phosphotransferase system enzyme IIA	1.2	35.521 up
lmo0915	similar to phosphotransferase system enzyme IIC	1.2	21.540 up
lmo0914	similar to PTS system. IIB component	1.2	23.186 up
lmo0876	similar to PTS system. Lichenan-specific enzyme IIC component	1.2	3.433 up
lmo0875	similar to PTS system. beta-glucoside enzyme IIB component	1.2	4.492 up
lmo0874	similar to PTS system enzyme IIA component	1.2	4.725 up
lmo0781	similar to mannose-specific phosphotransferase system (PTS) component IID	1.2	2.470 up
lmo0633	similar to PTS system. fructose-specific IIB component	1.2	3.209 up
lmo0632	similar to PTS system. fructose-specific IIC component	1.2	4.421 up
lmo0544	similar to PTS system. glucitol/sorbitol-specific enzyme II CII component	1.2	14.110 up
lmo0543	similar to PTS system. glucitol/sorbitol-specific enzyme IIBC component	1.2	3.289 up
lmo0400	similar to fructose-specific phosphotransferase enzyme IIC	1.2	6.875 up
lmo0399	similar to fructose-specific phosphotransferase enzyme IIB	1.2	3.859 up
lmo0301	similar to PTS beta-glucoside-specific enzyme IIA component	1.2	17.073 up
lmo0299	similar to PTS beta-glucoside-specific enzyme IIB component	1.2	13.398 up

lmo0298	similar to PTS beta-glucoside-specific enzyme IIC component	1.2	13.358 up
lmo0278	similar to sugar ABC transporter. ATP-binding protein	1.2	3.867 up
lmo0181	similar to sugar ABC transporter. sugar-binding protein	1.2	2.024 up
lmo0027	similar to PTS system. beta-glucosides specific enzyme IIABC	1.2	18.344 up
lmo0024	similar to PTS system. mannose-specific IID component	1.2	19.291 up
lmo0023	similar to PTS system. fructose-specific IIC component	1.2	15.139 up
lmo0022	similar to PTS system. fructose-specific IIB component	1.2	19.035 up
lmo0021	similar to PTS system. fructose-specific IIA component	1.2	11.202 up
lmo2667	similar to PTS system galactitol-specific enzyme IIA component	1.2	6.649 up
lmo2651	similar to mannitol-specific PTS enzyme IIA component	1.2	33.435 up
lmo2137	similar to PTS system. fructose-specific enzyme IIA component	1.2	5.052 up
lmo0631	similar to PTS system. fructose-specific IIA component	1.2	4.174 up
lmo0503	similar to PTS fructose-specific enzyme IIA component	1.2	2.766 up
lmo0398	similar to phosphotransferase system enzyme IIA	1.2	4.494 up
lmo0357	similar to PTS system. enzyme IIA component	1.2	2.209 up
kdpA	highly similar to potassium-transporting atpase a chain	1.2	3.003 up
kdpB	potassium-transporting atpase b chain	1.2	2.798 up
lmo2649	similar to hypothetical PTS enzyme IIC component	1.2	32.946 up
lmo0608	similar to ABC transporter. ATP-binding protein	1.2	2.661 up
lmo2584	similar to formate dehydrogenase associated protein	2.1.1	3.283 up
lmo1538	similar to glycerol kinase	2.1.1	3.467 up
glpD	similar to glycerol 3 phosphate dehydrogenase	2.1.1	2.898 up
lmo0878	similar to oxidoreductases	2.1.1	3.251 up
lmo0383	highly similar to B. subtilis methylmalonate-semialdehyde dehydrogenase IolA	2.1.1	7.233 up
lmo0386	similar to B. subtilis IolD protein. to acetolactate synthase	2.1.1	3.020 up
lmo2664	similar to sorbitol dehydrogenase	2.1.1	9.634 up
lmo2663	similar to polyol dehydrogenase	2.1.1	9.744 up
lmo2849	similar to rhamnulokinase	2.1.1	8.746 up
lmo2848	highly similar to L-rhamnose isomerase	2.1.1	7.039 up
lmo2847	highly similar to rhamnulose-1-phosphate aldolase	2.1.1	3.324 up
lmo2801	similar to a putative N-acetylmannosamine-6-phosphate epimerase	2.1.1	9.175 up
lmo2781	similar to beta-glucosidase	2.1.1	15.760 up
lmo2771	similar to beta-glucosidase	2.1.1	2.043 up
lmo2743	similar to transaldolase	2.1.1	4.336 up
lmo2735	similar to Sucrose phosphorylase	2.1.1	6.244 up
lmo2734	weakly similar to sugar hydrolase	2.1.1	6.192 up
lmo2674	similar to ribose 5-phosphate epimerase	2.1.1	3.332 up
lmo2662	similar to ribose 5-phosphate epimerase	2.1.1	8.669 up
lmo2661	similar to ribulose-5-phosphate 3-epimerase	2.1.1	8.132 up
lmo2126	similar to maltogenic amylase	2.1.1	2.538 up
lmo2121	similar to maltosephosphorylase	2.1.1	9.319 up
lmo1883	similar to chitinases	2.1.1	7.157 up
lmo1729	similar to beta-glucosidases	2.1.1	2.676 up
lmo1254	similar to alpha.alpha-phosphotrehalase	2.1.1	2.167 up
lmo0957	similar to glucosamine-6-Phoasphate isomerase (EC 5.3.1.10)	2.1.1	2.650 up
lmo0917	similar to beta-glucosidase	2.1.1	60.712 up
lmo0877	similar to B. subtilis NagB protein (glucosamine-6-phosphate isomerase)	2.1.1	2.685 up
lmo0643	similar to putative transaldolase	2.1.1	6.213 up

lmo0634	similar to an E. coli putative tagatose 6-phosphate kinase	2.1.1	3.111 up
lmo0536	similar to 6-phospho-beta-glucosidase	2.1.1	5.450 up
lmo0401	highly similar to E. col YbgG protein. a putative sugar hydrolase	2.1.1	19.291 up
lmo0385	similar to B. subtilis IolC protein and to fructokinase	2.1.1	3.668 up
lmo0384	similar to B. subtilis IolB protein	2.1.1	6.414 up
lmo0348	similar to dihydroxyacetone kinase	2.1.1	4.088 up
lmo0347	similar to dihydroxyacetone kinase	2.1.1	3.007 up
lmo0345	similar to sugar-phosphate isomerase	2.1.1	2.632 up
lmo0319	similar to phospho-beta-glucosidase	2.1.1	2.193 up
lmo0300	similar to phospho-beta-glucosidase and phospho-beta-galactosidase	2.1.1	11.617 up
lmo0105	highly similar to chitinase B	2.1.1	34.138 up
lmo2172	similar to propionate CoA-transferase	2.1.1	4.861 up
lmo1999	weakly similar to glucosamine-fructose-6-phosphate aminotransferase	2.1.1	7.159 up
lmo1998	similar to opine catabolism protein	2.1.1	4.561 up
lmo0502	similar to putative sugar-phosphate isomerase	2.1.1	4.322 up
lmo2175	similar to dehydrogenase	2.1.1	7.305 up
lmo0344	similar to dehydrogenase/reductase	2.1.1	2.317 up
lmo2800	similar to dehydrogenase	2.1.1	3.867 up
lmo2586	similar to formate dehydrogenase alpha chain	2.1.1	7.368 up
lmo0640	similar to oxidoreductase	2.1.1	2.295 up
lmo0025	similar to phosphoheptose isomerase	2.1.1	2.012 up
lmo2122	similar to maltodextrose utilization protein MalA	2.1.1	9.721 up
leuB	similar to 3-isopropylmalate dehydrogenase	2.2	3.317 up
argH	similar to argininosuccinate lyase	2.2	5.961 up
argG	similar to argininosuccinate synthase	2.2	4.707 up
ilvA	similar to threonine dehydratase	2.2	3.346 up
leuD	similar to 3-isopropylmalate dehydratase (small subunit)	2.2	3.662 up
leuC	similar to 3-isopropylmalate dehydratase (large subunit)	2.2	3.209 up
leuA	similar to 2-isopropylmalate synthase	2.2	4.040 up
ilvN	similar to acetolactate synthase (acetohydroxy-acid synthase) (small subunit)	2.2	3.951 up
argC	similar to N-acetylglutamate gamma-semialdehyde dehydrogenases	2.2	4.317 up
argJ	highly similar to ornithine acetyltransferase and amino-acid acetyltransferases	2.2	3.770 up
argB	highly similar to N-acetylglutamate 5-phosphotransferase	2.2	2.314 up
lmo1350	similar to glycine dehydrogenase (decarboxylating) subunit 2	2.2	3.829 up
lmo1349	similar to glycine dehydrogenase (decarboxylating) subunit 1	2.2	7.360 up
lmo1348	similar to aminomethyltransferase	2.2	4.328 up
lmo0265	similar to succinyldiaminopimelate desuccinylase	2.2	2.270 up
lmo0039	similar to carbamate kinase	2.2	2.448 up
ilvD	similar to dihydroxy-acid dehydratase	2.2	2.438 up
ilvC	similar to ketol-acid reductoisomerase (acetohydroxy-acid isomeroreductase)	2.2	4.822 up
ilvB	similar to acetolactate synthase (acetohydroxy-acid synthase) (large subunit)	2.2	4.107 up
lmo1992	similar to alpha-acetolactate decarboxylase	2.2	2.197 up
lmo2819	similar to carboxypeptidase	2.2	2.630 up
lmo2817	similar to peptidases	2.2	6.409 up
lmo2647	similar to creatinine amidohydrolase	2.2	53.486 up
lmo2742	hypothetical protein	5.2	9.154 up
lmo0471	hypothetical protein	5.2	2.647 up
lmo2158	similar to B. subtilis YwmG protein	5.2	2.627 up

lmo2603	hypothetical protein	5.2	2.421 up
lmo0038	conserved hypothetical protein	5.2	2.182 up
lmo1256	hypothetical protein	5.2	2.998 up
lmo2736	conserved hypothetical protein	5.2	2.065 up
lmo2162	similar to unknown proteins	5.2	5.404 up
lmo2161	hypothetical protein	5.2	3.610 up
lmo2160	similar to unknown proteins	5.2	2.970 up
lmo0879	hypothetical protein	5.2	3.296 up
lmo2511	similar to conserved hypothetical proteins like to B. subtilis YvyD protein	5.2	3.764 up
lmo0629	hypothetical protein	5.2	2.153 up
lmo1830	similar to conserved hypotheticl proteins	5.2	2.456 up
lmo2213	similar to unknown protein	5.2	2.514 up
lmo2170	similar to unknown proteins	5.2	2.132 up
lmo0869	hypothetical protein	5.2	3.084 up
lmo0584	conserved hypothetical membrane protein	5.2	2.929 up
lmo0323	similar to unknown proteins	5.2	3.948 up
lmo0134	similar to E. coli YjdJ protein	5.2	2.804 up
lmo2846	highly similar to B. subtilis YulD protein	5.2	2.508 up
lmo2724	similar to unknown proteins	5.2	2.409 up
lmo2670	conserved hypothetical protein	5.2	3.495 up
lmo2585	similar to B. subtilis YrhD protein	5.2	7.101 up
lmo2570	hypothetical protein	5.2	2.112 up
lmo2487	similar to B. subtilis YvlB protein	5.2	2.307 up
lmo2357	similar to unknown protein	5.2	2.564 up
lmo1718	similar to putative outer surface protein	5.2	4.872 up
lmo1241	hypothetical protein	5.2	2.366 up
lmo1140	hypothetical protein	5.2	2.466 up
lmo0870	hypothetical protein	5.2	2.569 up
lmo0796	conserved hypothetical protein	5.2	2.209 up
lmo0496	similar to B. subtilis YnzC protein	5.2	2.259 up
lmo0351	similar to unknown proteins	5.2	7.184 up
lmo0133	similar to E. coli YjdI protein	5.2	2.363 up
lmo0720	hypothetical protein	5.2	2.378 up
lmo2673	conserved hypothetical protein	5.2	2.096 up
lmo1580	similar to unknown protein	5.2	2.291 up
lmo0515	conserved hypothetical protein	5.2	2.990 up
lmo2645	hypothetical protein	5.2	40.633 up
