# Supplementary material for: Changes in Gene Expression during Adaptation of Listeria monocytogenes to the Soil Environment
Source: PLoS One. 2011 Sep 23;6(9):e24881. doi: 10.1371/journal.pone.0024881 (PMC3179493; doi:10.1371/journal.pone.0024881)
Supplement: Table S5 — Gene ontology «functional categories » at time 30 minutes. (PDF) [file pone.0024881.s005.pdf]

Table S5. Gene ontology « functional category » 30 minutes

gene name	description	category	fold change
lmo2171	similar to antiporter proteins	1.2	4.328 up
lmo2818	similar to transmembrane efflux protein	1.2	4.878 up
lmo0021	similar to PTS system. fructose-specific IIA component	1.2	12.812 up
lmo0022	similar to PTS system. fructose-specific IIB component	1.2	19.697 up
lmo0023	similar to PTS system. fructose-specific IIC component	1.2	14.374 up
lmo0024	similar to PTS system. mannose-specific IID component	1.2	25.296 up
lmo0027	similar to PTS system. beta-glucosides specific enzyme IIABC	1.2	41.540 up
lmo0181	similar to sugar ABC transporter. sugar-binding protein	1.2	2.426 up
lmo0298	similar to PTS beta-glucoside-specific enzyme IIC component	1.2	15.822 up
lmo0299	similar to PTS beta-glucoside-specific enzyme IIB component	1.2	17.041 up
lmo0301	similar to PTS beta-glucoside-specific enzyme IIA component	1.2	12.663 up
lmo0399	similar to fructose-specific phosphotransferase enzyme IIB	1.2	3.478 up
lmo0400	similar to fructose-specific phosphotransferase enzyme IIC	1.2	7.580 up
lmo0544	similar to PTS system. glucitol/sorbitol-specific enzyme II CII component	1.2	7.390 up
lmo0632	similar to PTS system. fructose-specific IIC component	1.2	4.672 up
lmo0633	similar to PTS system. fructose-specific IIB component	1.2	4.049 up
lmo0781	similar to mannose-specific phosphotransferase system (PTS) component IID	1.2	2.357 up
lmo0874	similar to PTS system enzyme IIA component	1.2	6.369 up
lmo0875	similar to PTS system. beta-glucoside enzyme IIB component	1.2	3.987 up
lmo0876	similar to PTS system. Lichenan-specific enzyme IIC component	1.2	3.381 up
lmo0914	similar to PTS system. IIB component	1.2	21.601 up
lmo0915	similar to phosphotransferase system enzyme IIC	1.2	25.705 up
lmo0916	similar to phosphotransferase system enzyme IIA	1.2	49.999 up
lmo1719	similar to phosphotransferase system (PTS) lichenan-specific enzyme IIA component	1.2	4.219 up
lmo1720	similar to phosphotransferase system (PTS) lichenan-specific enzyme IIB component	1.2	3.472 up
lmo1730	similar to sugar ABC transporter binding protein	1.2	12.135 up
lmo1731	similar to sugar ABC transporter. permease protein	1.2	6.124 up
lmo1732	similar to sugar ABC transporter. permease protein	1.2	4.314 up
lmo1997	similar to PTS mannose-specific enzyme IIA component	1.2	6.082 up
lmo2000	similar to PTS mannose-specific enzyme IID component	1.2	13.563 up
lmo2001	similar to PTS mannose-specific enzyme IIC component	1.2	7.748 up
lmo2002	similar to PTS mannose-specific enzyme IIB component	1.2	7.982 up
lmo2123	similar to maltodextrin ABC-transport system (permease)	1.2	17.192 up
lmo2124	similar to maltodextrin ABC-transport system (permease)	1.2	6.687 up
lmo2125	similar to maltose/maltodextrin ABC-transporter (binding protein)	1.2	14.136 up
lmo2135	similar to PTS system. fructose-specific enzyme IIC component	1.2	3.002 up
lmo2136	similar to PTS system. fructose-specific enzyme IIB component	1.2	4.674 up
lmo2650	similar to hypothetical PTS enzyme IIB component	1.2	29.323 up
lmo2665	similar to PTS system galactitol-specific enzyme IIC component	1.2	20.652 up
lmo2666	similar to PTS system galactitol-specific enzyme IIB component	1.2	17.375 up
lmo2683	similar to cellobiose phosphotransferase enzyme IIB component	1.2	5.149 up
lmo2684	similar to cellobiose phosphotransferase enzyme IIC component	1.2	7.871 up
lmo2685	similar to cellobiose phosphotransferase enzyme IIA component	1.2	11.108 up
lmo2708	similar to PTS system. cellobiose-specific enzyme IIC	1.2	9.915 up
lmo2733	similar to PTS system. fructose-specific IIABC component	1.2	4.004 up

lmo2772	similar to beta-glucoside-specific enzyme IIABC	1.2	4.320 up
lmo2780	similar to cellobiose PTS enzyme IIA	1.2	3.593 up
lmo2782	similar to PTS. cellobiose-specific IIB component	1.2	31.364 up
lmo2783	similar to cellobiose phosphotransferase system enzyme IIC	1.2	17.189 up
lmo2799	similar to phosphotransferase system mannitol-specific enzyme IIBC	1.2	18.475 up
lmo2816	similar to transport protein	1.2	9.502 up
lmo2850	similar to sugar transport proteins	1.2	21.620 up
lmo0358	similar to PTS system. fructose-specific enzyme IIBC component	1.2	2.452 up
lmo0507	similar to PTS system. Galactitol-specific IIB component	1.2	2.487 up
lmo0398	similar to phosphotransferase system enzyme IIA	1.2	3.361 up
lmo0503	similar to PTS fructose-specific enzyme IIA component	1.2	4.361 up
lmo0631	similar to PTS system. fructose-specific IIA component	1.2	2.922 up
lmo2137	similar to PTS system. fructose-specific enzyme IIA component	1.2	4.918 up
lmo2651	similar to mannitol-specific PTS enzyme IIA component	1.2	33.793 up
lmo2667	similar to PTS system galactitol-specific enzyme IIA component	1.2	8.907 up
kdpB	potassium-transporting atpase b chain	1.2	2.615 up
kdpA	highly similar to potassium-transporting atpase a chain	1.2	2.860 up
kdpC	potassium-transporting atpase c chain	1.2	2.308 up
lmo2649	similar to hypothetical PTS enzyme IIC component	1.2	48.264 up
lmo0608	similar to ABC transporter. ATP-binding protein	1.2	2.181 up
lmo0986	similar to antibiotic ABC transporter. ATP-binding protein.	1.2	13.445 up
lmo0383	highly similar to B. subtilis methylmalonate-semialdehyde dehydrogenase IolA	2.1.1	8.293 up
lmo0878	similar to oxidoreductases	2.1.1	5.696 up
glpD	similar to glycerol 3 phosphate dehydrogenase	2.1.1	2.850 up
lmo1538	similar to glycerol kinase	2.1.1	3.258 up
lmo2584	similar to formate dehydrogenase associated protein	2.1.1	3.892 up
lmo0386	similar to B. subtilis IolD protein. to acetolactate synthase	2.1.1	5.058 up
lmo2663	similar to polyol dehydrogenase	2.1.1	21.145 up
lmo2664	similar to sorbitol dehydrogenase	2.1.1	16.266 up
lmo0105	highly similar to chitinase B	2.1.1	43.506 up
lmo0300	similar to phospho-beta-glucosidase and phospho-beta-galactosidase	2.1.1	13.810 up
lmo0319	similar to phospho-beta-glucosidase	2.1.1	3.403 up
lmo0345	similar to sugar-phosphate isomerase	2.1.1	5.345 up
lmo0347	similar to dihydroxyacetone kinase	2.1.1	5.598 up
lmo0348	similar to dihydroxyacetone kinase	2.1.1	6.555 up
lmo0384	similar to B. subtilis IolB protein	2.1.1	9.531 up
lmo0385	similar to B. subtilis IolC protein and to fructokinase	2.1.1	5.751 up
lmo0401	highly similar to E. col YbgG protein. a putative sugar hydrolase	2.1.1	20.009 up
lmo0536	similar to 6-phospho-beta-glucosidase	2.1.1	5.857 up
lmo0634	similar to an E. coli putative tagatose 6-phosphate kinase	2.1.1	3.930 up
lmo0643	similar to putative transaldolase	2.1.1	4.796 up
lmo0877	similar to B. subtilis NagB protein (glucosamine-6-phosphate isomerase)	2.1.1	3.630 up
lmo0917	similar to beta-glucosidase	2.1.1	103.336 up
lmo0957	similar to glucosamine-6-Phoasphate isomerase (EC 5.3.1.10)	2.1.1	2.653 up
lmo1729	similar to beta-glucosidases	2.1.1	2.971 up
lmo1883	similar to chitinases	2.1.1	6.114 up
lmo2121	similar to maltosephosphorylase	2.1.1	23.635 up
lmo2661	similar to ribulose-5-phosphate 3-epimerase	2.1.1	16.768 up

lmo2662	similar to ribose 5-phosphate epimerase	2.1.1	13.639 up
lmo2674	similar to ribose 5-phosphate epimerase	2.1.1	4.027 up
lmo2734	weakly similar to sugar hydrolase	2.1.1	6.173 up
lmo2735	similar to Sucrose phosphorylase	2.1.1	7.069 up
lmo2743	similar to transaldolase	2.1.1	4.035 up
lmo2771	similar to beta-glucosidase	2.1.1	3.346 up
lmo2781	similar to beta-glucosidase	2.1.1	30.740 up
lmo2801	similar to a putative N-acetylmannosamine-6-phosphate epimerase	2.1.1	40.473 up
lmo2847	highly similar to rhamnulose-1-phosphate aldolase	2.1.1	5.645 up
lmo2848	highly similar to L-rhamnose isomerase	2.1.1	12.426 up
lmo2849	similar to rhamnulokinase	2.1.1	17.831 up
lmo0505	similar to ribulose-5-phosphate 3-epimerase	2.1.1	2.785 up
lmo0956	similar to N-acetylglucosamine-6P-phosphate deacetylase (EC 3.5.1.25)	2.1.1	2.041 up
lmo1728	some similarities to cellobiose-phosphorylase	2.1.1	2.358 up
lmo2134	similar to fructose-1.6-biphosphate aldolase type II	2.1.1	2.224 up
lmo2659	similar to ribulose-phosphate 3-epimerase	2.1.1	3.035 up
lmo2660	similar to transketolase	2.1.1	4.089 up
lmo2696	similar to hypothetical dihydroxyacetone kinase	2.1.1	2.110 up
lmo2172	similar to propionate CoA-transferase	2.1.1	3.028 up
lmo0502	similar to putative sugar-phosphate isomerase	2.1.1	6.171 up
lmo1998	similar to opine catabolism protein	2.1.1	6.294 up
lmo1999	weakly similar to glucosamine-fructose-6-phosphate aminotransferase	2.1.1	10.879 up
lmo0344	similar to dehydrogenase/reductase	2.1.1	6.249 up
lmo2175	similar to dehydrogenase	2.1.1	11.559 up
lmo2586	similar to formate dehydrogenase alpha chain	2.1.1	10.877 up
lmo2800	similar to dehydrogenase	2.1.1	19.916 up
lmo2122	similar to maltodextrose utilization protein MalA	2.1.1	17.860 up
leuB	similar to 3-isopropylmalate dehydrogenase	2.2	2.954 up
lmo0039	similar to carbamate kinase	2.2	2.076 up
lmo0265	similar to succinyldiaminopimelate desuccinylase	2.2	2.498 up
lmo1348	similar to aminomethyltransferase	2.2	2.629 up
lmo1349	similar to glycine dehydrogenase (decarboxylating) subunit 1	2.2	9.321 up
lmo1350	similar to glycine dehydrogenase (decarboxylating) subunit 2	2.2	4.750 up
argB	highly similar to N-acetylglutamate 5-phosphotransferase	2.2	4.836 up
argJ	highly similar to ornithine acetyltransferase and amino-acid acetyltransferases	2.2	7.591 up
argC	similar to N-acetylglutamate gamma-semialdehyde dehydrogenases	2.2	4.334 up
ilvN	similar to acetolactate synthase (acetohydroxy-acid synthase) (small subunit)	2.2	3.287 up
leuA	similar to 2-isopropylmalate synthase	2.2	3.654 up
leuC	similar to 3-isopropylmalate dehydratase (large subunit)	2.2	2.889 up
leuD	similar to 3-isopropylmalate dehydratase (small subunit)	2.2	2.656 up
ilvA	similar to threonine dehydratase	2.2	2.947 up
argG	similar to argininosuccinate synthase	2.2	4.368 up
argH	similar to argininosuccinate lyase	2.2	5.981 up
argF	highly similar to ornithine carbamoyltransferase	2.2	2.258 up
argD	highly similar to N-acetylornithine aminotransferase	2.2	3.658 up
ilvB	similar to acetolactate synthase (acetohydroxy-acid synthase) (large subunit)	2.2	3.381 up
ilvC	similar to ketol-acid reductoisomerase (acetohydroxy-acid isomeroreductase)	2.2	3.737 up
lmo2647	similar to creatinine amidohydrolase	2.2	144.279 up

lmo2817	similar to peptidases	2.2	4.167 up
lmo2819	similar to carboxypeptidase	2.2	2.698 up
lmo0113	similar to protein gp35 from Bacteriophage A118	4.3	2.892 up
lmo0123	similar to protein gp18 from Bacteriophage A118	4.3	2.046 up
lmo0127	weakly similar to protein gp20 from Bacteriophage A118	4.3	2.074 up
lmo2279	holin [Bacteriophage A118]	4.3	2.002 up
lmo2281	protein gp22 [Bacteriophage A118]	4.3	2.399 up
lmo2282	protein gp21 [Bacteriophage A118]	4.3	2.290 up
lmo2285	Protein gp18 [Bacteriophage A118]	4.3	2.063 up
lmo2288	Protein gp15 [Bacteriophage A118]	4.3	2.511 up
lmo2289	Protein gp14 [Bacteriophage A118]	4.3	2.047 up
lmo2292	Portein gp11 [Bacteriophage A118]	4.3	2.000 up
lmo2298	Protein gp4 [Bacteriophage A118]	4.3	2.563 up
lmo2299	putative portal protein [Bacteriophage A118]	4.3	2.742 up
lmo2303	Protein gp66 [Bacteriophage A118]	4.3	2.974 up
lmo2304	Bacteriophage A118 gp65 protein	4.3	2.331 up
lmo2306	similar to phage protein	4.3	2.759 up
lmo2315	similar to protein gp51 [Bacteriophage A118]	4.3	2.405 up
lmo2322	gp44 [Bacteriophage A118]	4.3	2.217 up
lmo2323	gp43 [Bacteriophage A118]	4.3	2.013 up
lmo2326	similar to protein gp41 [Bacteriophage A118]	4.3	2.602 up
lmo2301	similar to putative terminase small subunit from Bacteriophage A118	4.3	2.780 up
lmo2317	similar to protein gp49 [Bacteriophage A118]	4.3	2.253 up
lmo0128	similar to a protein from Bacteriophage phi-105 (ORF 45)	4.3	2.131 up
lmo2287	putative tape-measure [Bacteriophage A118]	4.3	2.144 up
lmo2300	putative terminase large subunit from Bacteriophage A118	4.3	2.019 up
lmo2286	Protein gp17 [Bacteriophage A118]	4.3	2.261 up
lmo2294	Protein gp9 [Bacteriophage A118]	4.3	2.142 up
lmo0471	hypothetical protein	5.2	2.147 up
lmo2742	hypothetical protein	5.2	5.680 up
lmo0119	hypothetical protein	5.2	2.224 up
lmo0879	hypothetical protein	5.2	3.847 up
lmo2160	similar to unknown proteins	5.2	4.678 up
lmo2161	hypothetical protein	5.2	5.420 up
lmo2162	similar to unknown proteins	5.2	7.140 up
lmo2736	conserved hypothetical protein	5.2	3.193 up
lmo2511	similar to conserved hypothetical proteins like to B. subtilis YvyD protein	5.2	4.051 up
lmo0955	hypothetical protein	5.2	5.585 up
lmo2391	conserved hypothetical protein similar to B. subtilis YhfK protein	5.2	2.256 up
lmo0629	hypothetical protein	5.2	2.377 up
lmo0134	similar to E. coli YjdJ protein	5.2	2.746 up
lmo0323	similar to unknown proteins	5.2	2.845 up
lmo0584	conserved hypothetical membrane protein	5.2	3.325 up
lmo0869	hypothetical protein	5.2	2.975 up
lmo1966	similar to unknown proteins	5.2	4.594 up
lmo0133	similar to E. coli YjdI protein	5.2	2.415 up
lmo0351	similar to unknown proteins	5.2	2.768 up
lmo1140	hypothetical protein	5.2	2.322 up

lmo1241	hypothetical protein	5.2	2.147 up
lmo1718	similar to putative outer surface protein	5.2	4.214 up
lmo2487	similar to B. subtilis YvlB protein	5.2	8.833 up
lmo2570	hypothetical protein	5.2	2.521 up
lmo2585	similar to B. subtilis YrhD protein	5.2	11.917 up
lmo2670	conserved hypothetical protein	5.2	2.899 up
lmo2846	highly similar to B. subtilis YulD protein	5.2	3.303 up
lmo0047	hypothetical protein	5.2	2.229 up
lmo0987	similar to Streptococcus agalactiae CylB protein	5.2	2.644 up
lmo2486	hypothetical protein	5.2	2.402 up
lmo2697	hypothetical protein	5.2	2.230 up
lmo0515	conserved hypothetical protein	5.2	2.850 up
lmo1580	similar to unknown protein	5.2	2.612 up
lmo2673	conserved hypothetical protein	5.2	3.022 up
lmo2645	hypothetical protein	5.2	142.954 up
