# Supplementary material for: Changes in Gene Expression during Adaptation of Listeria monocytogenes to the Soil Environment
Source: PLoS One. 2011 Sep 23;6(9):e24881. doi: 10.1371/journal.pone.0024881 (PMC3179493; doi:10.1371/journal.pone.0024881)
Supplement: Table S6 — Gene ontology «functional categories » at time 18 h. (PDF) [file pone.0024881.s006.pdf]

Table S6. Gene ontology « functional categories » 18 h

name	description	category	fold change
lmo2818	similar to transmembrane efflux protein	1.2	4.878 up
lmo0021	similar to PTS system, fructose-specific IIA component	1.2	12.812 up
lmo0022	similar to PTS system, fructose-specific IIB component	1.2	19.697 up
lmo0023	similar to PTS system, fructose-specific IIC component	1.2	14.374 up
lmo0024	similar to PTS system, mannose-specific IID component	1.2	25.296 up
lmo0027	similar to PTS system, beta-glucosides specific enzyme IIABC	1.2	41.540 up
lmo0298	similar to PTS beta-glucoside-specific enzyme IIC component	1.2	15.822 up
lmo0299	similar to PTS beta-glucoside-specific enzyme IIB component	1.2	17.041 up
lmo0301	similar to PTS beta-glucoside-specific enzyme IIA component	1.2	12.663 up
lmo0400	similar to fructose-specific phosphotransferase enzyme IIC	1.2	7.580 up
lmo0544	similar to PTS system, glucitol/sorbitol-specific enzyme II CII component	1.2	7.390 up
lmo0914	similar to PTS system, IIB component	1.2	21.601 up
lmo0915	similar to phosphotransferase system enzyme IIC	1.2	25.705 up
lmo0916	similar to phosphotransferase system enzyme IIA	1.2	49.999 up
lmo1719	similar to phosphotransferase system (PTS) lichenan-specific enzyme IIA component	1.2	4.219 up
lmo1720	similar to phosphotransferase system (PTS) lichenan-specific enzyme IIB component	1.2	3.472 up
lmo1730	similar to sugar ABC transporter binding protein	1.2	12.135 up
lmo2000	similar to PTS mannose-specific enzyme IID component	1.2	13.563 up
lmo2002	similar to PTS mannose-specific enzyme IIB component	1.2	7.982 up
lmo2123	similar to maltodextrin ABC-transport system (permease)	1.2	17.192 up
lmo2125	similar to maltose/maltodextrin ABC-transporter (binding protein)	1.2	14.136 up
lmo2650	similar to hypothetical PTS enzyme IIB component	1.2	29.323 up
lmo2665	similar to PTS system galactitol-specific enzyme IIC component	1.2	20.652 up
lmo2666	similar to PTS system galactitol-specific enzyme IIB component	1.2	17.375 up
lmo2683	similar to cellobiose phosphotransferase enzyme IIB component	1.2	5.149 up
lmo2684	similar to cellobiose phosphotransferase enzyme IIC component	1.2	7.871 up
lmo2685	similar to cellobiose phosphotransferase enzyme IIA component	1.2	11.108 up
lmo2708	similar to PTS system, cellobiose-specific enzyme IIC	1.2	9.915 up
lmo2782	similar to PTS, cellobiose-specific IIB component	1.2	31.364 up
lmo2783	similar to cellobiose phosphotransferase system enzyme IIC	1.2	17.189 up
lmo2799	similar to phosphotransferase system mannitol-specific enzyme IIBC	1.2	18.475 up
lmo2816	similar to transport protein	1.2	9.502 up
lmo2850	similar to sugar transport proteins	1.2	21.620 up
lmo2651	similar to mannitol-specific PTS enzyme IIA component	1.2	33.793 up
lmo2667	similar to PTS system galactitol-specific enzyme IIA component	1.2	8.907 up
kdpB	potassium-transporting atpase b chain	1.2	2.615 up
lmo2649	similar to hypothetical PTS enzyme IIC component	1.2	48.264 up
glpD	similar to glycerol 3 phosphate dehydrogenase	2.1.1	2.850 up
lmo2663	similar to polyol dehydrogenase	2.1.1	21.145 up
lmo2664	similar to sorbitol dehydrogenase	2.1.1	16.266 up
lmo0105	highly similar to chitinase B	2.1.1	43.506 up
lmo0300	similar to phospho-beta-glucosidase and phospho-beta-galactosidase	2.1.1	13.810 up
lmo0348	similar to dihydroxyacetone kinase	2.1.1	6.555 up
lmo0401	highly similar to E. col YbgG protein, a putative sugar hydrolase	2.1.1	20.009 up
lmo0643	similar to putative transaldolase	2.1.1	4.796 up
lmo0877	similar to B. subtilis NagB protein (glucosamine-6-phosphate isomerase)	2.1.1	3.630 up

lmo0917	similar to beta-glucosidase	2.1.1	103.336 up
lmo0957	similar to glucosamine-6-Phoasphate isomerase (EC 5.3.1.10)	2.1.1	2.653 up
lmo1883	similar to chitinases	2.1.1	6.114 up
lmo2121	similar to maltosephosphorylase	2.1.1	23.635 up
lmo2661	similar to ribulose-5-phosphate 3-epimerase	2.1.1	16.768 up
lmo2662	similar to ribose 5-phosphate epimerase	2.1.1	13.639 up
lmo2674	similar to ribose 5-phosphate epimerase	2.1.1	4.027 up
lmo2734	weakly similar to sugar hydrolase	2.1.1	6.173 up
lmo2735	similar to Sucrose phosphorylase	2.1.1	7.069 up
lmo2781	similar to beta-glucosidase	2.1.1	30.740 up
lmo2801	similar to a putative N-acetylmannosamine-6-phosphate epimerase	2.1.1	40.473 up
lmo2849	similar to rhamnulokinase	2.1.1	17.831 up
lmo2172	similar to propionate CoA-transferase	2.1.1	3.028 up
lmo0344	similar to dehydrogenase/reductase	2.1.1	6.249 up
lmo2175	similar to dehydrogenase	2.1.1	11.559 up
lmo2586	similar to formate dehydrogenase alpha chain	2.1.1	10.877 up
lmo2122	similar to maltodextrose utilization protein MalA	2.1.1	17.860 up
lmo2288	Protein gp15 [Bacteriophage A118]	4.3	2.511 up
lmo2298	Protein gp4 [Bacteriophage A118]	4.3	2.563 up
lmo2303	Protein gp66 [Bacteriophage A118]	4.3	2.974 up
lmo2306	similar to phage protein	4.3	2.759 up
lmo2315	similar to protein gp51 [Bacteriophage A118]	4.3	2.405 up
lmo2322	gp44 [Bacteriophage A118]	4.3	2.217 up
lmo2326	similar to protein gp41 [Bacteriophage A118]	4.3	2.602 up
lmo2301	similar to putative terminase small subunit from Bacteriophage A118	4.3	2.780 up
lmo2742	hypothetical protein	5.2	5.680 up
lmo0879	hypothetical protein	5.2	3.847 up
lmo0584	conserved hypothetical membrane protein	5.2	3.325 up
lmo1718	similar to putative outer surface protein	5.2	4.214 up
lmo2585	similar to B. subtilis YrhD protein	5.2	11.917 up
lmo2846	highly similar to B. subtilis YulD protein	5.2	3.303 up
lmo0515	conserved hypothetical protein	5.2	2.850 up
lmo2673	conserved hypothetical protein	5.2	3.022 up
lmo2645	hypothetical protein	5.2	142.954 up
