# Supplementary material for: Changes in Gene Expression during Adaptation of Listeria monocytogenes to the Soil Environment
Source: PLoS One. 2011 Sep 23;6(9):e24881. doi: 10.1371/journal.pone.0024881 (PMC3179493; doi:10.1371/journal.pone.0024881)
Supplement: Table S7 — List of genes with higher and lower transcript levels at time 15 min, 30 min and 18 h identified after gene ontology with ontology terms “CodY”, “PrfA”, “SigmaB” and “PrfA/SigmaB”. (PDF) [file pone.0024881.s007.pdf]

Table S7. List of genes with higher and lower transcript levels at time 15 min identified after gene ontology with ontology terms “CodY”,” PrfA”,” SigmaB” and”PrfA/SigmaB”.

name	description	Functional category	Regulon	Fold change
argB	highly similar to N-acetylglutamate 5-phosphotransferase	2.2	CodY	2,314 up
argG	similar to argininosuccinate synthase	2.2	CodY	4,707 up
argH	similar to argininosuccinate lyase	2.2	CodY	5,961 up
argJ	highly similar to ornithine acetyltransferase and amino-acid acetyltransferases	2.2	CodY	3,770 up
lmo0022	similar to PTS system, fructose-specific IIB component	1.2	CodY	19,035 up
lmo0298	similar to PTS beta-glucoside-specific enzyme IIC component	1.2	CodY	13,358 up
lmo0299	similar to PTS beta-glucoside-specific enzyme IIB component	1.2	CodY	13,398 up
lmo0344	similar to dehydrogenase/reductase	2.1.1	CodY	2,317 up
lmo0345	similar to sugar-phosphate isomerase	2.1.1	CodY	2,632 up
lmo1997	similar to PTS mannose-specific enzyme IIA component	1.2	CodY	5,834 up
lmo1998	similar to opine catabolism protein	2.1.1	CodY	4,561 up
lmo1999	weakly similar to glucosamine-fructose-6-phosphate aminotransferase	2.1.1	CodY	7,159 up
lmo2001	similar to PTS mannose-specific enzyme IIC component	1.2	CodY	6,605 up
lmo2137	similar to PTS system, fructose-specific enzyme IIA component	1.2	CodY	5,052 up
lmo2585	similar to B. subtilis YrhD protein	5.2	CodY	7,101 up
lmo2646		6.0	CodY	32,680 up
lmo2647	similar to creatinine amidohydrolase	2.2	CodY	53,486 up
lmo2661	similar to ribulose-5-phosphate 3-epimerase	2.1.1	CodY	8,132 up
lmo2662	similar to ribose 5-phosphate epimerase	2.1.1	CodY	8,669 up
lmo2663	similar to polyol dehydrogenase	2.1.1	CodY	9,744 up
lmo2664	similar to sorbitol dehydrogenase	2.1.1	CodY	9,634 up
lmo2666	similar to PTS system galactitol-specific enzyme IIB component	1.2	CodY	15,747 up
lmo0269	similar to transporter	1.2	CodY	6.234 down
lmo2469	similar to amino acid transporter	1.2	CodY	2.302 down
motB	similar to motility protein (flagellar motor rotation) MotB	1.5	CodY	2.007 down
lmo0769	similar to alpha-1,6-mannanase	2.1.1	CodY	2.114 down
galE	UDP-glucose 4-epimerase	2.1.1	PrfA	2.292 down
lmo2829	similar to yeast protein Frm2p involved in fatty acid signaling	2.4	CodY	3.171 down
rpsP	ribosomal protein S16	3.7.1	CodY	2.448 down

lmo0242	similar to B. subtilis Yacp protein	5.2	PrfA	2.245 down
lmo0788	hypothetical protein	5.2	PrfA	2.743 down
lmo2127	hypothetical protein	5.2	CodY	3.305 down

List of genes with higher and lower transcript levels at time 30 min identified after gene ontology with ontology terms “CodY”,” PrfA”,” SigmaB” and”PrfA/SigmaB”.

name	description	Functional category	Regulon	Fold change
argB	highly similar to N-acetylglutamate 5-phosphotransferase	2.2	CodY	4,836 up
argG	similar to argininosuccinate synthase	2.2	CodY	4,368 up
argH	similar to argininosuccinate lyase	2.2	CodY	5,981 up
argJ	highly similar to ornithine acetyltransferase and amino-acid acetyltransferases	2.2	CodY	7,591 up
lmo0022	similar to PTS system, fructose-specific IIB component	1.2	CodY	19,697 up
lmo0298	similar to PTS beta-glucoside-specific enzyme IIC component	1.2	CodY	15,822 up
lmo0299	similar to PTS beta-glucoside-specific enzyme IIB component	1.2	CodY	17,041 up
lmo0344	similar to dehydrogenase/reductase	2.1.1	CodY	6,249 up
lmo0345	similar to sugar-phosphate isomerase	2.1.1	CodY	5,345 up
lmo1997	similar to PTS mannose-specific enzyme IIA component	1.2	CodY	6,082 up
lmo1998	similar to opine catabolism protein	2.1.1	CodY	6,294 up
lmo1999	weakly similar to glucosamine-fructose-6-phosphate aminotransferase	2.1.1	CodY	10,879 up
lmo2001	similar to PTS mannose-specific enzyme IIC component	1.2	CodY	7,748 up
lmo2137	similar to PTS system, fructose-specific enzyme IIA component	1.2	CodY	4,918 up
lmo2585	similar to B. subtilis YrhD protein	5.2	CodY	11,917 up
lmo2646		6.0	CodY	84,283 up
lmo2647	similar to creatinine amidohydrolase	2.2	CodY	144,279 up
lmo2661	similar to ribulose-5-phosphate 3-epimerase	2.1.1	CodY	16,768 up
lmo2662	similar to ribose 5-phosphate epimerase	2.1.1	CodY	13,639 up

lmo2663	similar to polyol dehydrogenase	2.1.1	CodY	21,145 up
lmo2664	similar to sorbitol dehydrogenase	2.1.1	CodY	16,266 up
lmo2666	similar to PTS system galactitol-specific enzyme IIB component	1.2	CodY	17,375 up
plcA	phosphatidylinositol-specific phospholipase c	2.4	PrfA	3.726 down
mpl	Zinc metalloproteinase precursor	2.2	PrfA	2.851 down
lmo0242	similar to B. subtilis Yacp protein	5.2	PrfA	2.980 down
lmo0269	similar to transporter	1.2	CodY	15.999 down
lmo0560	similar to NADP-specific glutamate dehydrogenase	2.2	CodY	3.157 down
lmo0654	hypothetical protein	6.0	PrfA	4.301 down
motB	similar to motility protein (flagellar motor rotation) MotB	1.5	CodY	2.073 down
lmo0701	hypothetical protein	1.8	CodY	2.132 down
lmo0729	hypothetical protein	6.0	CodY	2.180 down
lmo0768	similar to sugar ABC transporter, periplasmic sugar-binding protein	1.2	CodY	2.649 down
lmo0769	similar to alpha-1,6-mannanase	2.1.1	CodY	2.989 down
lmo0788	hypothetical protein	5.2	PrfA	3.816 down
lmo0937	hypothetical protein	6.0	PrfA	4.352 down
opuCB	similar to glycine betaine/carnitine/choline ABC transporter (membrane protein)	1.2	PrfA,Sigma B	2.147 down
opuCA	similar to glycine betaine/carnitine/choline ABC transporter (ATP-binding protein)	1.2	PrfA,Sigma B	9.492 down
lmo1516	similar to ammonium transporter NrgA	1.2	CodY	2.980 down
lmo1517	similar to nitrogen regulatory PII protein	2.2	CodY	3.125 down
lmo1734	similar to glutamate synthase (large subunit)	2.2	CodY	2.095 down
rpsP	ribosomal protein S16	3.7.1	CodY	3.752 down
lmo2127	hypothetical protein	5.2	CodY	8.309 down
lmo2219	similar to post-translocation molecular chaperone	1.6	PrfA	2.097 down
lmo2469	similar to amino acid transporter	1.2	CodY	5.495 down
galE	UDP-glucose 4-epimerase	2.1.1	PrfA	4.028 down
lmo2721	similar to glucosamine-6-phosphate isomerase	2.1.1	CodY	2.589 down
lmo2775	hypothetical membrane protein	1.2	CodY	2.706 down
lmo2803	hypothetical protein	6.0	CodY	3.195 down

lmo2829	similar to yeast protein Frm2p involved in fatty acid signaling	2.4	CodY	9.549 down

List of genes with higher and lower transcript levels at time 18 h identified after gene ontology with ontology terms “CodY”,” PrfA”,” SigmaB” and”PrfA/SigmaB”

name	description	Functional category	Regulon	Fold change
argG	similar to argininosuccinate synthase	2.2	CodY	2,002 up
lmo0022	similar to PTS system, fructose-specific IIB component	1.2	CodY	4,137 up
lmo0298	similar to PTS beta-glucoside-specific enzyme IIC component	1.2	CodY	5,698 up
lmo0299	similar to PTS beta-glucoside-specific enzyme IIB component	1.2	CodY	8,432 up
lmo0344	similar to dehydrogenase/reductase	2.1.1	CodY	3,672 up
lmo2585	similar to B. subtilis YrhD protein	5.2	CodY	5,771 up
lmo2646		6.0	CodY	48,757 up
lmo2647	similar to creatinine amidohydrolase	2.2	CodY	50,049 up
lmo2661	similar to ribulose-5-phosphate 3-epimerase	2.1.1	CodY	8,397 up
lmo2662	similar to ribose 5-phosphate epimerase	2.1.1	CodY	11,375 up
lmo2663	similar to polyol dehydrogenase	2.1.1	CodY	16,199 up
lmo2664	similar to sorbitol dehydrogenase	2.1.1	CodY	9,926 up
lmo2666	similar to PTS system galactitol-specific enzyme IIB component	1.2	CodY	7,420 up
lmo2659	similar to ribulose-phosphate 3-epimerase	2.1.1	CodY	2,349 up
lmo2660	similar to transketolase	2.1.1	CodY	3,527 up
lmo0269	similar to transporter	1.2	CodY	19.905 down
lmo0768	similar to sugar ABC transporter, periplasmic sugar-binding protein	1.2	CodY	5.303 down
lmo1516	similar to ammonium transporter NrgA	1.2	CodY	10.918 down
lmo1652	similar to ABC transporter (ATP-binding protein)	1.2	CodY	2.067 down
lmo2097	similar to PTS system galactitol-specific enzyme IIB component	1.2	CodY	4.027 down
lmo2469	similar to amino acid transporter	1.2	CodY	6.557 down
lmo2775	hypothetical membrane protein	1.2	CodY	2.098 down
motB	similar to motility protein (flagellar motor rotation) MotB	1.5	CodY	3.299 down

lmo0707	similar to flagellar hook-associated protein 2 FliD	1.5	CodY	4.217 down
lmo0708	similar to hypothetical flagellar protein	1.5	CodY	2.271 down
lmo0710	similar to flagellar basal-body rod protein flgB	1.5	CodY	2.375 down
lmo0712	similar to flagellar hook-basal body complex protein FliE	1.5	CodY	2.561 down
lmo0713	similar to flagellar basal-body M-ring protein fliF	1.5	CodY	2.548 down
lmo0714	similar to flagellar motor switch protein fliG	1.5	CodY	2.016 down
lmo0701	hypothetical protein	1.8	CodY	6.617 down
lmo1799	putative peptidoglycan bound protein (LPXTG motif)	1.8	CodY	3.006 down
lmo0769	similar to alpha-1,6-mannanase	2.1.1	CodY	7.872 down
lmo0813	similar to fructokinases	2.1.1	CodY	2.017 down
lmo2721	similar to glucosamine-6-phosphate isomerase	2.1.1	CodY	2.622 down
lmo0359	similar to D-fructose-1,6-biphosphate aldolase	2.1.2	CodY	3.683 down
lmo0458	similar to hydantoinase	2.2	CodY	7.420 down
lmo0560	similar to NADP-specific glutamate dehydrogenase	2.2	CodY	11.762 down
hisF	highly similar to cyclase HisF	2.2	CodY	4.101 down
hisA	highly similar to phosphoribosylformimino-5-aminoimidazole carboxamide ribotide isomerase	2.2	CodY	4.625 down
hisH	similar to amidotransferases	2.2	CodY	2.737 down
hisB	imidazoleglycerol-phosphate dehydratase	2.2	CodY	5.321 down
hisD	highly similar to histidinol dehydrogenases	2.2	CodY	4.868 down
hisG	similar to ATP phosphoribosyltransferase	2.2	CodY	4.459 down
lmo1517	similar to nitrogen regulatory PII protein	2.2	CodY	10.387 down
trpD	highly similar to anthranilate phosphoribosyltransferase	2.2	CodY	2.675 down
lmo1733	similar to glutamate synthase (small subunit)	2.2	CodY	2.995 down
lmo1734	similar to glutamate synthase (large subunit)	2.2	CodY	4.560 down
lmo2829	similar to yeast protein Frm2p involved in fatty acid signaling	2.4	CodY	9.362 down
lmo1196	similar to precorrin decarbocylase	2.5	CodY	7.249 down
rpsP	ribosomal protein S16	3.7.1	CodY	5.820 down
lmo0422	similar to unknown protein	5.2	CodY	3.241 down
lmo1649	hypothetical protein	5.2	CodY	4.108 down
lmo2127	hypothetical protein	5.2	CodY	11.794 down

lmo0687	hypothetical protein	6.0	CodY	5.893 down
lmo0702	hypothetical protein	6.0	CodY	3.474 down
lmo0709	hypothetical protein	6.0	CodY	2.549 down
lmo0715	flagellar assembly protein H	6.0	CodY	2.222 down
lmo0729	hypothetical protein	6.0	CodY	14.208 down
lmo2803	hypothetical protein	6.0	CodY	13.163 down
lmo2804	hypothetical protein	6.0	CodY	5.132 down
lmo0169	similar to a glucose uptake protein	1.2	PrfA,Sigma B	6.332 down
lmo0781	similar to mannose-specific phosphotransferase system (PTS) component IID	1.2	PrfA,Sigma B	4.114 down
opuCD	similar to betaine/carnitine/choline ABC transporter (membrane p)	1.2	PrfA,Sigma B	3.017 down
opuCC	similar to glycine betaine/carnitine/choline ABC transporter (osmoprotectant-binding protein)	1.2	PrfA,Sigma B	2.095 down
opuCB	similar to glycine betaine/carnitine/choline ABC transporter (membrane protein)	1.2	PrfA,Sigma B	4.858 down
opuCA	similar to glycine betaine/carnitine/choline ABC transporter (ATP-binding protein)	1.2	PrfA,Sigma B	15.392 down
lmo0676	similar to flagellar biosynthesic protein FliP	1.5	PrfA,Sigma B	2.740 down
lmo2067	similar to conjugated bile acid hydrolase	4.2	PrfA,Sigma B	2.048 down
lmo0439	weakly similar to a module of peptide synthetase	4.5	PrfA,Sigma B	3.765 down
lmo0796	conserved hypothetical protein	5.2	PrfA,Sigma B	3.355 down
lmo2231	similar to unknown proteins	5.2	PrfA,Sigma B	2.740 down
lmo2570	hypothetical protein	5.2	PrfA,Sigma B	2.515 down
lmo1261	hypothetical protein	6.0	PrfA,Sigma B	2.920 down

iap	P60 extracellular protein, invasion associated protein Iap	1.1	SigmaB	20.543 down
mreD	similar to cell-shape determining protein MreD	1.1	SigmaB	5.227 down
pbpB	similar to penicillin-binding protein 2B	1.1	SigmaB	2.619 down
spl	peptidoglycan lytic protein P45	1.1	SigmaB	10.045 down
lmo2554	similar to galactosyltransferase	1.1	SigmaB	4.995 down
lmo0405	similar to phosphate transport protein	1.2	SigmaB	2.713 down
lmo0593	similar to transport proteins (formate?)	1.2	SigmaB	3.349 down
lmo0738	similar to phosphotransferase system (PTS) beta-glucoside-specific enzyme IIABC component	1.2	SigmaB	3.892 down
lmo1421	similar to glycine betaine/carnitine/choline ABC transporter (ATP-binding protein)	1.2	SigmaB	3.600 down
lmo1422	similar to glycine betaine/carnitine/choline ABC transporter (membrane protein)	1.2	SigmaB	2.028 down
lmo1424	similar to manganese transport proteins NRAMP	1.2	SigmaB	2.292 down
lmo1431	similar to ABC transporter (ATP-binding protein)	1.2	SigmaB	12.551 down
lmo1724	similar to ABC transporter, ATP-binding protein	1.2	SigmaB	23.030 down
lmo1746	similar to ABC transporter (permease)	1.2	SigmaB	6.635 down
lmo1847	similar to adhesion binding proteins and lipoproteins with multiple specificity for metal cations (ABC transporter)	1.2	SigmaB	4.733 down
lmo1848	similar metal cations ABC transporter (permease protein)	1.2	SigmaB	2.933 down
lmo1849	similar to metal cations ABC transporter, ATP-binding proteins	1.2	SigmaB	2.237 down
lmo1875	similar to putative ABC transporters (ATP-binding protein)	1.2	SigmaB	5.928 down
lmo2062	similar to copper export proteins	1.2	SigmaB	29.505 down
lmo2192	similar to oligopeptide ABC transporter (ATP-binding protein)	1.2	SigmaB	3.443 down
lmo2194	similar to oligopeptide ABC transporter (permease)	1.2	SigmaB	3.381 down
lmo2371	similar to putative ABC-transporter transmembrane subunit	1.2	SigmaB	7.968 down
lmo2429	similar to B. subtilis ferrichrome ABC transporter (ATP-binding protein) FhuC	1.2	SigmaB	3.391 down
lmo2463	similar to transport protein	1.2	SigmaB	3.712 down
lmo2777	similar to efflux protein	1.2	SigmaB	8.584 down
lmo2451	similar to preprotein translocase subunit SecG	1.6	SigmaB	4.586 down
secA	translocase binding subunit (ATPase)	1.6	SigmaB	3.748 down

lmo1071	similar to cell-division protein RodA and FtsW	1.7	SigmaB	10.246 down
minD	highly similar to cell division inhibitor (septum placement) protein MinD	1.7	SigmaB	4.413 down
ftsZ	highly similar to cell-division initiation protein FtsZ	1.7	SigmaB	3.015 down
divIB	similar to cell-division initiation protein divIB	1.7	SigmaB	6.492 down
ftsL	similar to cell-division protein FtsL	1.7	SigmaB	66.299 down
ftsX	highly similar to cell-division protein FtsX	1.7	SigmaB	5.700 down
ftsE	highly similar to the cell-division ATP-binding protein FtsE	1.7	SigmaB	4.567 down
lmo1086	similar to CDP-ribitol pyrophosphorylase	2.1	SigmaB	5.814 down
lmo0529	conserved hypothetical protein similar to putative glucosaminyltransferase	2.1.1	SigmaB	3.532 down
lmo0554	similar to NADH-dependent butanol dehydrogenase	2.1.1	SigmaB	2.488 down
lmo0669	similar to oxidoreductase	2.1.1	SigmaB	2.779 down
lmo0739	similar to 6-phospho-beta-glucosidase	2.1.1	SigmaB	21.550 down
lmo0811	similar to carbonic anhydrase	2.1.1	SigmaB	2.916 down
ackA	highly similar to acetate kinase	2.1.1	SigmaB	4.200 down
lmo1818	similar to ribulose-5-phosphate 3-epimerase	2.1.1	SigmaB	10.596 down
lmo2475	similar to phosphomannomutase and phosphoglucomutase	2.1.1	SigmaB	7.829 down
lmo2712	highly similar to gluconate kinase	2.1.1	SigmaB	2.042 down
lmo1375	similar to aminotripeptidase	2.2	SigmaB	9.213 down
lmo1585	similar to proteases	2.2	SigmaB	16.129 down
lmo1681	similar to cobalamin-independent methionine synthase	2.2	SigmaB	5.082 down
hom	highly similar to homoserine dehydrogenase	2.2	SigmaB	21.543 down
lmo0218	polyribonucleotide nucleotidyltransferase domain present	2.3	SigmaB	6.685 down
rncS	similar to ribonuclease III	2.3	SigmaB	11.539 down
lmo0970	similar to enoyl- acyl-carrier protein reductase	2.4	SigmaB	6.227 down
lmo1647	similar to 1-acylglycerol-3-phosphate O-acyltransferases	2.4	SigmaB	12.443 down
fabD	similar to malonyl CoA-acyl carrier protein transacylase	2.4	SigmaB	6.883 down
lmo2201	similar to 3-oxoacyl-acyl-carrier protein synthase	2.4	SigmaB	2.994 down
lmo2450	similar to carboxylesterase	2.4	SigmaB	3.847 down
lmo2503	similar to cardiolipin synthase	2.4	SigmaB	4.800 down
tktB	similar to D-1-deoxyxylulose 5-phosphate synthase	2.5	SigmaB	5.336 down

menF	similar to menaquinone-specific isochorismate synthase	2.5	SigmaB	4.604 down
lmo1677	similar to menaquinone biosynthesis proteins	2.5	SigmaB	16.675 down
lmo2542	similar to protoporphyrinogen oxidase	2.5	SigmaB	2.997 down
lmo2641	similar to heptaprenyl diphosphate synthase component II	2.5	SigmaB	3.088 down
dnaE	highly similar to DNA polymerase III (alpha subunit) DnaE	3.1	SigmaB	3.782 down
parC	highly similar to DNA gyrase-like protein (subunit A)	3.4	SigmaB	2.360 down
lmo1606	similar to DNA translocase	3.4	SigmaB	6.830 down
smc	similar to Smc protein essential for chromosome condensation and partition	3.4	SigmaB	2.918 down
lmo2334	similar to transcriptional regulator	3.5.2	SigmaB	47.450 down
lmo2494	similar to negative regulator of phosphate regulon	3.5.2	SigmaB	2.912 down
lmo1450	similar to ATP-dependent RNA helicase, DEAD-box family (deaD)	3.5.3	SigmaB	4.676 down
lmo1496	similar to transcription elongation factor GreA	3.5.3	SigmaB	11.797 down
tsf	translation elongation factor	3.5.3	SigmaB	4.335 down
lmo2560	similar to B. subtilis RNA polymerase delta subunit	3.5.3	SigmaB	3.257 down
lmo1359	similar to transcription termination protein (NusB)	3.5.4	SigmaB	6.154 down
lmo2244	similar to putative ribosomal large subunit pseudouridine synthase	3.6	SigmaB	6.672 down
rpsT	ribosomal protein S20	3.7.1	SigmaB	7.470 down
rpsD	ribosomal protein S4	3.7.1	SigmaB	5.605 down
rpsI	ribosomal protein S9	3.7.1	SigmaB	5.217 down
lysS	lysyl-tRNA synthetase	3.7.2	SigmaB	2.244 down
alaS	alanyl-tRNA synthetase	3.7.2	SigmaB	9.227 down
hisS	histidyl-tRNA synthetase	3.7.2	SigmaB	11.362 down
infC	translation initiation factor IF-3	3.7.3	SigmaB	2.363 down
frr	highly similar to ribosome recycling factors	3.7.5	SigmaB	6.758 down
lmo1433	similar to glutathione reductase	4.1	SigmaB	2.659 down
lmo1601	similar to general stress protein	4.1	SigmaB	2.250 down
kat	catalase	4.2	SigmaB	3.124 down
lmo0190	similar to B. subtilis YabH protein	5.2	SigmaB	5.057 down
lmo0590	similar to a fusion of two types of conserved hypothetical protein conserved hypothetical	5.2	SigmaB	4.468 down

lmo0591	similar to unknown membrane proteins	5.2	SigmaB	2.737 down
lmo0663	conserved hypothetical proteins	5.2	SigmaB	2.734 down
lmo0670	hypothetical protein	5.2	SigmaB	2.305 down
lmo0911	hypothetical protein	5.2	SigmaB	2.785 down
lmo1351	hypothetical protein	5.2	SigmaB	3.082 down
lmo1358	similar to B. subtilis YqhY protein	5.2	SigmaB	3.698 down
lmo1440	similar to unknown proteins	5.2	SigmaB	36.429 down
lmo1456	similar to unknown proteins	5.2	SigmaB	2.685 down
lmo1488	similar to unknown proteins	5.2	SigmaB	7.557 down
lmo1526	similar to unknown proteins	5.2	SigmaB	2.440 down
lmo1776	similar to unknown protein	5.2	SigmaB	10.284 down
lmo1814	similar to unknown proteins	5.2	SigmaB	5.081 down
lmo1937	similar to unknown protein	5.2	SigmaB	5.792 down
lmo2048	similar to unknown proteins	5.2	SigmaB	11.670 down
lmo2245	similar to unknown proteins	5.2	SigmaB	5.069 down
lmo2254	similar to unknown proteins	5.2	SigmaB	14.388 down
lmo2386	similar to B. subtilis YuiD protein	5.2	SigmaB	4.031 down
lmo2387	conserved hypothetical protein	5.2	SigmaB	3.014 down
lmo2508	similar to conserved hypothetical proteins	5.2	SigmaB	2.489 down
lmo2553	conserved hypothetical protein	5.2	SigmaB	4.530 down
lmo2562	hypothetical protein	5.2	SigmaB	11.772 down
lmo2603	hypothetical protein	5.2	SigmaB	2.805 down
lmo2634	similar to B. subtilis YbaF protein	5.2	SigmaB	3.318 down
lmo0647	hypothetical protein	6.0	SigmaB	3.461 down
lmo0819	hypothetical protein	6.0	SigmaB	2.666 down
lmo1752	hypothetical protein	6.0	SigmaB	7.923 down
lmo2710	hypothetical protein	6.0	SigmaB	11.428 down
