# Supplementary material for: Changes in Gene Expression during Adaptation of Listeria monocytogenes to the Soil Environment
Source: PLoS One. 2011 Sep 23;6(9):e24881. doi: 10.1371/journal.pone.0024881 (PMC3179493; doi:10.1371/journal.pone.0024881)
Supplement: Table S8 — Transcript levels of the major virulence-associated genes and internalin family at time 15 min, 30 min and 18 h. (PDF) [file pone.0024881.s008.pdf]

Table S8. Transcript levels of the major virulence-associated genes and internalin family.

name	description	Functional category	Fold change TA 15 min	Fold change TA 30 min	Fold change TA 18 h	Regulon
prfA	listeriolysin positive regulatory protein	3.5.2	1.75 up	1.38 down	2.25 down	PrfA, σB
plcA	phosphatidylinositol-specific phospholipase c	2.4	1.80 down	3.72 down	3.35 down	PrfA
hly	listeriolysin O precursor	4.5	1.21 down	1.66 down	3.51 down	PrfA, σB
mpl	Zinc metalloproteinase precursor	2.2	1.92 down	2.85 down	-----	PrfA
actA	actin-assembly inducing protein precursor	1.8	1.52 down	1.83 down	5.81 down	PrfA
plcB	phospholipase C	2.4	1.34 down	1.95 down	1.90 down	PrfA, σB
inlA	Internalin A	1.8	1.54 up	1.65 up	1.41 down	PrfA, σB
inlB	Internalin B	1.8	2.61 up	1.49 up	1.51 down	PrfA, σB
inlC	internalin C	1.9	1.33 up	1.20 down	3.60 down	PrfA
inlE	internalin E	1.8	1.59 up	1.14 down	1.42 down	
inlG	internalin G	1.8	2.38 down	3.87 down	4.71 down	
inlH	internalin H	1.8	1.12 up	1.10 down	1.24 up	PrfA, σB
iap	P60 extracellular protein. invasion associated protein	1.1	4.70 down	6.77 down	20.54 down	σB
kat	catalase	4.2	1.22 down	2.11 down	3.12 down	σB
sod	superoxide dismutase	4.2	1.05 down	1.23 down	2.79 down	
lmo0171	similar to internalin proteins. putative peptidoglycan bound protein (LPXTG motif)	1.8	1.26 up	1.31 down	3.77 down	
lmo0327	Similar to cell surface proteins (LPXTG motif)	1.8	2.15 down	2.84 down	2.96 down	
lmo0331	similar to internalin. putative peptidoglycan bound protein (LPXTG motif)	1.8	1.58 down	2.97 down	5.81 down	σB
lmo0333	similar to internalin proteins. putative peptidoglycan bound protein (LPXTG motif)	1.8	2.30 down	7.40 down	6.88 down	
lmo0409	similar to internalin. peptidoglycan bound protein (LPxTG motif)	1.8	1.44 down	2.67 down	5.09 down	
lmo0514	similar to internalin protein. putative peptidoglycan bound protein (LPXTG motif)	1.8	12.52 down	27.49 down	5.89 down	
lmo0549	similar to internalin protein	1.9	2.37 down	6.21 down	4.16 down	
lmo0610	similar to internalin proteins. putative peptidoglycan bound protein (LPXTG motif)	1.8	----	1.23 down	1.07 down	σB
lmo0801	similar to internalin. putative peptidoglycan bound protein (LPXTG motif)	1.8	2.11 down	4.34 down	4.79 down	
lmo1136	similar to internalin. putative peptidoglycan bound protein (LPXTG motif)	1.8	4.21 down	8.92 down	5.64 down	
lmo1289	similar to internalin proteins. putative peptidoglycan bound protein (LPXTG motif)	1.8	1.55 up	1.04 up	1.19 down	
lmo1290	similar to internalin proteins. putative peptidoglycan bound protein (LPXTG motif)	1.8	1.05 down	1.37 down	3.61 down	
lmo2027	putative cell surface protein. similar to internalin proteins	1.9	1.08 down	1.94 down	1.48 down	
lmo2203	similar to N-acetylmuramoyl-L-alanine amidase and to internalin B	1.8	2.94 down	5.01 down	4.16 down	

lmo2396	similar to internalin proteins. putative peptidoglycan bound protein (LPXTG motif)	1.8	2.07 down	2.83 down	3.61 down	
lmo2445	similar to internalin	1.8	1.12 down	1.35 down	1.39 down	
lmo2470	similar to internalin proteins	1.9	1.30 up	1.15 up	1.50 up	
lmo2821	similar to internalin. Unknown. putative peptidoglycan bound protein (LPXTG motif)	1.8	1.03 down	1.25 down	53.30 down	
lmo2115	similar to ABC transporter (permease)	1.2	3.89 down	6.67 down	7.27 down	VirR, σB, σL
lmo2114	similar to ABC transporter (ATP-binding protein)	1.2	4.99 down	5.57 down	5.23 down	VirR, σB, σH, σL, CtsR
dltA	D-alanine-activating enzyme (dae), D-alanine-D-alanyl carrier protein ligase (dcl)	1.1	2.33 down	3.46 down	7.1 down	VirR
dltB	DltB protein for D-alanine esterification of lipoteichoic acid and wall teichoic acid	1.1	1.88 down	3.79 down	3.47 down	VirR
dltC	D-alanyl carrier protein	1.1	1.87 down	2.98 down	13.91 down	VirR
dltD	DltD protein for D-alanine esterification of lipoteichoic acid and wall teichoic acid	1.1	1.72 down	2.74 down	5.48 down	VirR
lmo1695	similar to putative membrane proteins	5.2	2.27 down	3.21 down	5.76 down	VirR
lmo1696	similar to unknown proteins	5.2	7.54 down	22.17 down	21.41 down	VirR
lmo2177	similar to unknown protein	5.2	2.99 down	4.71 down	5.23 down	VirR, σL
lmo0604	similar to B. subtilis YvlA protein	5.2	5.63 down	7.89 down	6.60 down	VirR
lmo2439	hypothetical protein	5.2	2.187 down	4.66 down	8.01 down	VirR
lmo2156	hypothetical protein	5.1	22.39 down	19.91 down	20.62 down	VirR
virR	similar to two-component response regulator	3.5.2	1.61 down	2.93 down	4.97 down	VirR
lmo1744	similar to unknown proteins	5.2	2.39 down	6.07 down	14.68 down	VirR
lmo1743	hypothetical protein	6.0	2.23 down	4.08 down	5.79 down	VirR
adeC	highly similar to adenine deaminases	2.3	2.35 down	3.46 down	5.16 down	VirR
virS	similar to two-component sensor histidine kinase	1.3	1.08 down	1.399 down	3.201 down	VirR
