# Supplementary material for: Changes in Gene Expression during Adaptation of Listeria monocytogenes to the Soil Environment
Source: PLoS One. 2011 Sep 23;6(9):e24881. doi: 10.1371/journal.pone.0024881 (PMC3179493; doi:10.1371/journal.pone.0024881)
Supplement: Table S9 — Differences of gene expression during incubation in compost-amended (DA) and control (TA) soil extract. (PDF) [file pone.0024881.s009.pdf]

Table S9. Differences of gene expression during incubation in compost-amended (DA) and control (TA) soil extract.

<u>15 min Incubation</u>

name	description	Functional category	Fold change TA vs T0	Fold change DA vs T0	Fold change DA vs TA
lmo0834	hypothetical protein	5.1	5.139 down	11.215 down	2.182 down
lmo0835	putative peptidoglycan bound protein (LPXTG motif)	1.8	4.256 down	8.832 down	2.075 down
lmo1333	similar to *B. subtilis* YqzC protein	5.2	1.964 down	4.319 down	2.198 down
lmo1623	similar to unknown proteins	5.2	2.053 down	5.023 down	2.452 down
zurR	transcriptional regulator ZurR (ferric uptake regulation)	3.5.2	2.219 down	5.442 down	2.445 down

<u>30 min Incubation</u>

name	description	Functional category	Fold change TA vs T0	Fold change DA vs T0	Fold change DA vs TA
cspB	similar to major cold-shock protein	4.1	2.181 down	1.023 down	2.131 up
kdpA	highly similar to potassium-transporting atpase a chain	1.2	2.860 up	1.190 up	2.403 down
kdpB	potassium-transporting atpase b chain	1.2	2.615 up	1.209 up	2.162 down
lmo0028	similar to *E. coli* microcin C7 self-immunity protein (MccF)	4.5	2.508 down	1.234 down	2.032 up
lmo0270	hypothetical protein	6.0	1.024 up	2.117 up	2.067 up
lmo0271	highly similar to phospho-beta-glucosidase	2.1.1	1.283 down	1.563 up	2.006 up
lmo0353	similar to unknown proteins	5.2	1.843 down	1.421 up	2.621 up
lmo0449	hypothetical protein	5.1	12.737 down	5.914 down	2.153 up
lmo0574	similar to beta-glucosidase	2.1.1	1.440 down	1.736 up	2.501 up
lmo0575	similar to transcription regulator GntR family	3.5.2	2.824 down	1.150 down	2.455 up
lmo0602	weakly similar to transcription regulator	3.5.2	1.856 up	4.190 up	2.256 up
lmo0800	similar to *B. subtilis* YqkB protein	5.2	1.558 down	1.290 up	2.011 up
lmo0850	hypothetical protein	6.0	12.029 down	4.912 down	2.448 up
lmo0901	similar to PTS system, cellobiose-specific IIC component	1.2	3.590 down	2.087 up	7.495 up
lmo0902	similar to transcription regulator (GntR family)	3.5.2	3.689 down	1.559 down	2.366 up
lmo0932	conserved hypothetical protein	5.2	2.077 down	1.003 up	2.085 up
lmo0937	hypothetical protein	6.0	4.352 down	1.879 down	2.315 up
lmo1516	similar to ammonium transporter NrgA	1.2	2.980 down	6.513 down	2.185 down
lmo1517	similar to nitrogen regulatory PII protein	2.2	3.125 down	6.582 down	2.105 down
lmo1779	hypothetical protein	6.0	22.031 down	10.238 down	2.151 up
lmo1798	similar to unknown protein	5.2	1.065 down	2.112 up	2.250 up
lmo1841	hypothetical protein	6.0	20.478 down	10.183 down	2.010 up
lmo1919	similar to unknown proteins	5.2	5.457 down	2.701 down	2.019 up
lmo2176	similar to transcriptional regulator (tetR family)	3.5.2	2.722 down	1.287 down	2.114 up
lmo2257	hypothetical CDS	6.0	1.573 down	1.452 up	2.284 up
lmo2765	similar to PTS cellobiose-specific enzyme IIA	1.2	2.263 down	1.016 up	2.300 up
lmo2816	similar to transport protein	1.2	9.502 up	4.300 up	2.209 down

<u>18 h Incubation</u>

name	description	Functional category	Fold change TA vs T0	Fold change DA vs T0	Fold change DA vs TA
atpI	highly similar to ATP synthase subunit i	1.4	3.811 down	8.110 down	2.127 down
lmo0342	similar to transketolase	2.1.2	1.508 up	3.677 up	2.438 up
lmo0343	similar to transaldolase	2.1.2	1.558 up	3.708 up	2.380 up
lmo0344	similar to dehydrogenase/reductase	2.1.1	3.672 up	9.234 up	2.514 up
lmo0345	similar to sugar-phosphate isomerase	2.1.1	1.528 up	3.468 up	2.269 up

lmo0735	similar to Ribulose-5-Phosphate 3-Epimerase	2.1.1	1.208 down	1.973 up	2.385 up
lmo0736	similar to ribose 5-phosphate isomerase	2.1.1	1.369 down	2.203 up	3.016 up
lmo0737	hypothetical protein	5.2	1.129 up	3.132 up	2.773 up
lmo0738	similar to phosphotransferase system (PTS) beta-glucoside-specific enzyme IIABC component	1.2	3.892 down	1.685 down	2.309 up
lmo0901	similar to PTS system, cellobiose-specific IIC component	1.2	2.955 down	1.289 down	2.291 up
lmo0985	hypothetical protein	6.0	1.364 up	2.923 up	2.143 up
lmo0986	similar to antibiotic ABC transporter, ATP-binding protein,	1.2	1.827 up	4.982 up	2.727 up
lmo0987	similar to *Streptococcus agalactiae* CylB protein	5.2	1.650 down	1.630 up	2.690 up
lmo1068	hypothetical protein lmo1068	6.0	1.097 up	2.346 up	2.138 up
lmo1799	putative peptidoglycan bound protein (LPXTG motif)	1.8	3.006 down	1.076 down	2.791 up
lmo2175	similar to dehydrogenase	2.1.1	3.096 up	9.370 up	3.026 up
lmo2254	similar to unknown proteins	5.2	14.388 down	4.606 down	3.123 up
lmo2799	similar to phosphotransferase system mannitol-specific enzyme IIBC	1.2	4.085 up	8.859 up	2.168 up
purE	hosphoribosylaminoimidazole carboxylase I	2.3	13.106 down	5.335 down	2.456 up
